# Association of Electronic Health Record Use Above Meaningful Use Thresholds With Hospital Quality and Safety Outcomes

**DOI:** 10.1001/jamanetworkopen.2020.12529

**Published:** 2020-09-09

**Authors:** Zachary R. Murphy, Jiangxia Wang, Michael V. Boland

**Affiliations:** 1 currently a medical student at Johns Hopkins University School of Medicine, Baltimore, Maryland; 2 Department of Biostatistics, Johns Hopkins University Bloomberg School of Public Health, Baltimore, Maryland; 3 Wilmer Eye Institute, Johns Hopkins University School of Medicine, Baltimore, Maryland; 4 Division of Health Sciences Informatics, Johns Hopkins University School of Medicine, Baltimore, Maryland

## Abstract

**Importance:**

By 2018, Medicare spent more than $30 billion to incentivize the adoption of electronic health records (EHRs), based partially on the belief that EHRs would improve health care quality and safety. In a time when most hospitals are well past minimum meaningful use (MU) requirements, examining whether EHR implementation beyond the minimum threshold is associated with increased quality and safety may guide the future focus of EHR development and incentive structures.

**Objective:**

To determine whether EHR implementation above MU performance thresholds is associated with changes in hospital patient satisfaction, efficiency, and safety.

**Design, Setting, and Participants:**

This quantile regression analysis of cross-sectional data used publicly available data sets from 2362 acute care hospitals in the United States participating in both the MU and Hospital Value-Based Purchasing (HVBP) programs from January 1 to December 31, 2016. Data were analyzed from August 1, 2019, to May 22, 2020.

**Exposures:**

Seven MU program performance measures, including medication and laboratory orders placed through the EHR, online health information availability and access rates, medication reconciliation through the EHR, patient-specific educational resources, and electronic health information exchange.

**Main Outcomes and Measures:**

The HVBP outcomes included patient satisfaction survey dimensions, Medicare spending per beneficiary, and 5 types of hospital-acquired infections.

**Results:**

Among the 2362 participating hospitals, mixed associations were found between MU measures and HVBP outcomes, all varying by outcome quantile and in some cases by interaction with EHR vendor. Computerized provider order entry (CPOE) for laboratory orders was associated with decreased ratings of every patient satisfaction outcome at middle quantiles (communication with nurses: β = −0.33 [*P* = .04]; communication with physicians: β = −0.50 [*P* < .001]; responsiveness of hospital staff: β = −0.57 [*P* = .03]; care transition performance: β = −0.66 [*P* < .001]; communication about medicines: β = −0.52 [*P* = .002]; cleanliness and quietness: β = −0.58 [*P* = .007]; discharge information: β = −0.48 [*P* < .001]; and overall rating: β = −0.95 [*P* < .001]). However, at middle quantiles, CPOE for medication orders was associated with increased ratings for communication with physicians (τ = 0.5; β = 0.54; *P* = .009), care transition (τ = 0.5; β = 1.24; *P* < .001), discharge information (τ = 0.5; β = 0.41; *P* = .01), and overall hospital ratings (τ = 0.5; β = 0.97; *P* = .02). At high quantiles, electronic health information exchange was associated with improved ratings of communication with nurses (τ = 0.9; β = 0.23; *P* = .03). Medication reconciliation had positive associations with increased communication with nursing at low quantiles (τ = 0.1; β = 0.60; *P* < .001), increased discharge information at middle quantiles (τ = 0.5; β = 0.28; *P* = .03), and responsiveness of hospital staff at middle (τ = 0.5; β = 0.77; *P* = .001) and high (τ = 0.9; β = 0.84; *P* = .001) quantiles. Patients accessing their health information online was not associated with any outcomes. Increased use of patient-specific educational resources identified through the EHR was associated with increased ratings of communication with physicians at high quantiles (τ = 0.9; β = 0.20; *P* = .02) and with decreased spending at low-spending hospitals (τ = 0.1; β = −0.40; *P* = .008).

**Conclusions and Relevance:**

Increasing EHR implementation, as measured by MU criteria, was not straightforwardly associated with increased HVBP measures of patient satisfaction, spending, and safety in this study. These results call for a critical evaluation of the criteria by which EHR implementation is measured and increased attention to how different EHR products may lead to differential outcomes.

## Key Points

**Question:**

Is electronic health record implementation beyond meaningful use thresholds associated with changes in hospital measures of patient satisfaction, spending, and safety?

**Findings:**

In this cross-sectional analysis of 2362 hospitals using data from 2016, associations between meaningful use performance measures and Hospital Value-Based Purchasing Program measures of patient satisfaction, spending, and safety were evaluated. Mixed associations were found that varied depending on whether the hospital was in the lower, middle, or upper quantiles of the Hospital Value-Based Purchasing Program outcome.

**Meaning:**

These findings suggest that advanced levels of electronic health record implementation are not consistently associated with patient satisfaction, spending, and safety, and in some cases depend on the outcome quantile.

## Teaser

This cross-sectional quality improvement study assesses whether electronic health record implementation above meaningful use thresholds is associated with changes in hospital patient satisfaction, efficiency, and safety.

## Introduction

The HITECH (Health Information Technology for Economic and Clinical Health) Act of 2009 was motivated by the belief that electronic health records (EHRs) would improve health care quality and safety.^1^ The HITECH Act created financial incentives for hospitals to demonstrate “meaningful use” (MU) of EHRs by meeting minimum implementation and performance thresholds across an array of EHR functions.

With more than $30 billion spent on the MU program (renamed *Promoting Interoperability*) by 2018,^2^ many studies have investigated whether the claim that EHRs would improve hospital quality and safety has paid off. This research has largely focused on comparisons between hospitals that attained the MU threshold and those that did not, which has revealed a divide between large, urban, academic hospitals that tended to achieve MU early and small, rural, nonacademic hospitals that lagged behind.^3,4,5^ Studies of patient satisfaction and the EHR in the inpatient setting have shown contradictory findings.^6,7,8,9,10,11,12,13,14^ Regarding cost control, attaining MU has not been found to affect expenditures per patient or hospital operating margins.^15,16^ The evidence that EHRs improve safety is stronger,^17^ but existing studies largely compared hospitals with full EHRs and hospitals with minimal or no EHRs.

In treating EHR implementation as a dichotomy between MU attained or not, little research has investigated differences between hospitals that just pass the minimum thresholds to meet MU and those that far exceed the minimum thresholds. Existing studies showed hospitals successfully attesting nearer to the minimum thresholds tended to be small, rural, nonacademic hospitals, whereas those at the top of the performance measures tended to be large, urban, academic medical centers.^18^ Furthermore, among hospitals attesting to MU, the EHR vendor had mixed associations with 6 MU performance measures.^19^

The heterogeneity of health systems, EHR products, and other factors contributing to the health care environment means that we must continually consider whether we are incentivizing the proper metrics to fully realize EHRs as a driver of quality and safety. In a time when most hospitals have EHR capabilities above the MU minimum thresholds, examining the association between EHR implementation above MU thresholds and quality and safety outcomes may provide insight into whether these MU metrics are still meeting their intended goal.

## Methods

This study used publicly available data sets and therefore did not fit the Health and Human Services criteria for human subjects research and did not require approval by an institutional review board or informed consent. eTable 1 in the Supplement contains links to the data sources used. This study followed the Strengthening the Reporting of Observational Studies in Epidemiology (STROBE) reporting guideline.

We created a cross-sectional sample of acute care hospitals that attested to participation in the MU program and also participated in the Hospital Value-Based Purchasing Program (HVBP) from January 1 to December 31, 2016, then constructed quantile regression models to examine associations between MU performance measures and 14 outcomes of the HVBP covering patient satisfaction, spending, and safety domains. Although the data represent 2016, this analysis was performed from August 1, 2019, to May 22, 2020.

### Outcomes: HVBP Quality and Safety Domain Scores

As measures of hospital patient satisfaction, efficiency, and safety, we used HVBP domain components. The HVBP is a Centers for Medicare & Medicaid (CMS) program that awards or penalizes acute care hospitals for safety and quality outcomes using payment adjustments,^20^ and data are publicly available through the Hospital Compare website.^21,22^ Hospitals receive domain scores, each composed of 1 to several components, some of which have changed over time. We have included brief descriptions of the components herein, and eTable 2 in the Supplement contains detailed descriptions.

The engagement domain reflects patient satisfaction and is derived from the Hospital Consumer Assessment of Healthcare Providers and Systems survey, which is sent to a subset of inpatients after hospital discharge to assess dimensions of satisfaction, including ratings of communication with nurses, communication with physicians, responsiveness of hospital staff, care transition, communication about medicines, cleanliness and quietness, discharge information, and the hospital overall. Each dimension is reported as the percentage of respondents selecting the best possible response for the relevant questions, adjusted for patient-level characteristics. Higher scores indicate better satisfaction.

The efficiency domain consists of a single measure, Medicare spending per beneficiary, which is reported as a ratio between the hospital’s mean price-standardized risk-adjusted spending per care episode divided by the national median of spending per episode. Lower scores indicate better efficiency.

The safety domain consists of several measures of in-hospital infections, accidents, and injuries. Among the components, the health care–associated infection measures are amenable to modeling. These reflect risk-adjusted standardized infection ratios for central line–associated bloodstream infections, catheter-associated urinary tract infections, surgical site infections (SSIs) after colon surgery, SSIs after abdominal hysterectomy, methicillin-resistant *Staphylococcus aureus* bacteremia, and *Clostridioides difficile* infection. These data are reported as ratios between observed and estimated infection rates. Lower scores indicate better safety.

The clinical care domain reflects 30-day all-cause mortality for 3 admission diagnoses. Although this domain is an important measure of hospital quality and safety, data for this domain have not been released for 2016, and thus we were unable to include it. The eMethods, eTable 3, and the eFigure in the Supplement contain a detailed discussion of our choice of outcomes that was constrained by frequent changes in both MU and outcome measures over time.

### MU Performance Measures

Each stage of MU has a set of EHR performance measures for which hospitals must submit data. However, these requirements changed over time, resulting in 4 overlapping sets of measures. We used CMS documentation to link identical measures across data sets, then found that 2016 was the best time frame to analyze.^23,24,25,26^ eMethods in the Supplement includes details. This resulted in 9 MU measures included as potential factors used to estimate performance (Table 1).

**Table 1.** MU Performance Measures

Measure	Numerator	Denominator
Electronic prescribing	Number of prescriptions in the denominator generated, queried for a drug formulary, and transmitted electronically	Number of new or changed permissible prescriptions written for drugs requiring a prescription to be dispensed for patients discharged during the EHR reporting period
Electronic access used	Number of patients (or patient-authorized representatives) in the denominator who view, download, or transmit to a third party their health information	Number of unique patients discharged from the inpatient or emergency department (POS 21 or 23) of the eligible hospital or CAH during the EHR reporting period
Electronic access available	Number of patients in the denominator who have access to view, download, and transmit their health information within 36 h after the information is available to the eligible hospital or CAH	Number of unique patients discharged from an eligible hospital’s or CAH’s inpatient or emergency department (POS 21 or 23) during the EHR reporting period
Medication reconciliation	Number of transitions of care in the denominator where medication reconciliation was performed	Number of transitions of care during the EHR reporting period for which the eligible hospital’s or CAH’s inpatient or emergency department (POS 21 or 23) was the receiving party of the transition
Electronic information exchange	Number of transitions of care and referrals in the denominator where a summary-of-care record was created using CEHRT and exchanged electronically	Number of transitions of care and referrals during the EHR reporting period for which the eligible hospital’s or CAH’s inpatient or emergency department (POS 21 or 23) was the transferring or referring provider
Patient-specific educational resources	Number of patients in the denominator who are subsequently provided patient-specific educational resources identified by CEHRT	Number of unique patients admitted to the eligible hospital or CAH inpatient or emergency departments (POS 21 or 23) during the EHR reporting period
CPOE for laboratory orders	Number of orders in the denominator recorded using CPOE	Number of laboratory orders created by the authorized providers in the eligible hospital’s or CAH’s inpatient or emergency department (POS 21 or 23) during the EHR reporting period
CPOE for medication orders	Number of orders in the denominator recorded using CPOE	Number of medication orders created by the authorized providers in the eligible hospital’s or CAH’s inpatient or emergency department (POS 21 or 23) during the EHR reporting period
CPOE for radiology orders	Number of orders in the denominator recorded using CPOE	Number of radiology orders created by the authorized providers in the eligible hospital’s or CAH’s inpatient or emergency department (POS 21 or 23) during the EHR reporting period

Abbreviations: CAH, critical access hospital; CEHRT, certified electronic health record technology; CPOE, computerized provider order entry; EHR, electronic health record; MU, meaningful use; POS, place of service code.

The MU attestation data are available through CMS public use files.^27^ The MU program also maintains a public file of the EHR products used by each hospital.^28^ Because some EHR vendors split software packages into separate products while others offer a unified product, we examined EHR use at the level of EHR vendor. We used this in conjunction with the Certified Health IT Product List, which contains information about the functionality of each EHR product, to profile the EHR functionality of each hospital.^29^ We used crosswalks published by CMS to combine the various versions of Certified Health IT Product List criteria into a unified set, then calculated the mean percentage of criteria met by each EHR vendor per hospital.^30^

### Hospital Characteristics

We controlled for hospital characteristics, including years of MU program participation, number of EHR vendors used, and the mean percentage of EHR product certification criteria met by each EHR vendor used by each hospital. We also adjusted for hospital characteristics, including ownership, location, and hospital identifiers from Hospital Compare data^22^; number of beds, inpatient revenue, payor mix, and teaching status from CMS cost reports^31^; case-mix index from CMS^32^; Magnet status^33^; and the urban-rural scale for US counties from the National Center for Health Statistics.^34^

### Statistical Analysis

Data for each hospital were linked using the CMS certification number. Because the Magnet program data set did not include CMS certification numbers, we manually matched each Magnet recipient to its CMS certification number.

Continuous variables (MU measures, HVBP outcomes, total inpatient revenue, Medicare and Medicaid discharge percentages, vendor count, and case-mix index) were examined for outliers greater than 3 SDs from the mean. In cases where outliers could be corroborated as data entry errors through hospital websites or American Hospital Directory Free Hospital Profiles (limited hospital profiles based on public and proprietary data),^35^ they were replaced with values from 2015 data. In cases where an outlier could not be confirmed as an entry error, it was retained. Records with missing independent and control variables were removed from analysis. Records with missing outcome data were removed from the specific model for that outcome, and baseline characteristics were compared with hospitals that were included using 2-sample *t* tests. Pairwise correlations less than 0.7 and variable inflation factors less than 10 were considered acceptable for performance measures.

Characteristics of the sample were summarized as means and SDs, median and interquartile range, or frequencies and percentages. The most frequently used EHR vendors were identified by examining how many hospitals used each vendor during 2016.

Quantile regression models were constructed for each outcome using the Statsmodels module, version 0.9.0, in Python, version 3.7 (Python Software Foundation). Quantile regression examines associations between variables used to estimate outcomes and a continuous outcome at different quantiles of the outcome.^36,37^ At each quantile τ, a model is produced with coefficients for each variable used to estimate outcomes, which allows us to examine different associations between the variables and the outcome as different levels of the outcome. Unit changes for all MU performance measures, percentage of Medicare/Medicaid discharges, and EHR product feature coverage were set at 10%. Unit changes for all outcomes were set at 1%. Interactions between each performance measure and the 4 most commonly used EHR vendors were included, as well as between EHR vendor and number of beds. We examined results at 3 quantiles (0.1, 0.5, and 0.9) for each outcome, selected a priori to represent low, middle, and high outcome performance. We used a Bonferroni correction to account for multiple outcomes by multiplying unadjusted *P* values by the number of outcomes (14) and reporting the 99.6% CIs. Corrected 2-sided *P* < .05 was considered significant.

## Results

### Sample Characteristics

A total of 2362 hospitals were included in the sample. Descriptive statistics are shown in Table 2.^38^ Three data entry errors were replaced with 2015 values (eTable 4 in the Supplement). We found that of the 165 EHR vendors used, the 4 most frequently used were Epic Systems Corporation (Epic; 585 [24.8%]), Meditech Information Technology, Inc (Meditech; 575 [24.3%]), Cerner Corporation (Cerner; 546 [23.1%]), and McKesson Corporation (McKesson; 283 [12.0%]).

**Table 2.** Characteristics of Hospitals Included in the Analysis

Variable	Data (n = 2362)
CPOE for medication orders (by order), mean (SD), %	85.3 (10.4)
CPOE for laboratory orders, mean (SD), %	79.9 (15.1)
Patient electronic access (available), mean (SD), %	89.7 (12.0)
Patient electronic access (accessed), mean (SD), %	12.4 (13.7)
Patient-specific educational resources, mean (SD), %	73.8 (24.3)
Medication reconciliation, mean (SD), %	88.2 (11.9)
Electronic health information exchange, mean (SD), %	38.7 (22.1)
Top EHR use, No. (%) of hospitals	
Meditech Information Technology, Inc	575 (24.3)
Epic Systems Corporation	585 (24.8)
Cerner Corporation	546 (23.1)
McKesson Corporation	283 (12.0)
Years of MU attestation, mean (SD)	4.7 (0.9)
Hospital ownership, No. (%)	
Government	343 (14.5)
Nonprofit	1517 (64.2)
Proprietary	502 (21.3)
Geographic division, No. (%)	
East North Central	408 (17.3)
East South Central	190 (8.0)
Mid-Atlantic	300 (12.7)
Mountain	153 (6.5)
New England	108 (4.6)
Pacific	251 (10.6)
South Atlantic	433 (18.3)
West North Central	192 (8.1)
West South Central	327 (13.8)
No. of beds, No. (%)	
1-99	647 (27.4)
100-199	769 (32.6)
200-299	394 (16.7)
300-399	235 (9.9)
400-499	120 (5.1)
≥500	197 (8.3)
Total inpatient revenue, mean (SD), $1 million	603.3 (866.0)
Medicare discharges, mean (SD), %	34.8 (10.8)
Medicaid discharges, mean (SD), %	10.5 (10.0)
Teaching hospital, No. (%)	947 (40.1)
NCHS code, No. (%)	
Large central metropolitan	556 (23.5)
Large fringe metropolitan	480 (20.3)
Medium metropolitan	485 (20.5)
Small metropolitan	296 (12.5)
Micropolitan	404 (17.1)
Noncore	141 (6.0)
Vendor count, median (IQR)	2 (1-2)
Criteria coverage, mean (SD), %	68.1 (17.0)
Magnet, No. (%)	250 (10.6)
CMI, mean (SD)	1.6 (0.3)
Communication with nurses, mean (SD), %	78.6 (4.3)
Communication with physicians, mean (SD), %	80.0 (4.1)
Responsiveness of hospital staff, mean (SD)	64.7 (6.7)
Care transition, mean (SD)	50.5 (5.8)
Communication about medicines, mean (SD)	63.1 (4.9)
Cleanliness and quietness, mean (SD)	65.4 (6.2)
Discharge information, mean (SD)	87.0 (3.3)
Overall rating of hospital, mean (SD)	70.7 (7.4)
MSPB, mean (SD)	1.0 (0.1)
CLABSI, mean (SD)	0.5 (0.5)
CAUTI, mean (SD)	0.5 (0.5)
SSI after colon surgery, mean (SD)	0.9 (0.7)
MRSA bacteremia, mean (SD)	1.0 (0.8)
*Clostridioides difficile* infection, mean (SD)	0.8 (0.4)

Abbreviations: CAUTI, catheter-associated urinary tract infection; CLABSI, central line–associated bloodstream infection; CMI, case-mix index; CPOE, computerized provider order entry; EHR, electronic health record; IQR, interquartile range; MRSA, methicillin-resistant *Staphylococcus aureus*; MSPB, Medicare spending per beneficiary; MU, meaningful use; NCHS, National Center for Health Statistics; SSI, surgical site infection.

### Quantile Regression

Computerized provider (physicians and nonphysician licensed clinicians) order entry (CPOE) for laboratory orders and CPOE for radiology orders were highly correlated (Pearson correlation, 0.76), so the latter was excluded. Among all the other variables used to estimate outcomes, all pairwise correlations and variable inflation factors values were within acceptable ranges. Only 1365 hospitals (57.8%) submitted data for the electronic prescribing measure, so this outcome was omitted. Only 729 hospitals (30.8%) submitted data for SSI after abdominal hysterectomy, and therefore this outcome was omitted. Hospitals with missing health care–associated infection outcome data had MU measures that were significantly different from those that were included in the models (eTable 5 in the Supplement).

Table 3 contains adjusted regression coefficients for performance measures at the 10th, 50th, and 90th percentiles. eTables 6 and 7 in the Supplement contain complete adjusted results.

**Table 3.** Adjusted Regression Results for MU Performance Measures at 0.1, 0.5, and 0.9 Quantiles^a^

Variable	τ Statistic	Patient satisfaction, % selecting the best possible response								Efficiency, MSPB (n = 2362)^b^	Safety, standardized infection ratio^c^				
		Communication with nurses (n = 2362)	Communication with physicians (n = 2362)	Responsiveness of hospital staff (n = 2362)	Care transition (n = 2362)	Communication about medicines (n = 2362)	Clean and quiet (n = 2362)	Discharge information (n = 2362)	Overall rating (n = 2362)		CLABSI (n = 1642)	CAUTI (n = 1856)	SSI after colon surgery (n = 1689)	MRSA bacteremia (n = 1510)	*Clostridioides difficile* Infection (n = 2342)
CPOE for medication orders	0.1	0	0.01	0.71	0.49	0.47	0.01	0.47	–0.02	–0.44	0.01	0.1	0.78	2.23	2.87
	0.5	0.43	0.54^d^	0.74	1.24^e^	0.39	0.61	0.41^f^	0.97^f^	–0.57	–1.71	3.73	–3.6	0.79	2.75
	0.9	0.22	0.37	0.53	0.41	0.70	0.66	0	0.66	–0.49	–4	5.48	–10.59	–3.61	–2.33
CPOE for laboratory orders	0.1	–0.33	–0.26	–0.53	–0.42	–0.53	–0.12	–0.85^e^	–0.56	0.21	0.04	0.06	2.53	–0.47	–0.49
	0.5	–0.33^f^	–0.50^e^	–0.57^f^	–0.66^e^	–0.52^d^	–0.58^d^	–0.48^e^	–0.95^e^	0.02	3.1	–2.65	1.64	1.76	–1.96
	0.9	–0.12	–0.36^f^	–0.17	–0.14	–0.31	–0.15	–0.12	–0.65	–0.14	–0.13	–0.5	6.37	6.74	0.76
Patient electronic access (available)	0.1	0.45	0.25	0.30	–0.50	0.10	0.20	–0.19	0.28	–0.03	–0.30	–0.11	2.71	–1.12	1.19
	0.5	0.01	–0.12	–0.18	–0.16	0.12	–0.04	0.16	–0.08	0.18	–5.24^f^	2.02	4.95	3.79	0.55
	0.9	–0.02	–0.30	0	–0.07	0.02	–0.07	0.01	0.13	–0.08	4.69	–5.41	16.86^d^	1.56	0.06
Patient electronic access (used)	0.1	–0.22	0.39	–0.51	–0.10	0.24	0.02	0.38	0.12	–0.07	–0.08	0.01	4.84	4.37	0.45
	0.5	–0.15	–0.21	0.06	–0.20	0.07	–0.23	–0.06	–0.18	–0.42	3.08	1.52	0.03	–2.4	–2.35
	0.9	–0.07	0.17	–0.30	0.19	–0.06	–0.33	–0.02	0.05	–0.71	–1.72	0.16	15.26	0.52	–4.29
Patient-specific educational resources	0.1	–0.08	0.04	–0.08	0.07	0.07	–0.06	0.09	–0.23	–0.40^d^	0	–0.03	–0.41	–1.33	–1.13
	0.5	0.01	0.08	0.21	0.19	0.18	0.24	0.02	0.29	–0.13	–0.68	–0.44	2.06	–2.01	–0.53
	0.9	0.03	0.20^f^	0.29	0.16	0.18	0.24	0.05	0.21	0.17	3.04	–0.18	–5.72	–4.64	–0.93
Medication reconciliation	0.1	0.60^e^	0.36	0.58	–0.23	0.38	–0.01	–0.03	0.04	0.29	–0.04	0.02	–0.83	0.04	–2.09
	0.5	0.33	0.23	0.77^d^	–0.18	0.38	0.37	0.28^f^	0.06	0.03	4.10	0.42	–2.73	4.67	–2.56
	0.9	0.45^f^	–0.01	0.84^d^	0.17	0.42	0.44	–0.05	0.40	–0.03	–5.10	–1.91	8.93	2.42	3.08
Electronic health information exchange	0.1	0.10	–0.06	0.18	0.16	–0.03	–0.23	0.15	–0.13	–0.29	0.12	0.06	0.01	–0.99	–0.58
	0.5	0.10	0.05	0.19	–0.06	–0.02	–0.01	–0.03	0.07	–0.11	1.29	–0.68	0.77	0.50	1.60
	0.9	0.24^f^	0.02	0.56^e^	0.04	0.03	0.30	–0.12	0.21	–0.04	5.34^e^	2.73	4.47	6.77	–0.09

Abbreviations: CAUTI, catheter-associated urinary tract infection; CLABSI, central line–associated bloodstream infection; CPOE, computerized provider order entry; MRSA, methicillin-resistant *Staphylococcus aureus*; MSPB, Medicare spending per beneficiary; MU, meaningful use; SSI, surgical site infection.

^a^ Complete regression results, including exact *P* values and 99.6% CIs, are given in eTable 5 in the Supplement. Models were adjusted for years of MU attestation, hospital ownership, geographic division, number of beds, total inpatient revenue, payor mix, teaching status, National Center for Health Statistics urban-rural code, electronic health record (EHR) vendor count, use of specific EHR vendors, EHR functionality, Magnet status, and case-mix index as well as interactions between MU performance measure with EHR vendor and between EHR vendors with number of beds.

^b^ Reported as the ratio between the hospital’s mean price-standardized risk-adjusted spending per care episode divided by the national median of spending per episode. Lower scores indicate better efficiency.

^c^ Reported as ratios between observed and estimated infection rates. Lower scores indicate better safety.

^d^ *P* < .01.

^e^ *P* < .001.

^f^ *P* < .05.

Computerized provider order entry for laboratory orders was associated with decreased performance on every patient satisfaction outcome at middle quantiles (Table 3). However, these decreases were not present in the discharge information outcome for hospitals using McKesson (interaction: τ = 0.5; β = 0.47; *P* = .006) or Meditech (interaction: τ = 0.5; β = 0.46; *P* = .02). Computerized physician order entry for medication orders was associated with improved communication with physicians (τ = 0.5; β = 0.54; *P* = .009), care transition (τ = 0.5; β = 1.24; *P* < .001), discharge information (τ = 0.5; β = 0.41; *P* = .01), and overall hospital ratings (τ = 0.5; β = 0.97; *P* = .02) at middle quantiles.

At high quantiles, electronic health information exchange was associated with improved communication with nurses (τ = 0.9; β = 0.23; *P* = .03) and responsiveness of hospital staff (τ = 0.9; β = 0.56; *P* < .001), but also with increased rates of central line–associated bloodstream infections (τ = 0.9; β = 5.23; *P* = .03).

Medication reconciliation was associated with increased communication with nursing at low quantiles (τ = 0.1; β = 0.60; *P* < .001), increased discharge information at middle quantiles (τ = 0.5; β = 0.28; *P* = .03), and increased responsiveness of hospital staff at middle (τ = 0.5; β = 0.77; *P* = .001) and high (τ = 0.9; β = 0.84; *P* = .001) quantiles. However, the concurrent use of Epic was associated with a reverse in these associations wherein increased medication reconciliation was associated with decreased communication with nursing at low quantiles (interaction: τ = 0.1; β = −1.19; *P* = .005) and decreased responsiveness of staff ratings at middle quantiles (interaction: τ = 0.5; β = −1.37; *P* = .02).

Patients accessing their information online was not significantly associated with any outcome. However, having patients’ health information online, whether accessed or not, was associated with an increase in SSIs after colon surgery at high quantiles (τ = 0.9; β = 12.45; *P* = .03).

Patient-specific educational resources were associated with increased communication with physicians at high quantiles (τ = 0.9; β = 0.20; *P* = .02); however, a reverse association was found with concurrent use of Cerner (interaction: τ = 0.9; β = −0.36; *P* = .02) or McKesson (interaction: τ = 0.9; β = −0.36; *P* = .02). In addition, patient-specific educational resources were associated with decreased spending at low spending hospitals (τ = 0.1; β = −0.40; *P* = .008).

## Discussion

This study is the first of which we are aware to assess whether EHR implementation above MU thresholds is associated with HVBP outcomes. Our results suggest that EHR use above minimal MU requirements has small, mixed associations with HVBP engagement, efficiency, and safety outcomes that in some cases depend on the EHR vendor.

Although increased use of CPOE for medications was associated with improved patient satisfaction in some areas, increased CPOE for laboratory tests was associated with lower satisfaction in all areas. This finding suggests that studies of CPOE must look at these distinct order types rather than CPOE as a single entity. Although CPOE for medication and laboratory orders is commonly unified by the EHR, the workflows for each activity diverge almost immediately. Systems factors beyond the CPOE system may contribute to these opposing associations, and more research is therefore necessary to explain these findings.

Our finding of no association between patients accessing their information online and cost savings is consistent with past research.^16^ Regarding our finding that patients’ information being online, whether accessed or not, was associated with an increase in SSIs after colon surgery is most likely the result of an unidentified confounding variable, because no straightforward theory as to why these would be associated appears to exist.

Past research has shown electronic health information exchange to be associated with better patient satisfaction and cost control.^39,40^ Although we did not find associations with cost savings, we did find positive associations with patient satisfaction, in particular communication with nurses and responsiveness of hospital staff. Communication with nursing is vital at admission and discharge, and increased electronic transmission of health records may facilitate data gathering and nurse-patient communication that occurs during these times, resulting in higher ratings of communication.

Past research^41^ has examined medication reconciliation and patient satisfaction as independent outcomes in the context of transition of care interventions, but no past research has looked at specific associations between medication reconciliation performed through the EHR and patient satisfaction. We found medication reconciliation was associated with several dimensions of patient satisfaction related to admission and discharge, when medication reconciliation would be performed. However, these associations were scattered among low, medium, and high quantiles. It is unclear why these associations were not more consistent across patient satisfaction dimensions and across quantiles. Moreover, past research^42^ has found cost savings associated with pharmacist-led interventions involving medication reconciliation, so we were surprised to not find this association at any quantile.

Identifying patient educational information through the EHR was associated with higher ratings of communication with physicians at high quantiles, but only associated with decreased Medicare spending per beneficiary at low-spending hospitals. Physicians with high communication skills may be more adept at using this information through the EHR, so only highly rated communicators might see benefits from using this information. Similarly, cost savings may only be seen with increased use of educational information found through the EHR at low-spending hospitals because less efficient hospitals may not have structures and workflows to use these tools as efficiently. Further research is necessary to explore these results.

Several of our results are associated with significant interaction terms based on EHR vendor, which either removed or reversed the main effects. This finding suggests that the particular solutions offered may differentiate by vendor and warrant further study.

Taken together, our results suggest that the MU performance measures used thus far do not straightforwardly estimate HVBP measures of patient satisfaction, efficiency, or safety. Although stage 2 of MU is largely in the past, stage 3 is the present for hospitals, and many of the performance measures for stage 3 are the same as those considered herein.^43^ Our results suggest that the current criteria may not be focusing on the right metrics to improve patient satisfaction, efficiency, and some measures of safety as measured by HVBP at all hospitals.

### Strengths and Limitations

Strengths of our study include a large sample size and the use of quantile regression to explore associations at different levels of the outcomes. There are also several limitations. Owing to changes in the measures used, our time frame is limited to 2016 and reflects only part of the HVBP safety domain and none of the clinical care domain. We may not have been able to include some relevant factors in our models. Of note, the MU program only collects data about certified EHR technology, and thus our analysis does not take into account the potential effects of using noncertified or non-EHR systems. Moreover, there are previously described limitations to the validity of HVBP domains as measures of patient satisfaction and cost control.^44,45^ Our sample was limited to acute care hospitals in the United States because only they were eligible for the HVBP program, excluding many rural and critical access hospitals, which historically have struggled to implement EHR technology.^46^ Moreover, much of the data analyzed are self-submitted by hospitals, which may be a source of bias and error. In particular, our findings regarding health care–associated infection outcomes may not be generalizable because the MU measures of hospitals included in the models were different from those in the hospitals excluded because they did not submit data, and hospitals may not have submitted data for particular measures owing to low performance.

## Conclusions

Although some MU performance measures were significantly associated with patient satisfaction, efficiency, and safety, most associations varied by the level of the outcomes. Moreover, the EHR vendor was an important interacting factor in several of our findings. Insofar as the MU program was founded on the belief that more EHR implementation will lead to better quality and safety, these results call for a critical evaluation of the criteria by which EHR implementation is measured and incentivized, as well as increased attention to understanding how the different features of EHR solutions may lead to differential outcomes.

## Notes

Supplement. **eTable 1.** Data Sources Used **eTable 2.** HVBP Outcomes Detailed **eTable 3.** Number of Hospitals Attesting to Each Meaningful Use Performance Measure by Year **eTable 4.** Errors Fixed **eTable 5.** Mean Differences (*P* Value) in MU Measures Between Hospitals Included in HAI Models vs Hospitals Excluded Due to Not Submitting Data Using 2-Sample *t* Test **eTable 6.** Adjusted Quantile Regression Results for HVBP Engagement (Patient Satisfaction) Outcomes at 0.1, 0.5, and 0.9 Quantiles **eTable 7.** Adjusted Quantile Regression Results for Medicare Spending per Beneficiary (MSPB) and Hospital-Acquired Infection (HAI) Outcomes at 0.1, 0.5, and 0.9 Quantiles **eMethods.** Data Considerations **eFigure.** Hospital Value-Based Purchasing Program (HVBP) Domain Component Performance Periods by Fiscal Year
