# Supplementary material for: Association of Electronic Health Record Use Above Meaningful Use Thresholds With Hospital Quality and Safety Outcomes
Source: JAMA Netw Open. 2020 Sep 9;3(9):e2012529. doi: 10.1001/jamanetworkopen.2020.12529 (PMC7489820; doi:10.1001/jamanetworkopen.2020.12529)
Supplement: Supplement. — eTable 1. Data Sources Used eTable 2. HVBP Outcomes Detailed eTable 3. Number of Hospitals Attesting to Each Meaningful Use Performance Measure by Year eTable 4. Errors Fixed eTable 5. Mean Differences (P Value) in MU Measures Between Hospitals Included in HAI Models vs Hospitals Excluded Due to Not Submitting Data Using 2-Sample t Test eTable 6. Adjusted Quantile Regression Results for HVBP Engagement (Patient Satisfaction) Outcomes at 0.1, 0.5, and 0.9 Quantiles eTable 7. Adjusted Quantile Regression Results for Medicare Spending per Beneficiary (MSPB) and Hospital-Acquired Infection (HAI) Outcomes at 0.1, 0.5, and 0.9 Quantiles eMethods. Data Considerations eFigure. Hospital Value-Based Purchasing Program (HVBP) Domain Component Performance Periods by Fiscal Year [file jamanetwopen-e2012529-s001.pdf]

Supplementary Online Content

Murphy ZR, Wang J, Boland MV. Association of electronic health record use above meaningful use thresholds with hospital quality and safety outcomes. *JAMA Netw Open*. 2020;3(9):e2012529. doi:10.1001/jamanetworkopen.2020.12529

eTable 1. Data Sources Used
eTable 2. HVBP Outcomes Detailed
eTable 3. Number of Hospitals Attesting to Each Meaningful Use Performance Measure by Year
eTable 4. Errors Fixed
eTable 5. Mean Differences (*P* Value) in MU Measures Between Hospitals Included in HAI Models vs Hospitals Excluded Due to Not Submitting Data Using 2-Sample *t* Test
eTable 6. Adjusted Quantile Regression Results for HVBP Engagement (Patient Satisfaction) Outcomes at 0.1, 0.5, and 0.9 Quantiles
eTable 7. Adjusted Quantile Regression Results for Medicare Spending per Beneficiary (MSPB) and Hospital-Acquired Infection (HAI) Outcomes at 0.1, 0.5, and 0.9 Quantiles
eMethods. Data Considerations
eFigure. Hospital Value-Based Purchasing Program (HVBP) Domain Component Performance Periods by Fiscal Year

This supplementary material has been provided by the authors to give readers additional information about their work.

©2020 Murphy ZR et al. *JAMA Network Open*.

eTable 1. Data Sources Used

**Data source**	**Version/Year**	**URL**
Meaningful use public use files	Stage 1 for eligible hospitals	https://www.cms.gov/Regulations-and-Guidance/Legislation/EHRIncentivePrograms/Downloads/EH_PUF_Q32018Stage1.zip
	Stage 2 for eligible hospitals	https://www.cms.gov/Regulations-and-Guidance/Legislation/EHRIncentivePrograms/Downloads/EH_PUF_Q32018Stage2.zip
Hospital Compare	Revised flat files 10/31/2018	https://medicare.gov/download/HospitalCompare/2018/October/HOSArchive_Revised_FlatFiles_20181031.zip
CMI	FY 2018 Final	https://www.cms.gov/Medicare/Medicare-Fee-for-Service-Payment/AcuteInpatientPPS/Downloads/FY2018-CMS-1677-FR-Case-Mix.zip
CMS cost reports	FY 2016	http://downloads.cms.gov/FILES/HCRIS/HOSP10FY2016.zip
EHR products used for Meaningful Use attestation	Complete dataset	https://dashboard.healthit.gov/datadashboard/data/MU_REPORT.csv
CHPL	2011 edition	https://chpl.healthit.gov/rest/download?api_key=12909a978483dfb8ecd0596c98ae9094&edition=2011
	2014 edition	https://chpl.healthit.gov/rest/download?api_key=12909a978483dfb8ecd0596c98ae9094&edition=2014
	2015 edition	https://chpl.healthit.gov/rest/download?api_key=12909a978483dfb8ecd0596c98ae9094&edition=2015
Census Geographic Division Codes	2010 census	https://www2.census.gov/geo/pdfs/maps-data/maps/reference/us_regdiv.pdf
Magnet	2019	https://www.nursingworld.org/MapOrganizationBlock/DownloadFormatMapData?type=Magnet
NCHS	2013 edition	https://www.cdc.gov/nchs/data/data_acces_files/NCHSURCodes2013.xlsx

CMI = case-mix index, CMS = centers for Medicare and Medicaid, EHR = electronic health record, CHPL = certified health IT product list, NCHS = national center for health statistics.

©2020 Murphy ZR et al. *JAMA Network Open*.

eTable 2. HVBP Outcomes Detailed

Domain	Measure	Description
Engagement (patient satisfaction)	Communication with Nurses	Patient-rated quality of communication with nurses. Reported as percent select best possible response adjusted for patient-mix.
	Communication with Doctors	Patient-rated quality of communication with doctors. Reported as percent select best possible response adjusted for patient-mix.
	Responsiveness of Hospital Staff	Patient-rated frequency of needing to wait for hospital staff to respond to requests. Reported as percent select best possible response adjusted for patient-mix.
	Care Transition	Patient-rated quality of information and understanding of care plan after discharge. Reported as percent select best possible response adjusted for patient-mix.
	Communication about Medicines	Patient-rated quality of information about new medications, including side effects, during inpatient stay. Reported as percent select best possible response adjusted for patient-mix.
	Cleanliness and Quietness of Hospital Environment	Patient-rated cleanliness and quietness of the hospital environment. Reported as percent select best possible response adjusted for patient-mix.
	Discharge Information	Patient-rated quality of information about help needed after discharge and signs to look out for. Reported as percent select best possible response adjusted for patient-mix.
	Overall Rating of Hospital	Patient-rated overall quality of the hospital. Reported as percent select best possible response adjusted for patient-mix.
Efficiency (spending)	Medicare Spending Per Beneficiary (MSPB)	Each hospital's price-standardized risk-adjusted Medicare spending per beneficiary per care episode divided by the national median Medicare spending per beneficiary per care episode. Reported as ratio, interpretable as spending percentage more or less than national median.
Safety	CLABSI (HAI-1)	Central line associated blood stream infections (CLABSIs). Reported as standardized infection rate: ratio of number of infections divided by the number of predicted infections based on national multivariate regression analyses. Interpretable as percent more or less than expected based on national trends.
	CAUTI (HAI-2)	Catheter associated urinary tract infections (CAUTIs). Reported as standardized infection rate: ratio of number of infections divided by the number of predicted infections based on national multivariate regression analyses. Interpretable as percent more or less than expected based on national trends.
	SSI-Colon (HAI-3)	Surgical site infections (SSIs) after colon surgery. Reported as standardized infection rate: ratio of number of infections divided by the number of predicted infections based on national multivariate regression analyses. Interpretable as percent more or less than expected based on national trends.
	SSI-Abd Hyst (HAI-4)	Surgical site infections (SSIs) after abdominal hysterectomy. Reported as standardized infection rate: ratio of number of infections divided by the number of predicted infections based on national multivariate regression analyses. Interpretable as percent more or less than expected based on national trends.
	MRSA Bacteremia (HAI-5)	Methicillin resistant staph aureus bacteremia. Reported as standardized infection rate: ratio of number of infections divided by the number of predicted infections based on national multivariate regression analyses. Interpretable as percent more or less than expected based on national trends.
	C. diff Infection (HAI-6)	Clostridioides difficile infection. Reported as standardized infection rate: ratio of number of infections divided by the number of predicted infections based on national multivariate regression analyses. Interpretable as percent more or less than expected based on national trends.

©2020 Murphy ZR et al. *JAMA Network Open*.

eTable 3. Number of Hospitals Attesting to Each Meaningful Use Performance Measure by Year

**Measure**	**2011**	**2012**	**2013**	**2014**	**2015**	**2016**
CPOE for Medication Orders (by patient)	807	2548	3926	2160	82	0
Maintain Problem List	807	2548	3926	2160	82	0
Active Medication List	807	2548	3926	2160	82	0
Medication Allergy List	807	2548	3926	2160	82	0
Record Demographics	807	2548	3926	3979	82	0
Record Vital Signs	807	2548	3926	3979	82	0
Record Smoking Status	803	2538	3903	3961	80	0
Electronic Copy of Health Information	236	845	1272	1853	82	0
Electronic Copy of Discharge Instructions	322	967	1276	71	0	0
CPOE for Medication Orders (by order)	0	0	0	1819	3957	3367
CPOE for Radiology Orders	0	0	0	1819	3754	3364
CPOE for Laboratory Orders	0	0	0	1819	3758	3365
Clinical Lab Test Results	0	0	0	1819	0	0
Patient Electronic Access (Available)	0	0	0	1819	3957	3367
Patient Electronic Access (Accessed)	0	0	0	1812	3711	3345
Patient-Specific Education Resources	0	0	0	1819	3877	3367
Medication Reconciliation	0	0	0	1819	3873	3367
Transition of Care Summary Provided	0	0	0	1818	0	0
Electronic Health Information Exchange	0	0	0	1818	3695	3367
Electronic Medication Administration Record (eMAR)	0	0	0	1702	0	0
ePrescriptions	0	0	0	0	1597	1996
Total unique	807	2548	3926	3979	4039	3367

CPOE = computerized provider order entry

©2020 Murphy ZR et al. *JAMA Network Open*.

eTable 4. Errors Fixed

CCN	Variable	Reported Value	2015 Value
100314	Num Beds	36985	133
500019	Num Beds	17924	86
521357	Num Beds	3716	25

©2020 Murphy ZR et al. *JAMA Network Open*.

eTable 5. Mean Differences (*P* Value) in MU Measures Between Hospitals Included in HAI Models vs Hospitals Excluded Due to Not Submitting Data Using 2-Sample *t* Test

**MU Measure**	**CLABSI**	**CAUTI**	**SSI-Colon**	**MRSA**	**C diff**
CPOE for Medication Orders	-2.1 (0.002)	-1.5 (0.044)	-3.2 (0.000)	-2.7 (0.000)	-6.7 (0.047)
CPOE for Laboratory Orders	-1.8 (0.000)	-1.3 (0.010)	-2.0 (0.000)	-2.2 (0.000)	-5.1 (0.028)
Electronic Health Information Exchange	2.6 (0.007)	4.1 (0.000)	0.1 (0.946)	2.0 (0.038)	2.1 (0.668)
Medication Reconciliation	-1.2 (0.026)	-0.5 (0.394)	-2.1 (0.000)	-1.6 (0.001)	-1.5 (0.576)
Patient Electronic Access (Accessed)	-2.0 (0.001)	-0.8 (0.234)	-2.8 (0.000)	-1.9 (0.001)	0.1 (0.982)
Patient Electronic Access (Available)	-2.3 (0.000)	-1.6 (0.006)	-2.4 (0.000)	-1.4 (0.006)	-3.8 (0.157)
Patient-Specific Education Resources	4.1 (0.000)	5.3 (0.000)	3.0 (0.007)	3.4 (0.001)	10.0 (0.068)

CPOE = computerized provider order entry, CLABSI = central line associated blood stream infection, SSI-Colon = surgical site infection following colon surgery, MRSA = methicillin resistant staph aureus, C diff = Clostridioides difficile.

©2020 Murphy ZR et al. *JAMA Network Open*.

eTable 6. Adjusted Quantile Regression Results for HVBP Engagement (Patient Satisfaction) Outcomes at 0.1, 0.5, and 0.9 Quantiles

Variable	τ	Communication with Nurses Performance Rate			Communication with Doctors Performance Rate			Responsiveness of Hospital Staff Performance Rate			Care Transition Performance Rate			Communication about Medicines Performance Rate			Cleanliness and Quietness of Hospital Environment Performance Rate			Discharge Information Performance Rate			Overall Rating of Hospital Performance Rate		
		**β**	**99.6% CI**	**p**	**β**	**99.6% CI**	**p**	**β**	**99.6% CI**	**p**	**β**	**99.6% CI**	**p**	**β**	**99.6% CI**	**p**	**β**	**99.6% CI**	**p**	**β**	**99.6% CI**	**p**	**β**	**99.6% CI**	**p**
CPOE for Medication Orders (by order)	0.1	0.00	-0.63 - 0.63	1.00	0.01	-0.48 - 0.5	1.00	0.71	-0.22 - 1.64	0.36	0.49	-0.28 - 1.26	0.91	0.47	-0.26 - 1.19	0.83	0.01	-0.75 - 0.77	1.00	0.47	-0.07 - 1.01	0.17	-0.02	-1.14 - 1.11	1.00
	0.5	0.43	-0.04 - 0.89	0.11	0.54**	0.08 - 1.00	0.01	0.74	-0.04 - 1.51	0.08	1.24***	0.60 - 1.88	0.00	0.39	-0.19 - 0.96	0.69	0.61	-0.11 - 1.32	0.18	0.41*	0.05 - 0.77	0.01	0.97*	0.07 - 1.86	0.02
	0.9	0.22	-0.43 - 0.87	1.00	0.37	-0.16 - 0.9	0.59	0.53	-0.38 - 1.44	1.00	0.41	-0.37 - 1.18	1.00	0.70	-0.04 - 1.45	0.08	0.66	-0.22 - 1.54	0.41	0.00	-0.36 - 0.37	1.00	0.66	-0.34 - 1.66	0.76
:MEDITECH	0.1	-0.43	-1.42 - 0.55	1.00	-0.25	-1.06 - 0.56	1.00	-1.03	-2.57 - 0.51	0.71	-0.99	-2.19 - 0.22	0.24	-0.76	-1.87 - 0.34	0.61	0.06	-1.13 - 1.24	1.00	-0.30	-1.21 - 0.62	1.00	-1.02	-2.77 - 0.73	1.00
	0.5	-0.09	-0.77 - 0.6	1.00	-0.41	-1.08 - 0.27	1.00	-0.81	-1.94 - 0.32	0.52	-1.48***	-2.41 - -0.54	0.00	-0.14	-0.98 - 0.7	1.00	-1.02	-2.06 - 0.03	0.06	-0.13	-0.66 - 0.39	1.00	-0.83	-2.15 - 0.48	0.89
	0.9	-0.13	-0.93 - 0.67	1.00	-0.61	-1.34 - 0.13	0.22	0.29	-0.92 - 1.51	1.00	-0.72	-1.72 - 0.29	0.52	-0.26	-1.22 - 0.7	1.00	-0.84	-2.17 - 0.48	0.87	0.62**	0.12 - 1.11	0.00	-0.56	-1.88 - 0.77	1.00
:Epic	0.1	1.68	0 - 3.36	0.05	1.94***	0.69 - 3.20	0.00	-0.63	-2.47 - 1.21	1.00	1.40	-0.4 - 3.2	0.33	0.95	-0.91 - 2.81	1.00	1.30	-0.78 - 3.38	0.96	0.54	-0.79 - 1.87	1.00	0.97	-1.88 - 3.82	1.00
	0.5	0.27	-0.79 - 1.33	1.00	0.52	-0.53 - 1.56	1.00	-0.63	-2.38 - 1.12	1.00	-0.86	-2.31 - 0.58	1.00	0.27	-1.03 - 1.58	1.00	0.44	-1.17 - 2.05	1.00	0.46	-0.35 - 1.28	1.00	-0.35	-2.37 - 1.68	1.00
	0.9	-0.25	-1.82 - 1.31	1.00	0.26	-0.89 - 1.41	1.00	-0.97	-3.49 - 1.54	1.00	-1.65	-3.46 - 0.16	0.11	-1.56*	-3.07 - -0.06	0.04	-0.31	-2 - 1.38	1.00	0.07	-0.7 - 0.84	1.00	0.52	-1.82 - 2.86	1.00
:Cerner	0.1	-0.41	-1.54 - 0.72	1.00	-0.14	-1 - 0.71	1.00	-0.65	-2.13 - 0.83	1.00	-0.63	-1.96 - 0.69	1.00	-0.89	-2.07 - 0.3	0.41	-0.27	-1.53 - 0.99	1.00	-0.23	-1.04 - 0.58	1.00	-0.44	-2.26 - 1.39	1.00
	0.5	0.05	-0.72 - 0.82	1.00	-0.29	-1.05 - 0.47	1.00	-0.09	-1.36 - 1.18	1.00	-0.64	-1.68 - 0.41	1.00	0.34	-0.6 - 1.29	1.00	0.24	-0.93 - 1.41	1.00	-0.48	-1.07 - 0.11	0.24	-0.55	-2.02 - 0.92	1.00
	0.9	-0.41	-1.4 - 0.57	1.00	-0.02	-0.95 - 0.91	1.00	-0.27	-1.87 - 1.32	1.00	-0.03	-1.44 - 1.37	1.00	-0.05	-1.34 - 1.25	1.00	-0.75	-2.34 - 0.85	1.00	-0.20	-0.87 - 0.47	1.00	-0.57	-2.37 - 1.22	1.00
:McKesson	0.1	0.02	-1.23 - 1.27	1.00	-0.14	-1.11 - 0.83	1.00	-0.08	-1.9 - 1.74	1.00	-0.50	-1.85 - 0.85	1.00	-0.25	-1.66 - 1.16	1.00	0.20	-1.4 - 1.79	1.00	-1.04	-2.14 - 0.06	0.08	-0.64	-2.69 - 1.41	1.00
	0.5	-0.53	-1.32 - 0.25	0.66	-0.93**	-1.7 - -0.15	0.01	-0.63	-1.93 - 0.67	1.00	-1.32**	-2.39 - -0.25	0.01	-0.55	-1.51 - 0.42	1.00	-0.48	-1.68 - 0.72	1.00	-0.60	-1.21 - 0	0.05	-1.01	-2.52 - 0.49	0.70
	0.9	0.24	-0.76 - 1.23	1.00	-0.12	-0.99 - 0.76	1.00	-0.13	-1.67 - 1.42	1.00	0.38	-0.85 - 1.6	1.00	0.34	-0.87 - 1.55	1.00	0.11	-1.49 - 1.7	1.00	-0.19	-0.83 - 0.44	1.00	0.35	-1.21 - 1.92	1.00
CPOE for Laboratory Orders	0.1	-0.33	-0.83 - 0.18	0.82	-0.26	-0.6 - 0.08	0.36	-0.53	-1.21 - 0.16	0.36	-0.42	-1.02 - 0.18	0.57	-0.53	-1.09 - 0.03	0.08	-0.12	-0.7 - 0.46	1.00	-0.85***	-1.28 - -0.42	0.00	-0.56	-1.37 - 0.26	0.64
	0.5	-0.33*	-0.65 - -0.01	0.04	-0.50***	-0.81 - -0.18	0.00	-0.57*	-1.1 - -0.04	0.03	-0.66***	-1.1 - -0.23	0.00	-0.52**	-0.91 - -0.12	0.00	-0.58**	-1.07 - -0.1	0.01	-0.48***	-0.72 - -0.23	0.00	-0.95***	-1.56 - -0.33	0.00
	0.9	-0.12	-0.53 - 0.3	1.00	-0.36	-0.72 - 0	0.05	-0.17	-0.72 - 0.38	1.00	-0.14	-0.63 - 0.35	1.00	-0.31	-0.78 - 0.15	0.71	-0.15	-0.73 - 0.43	1.00	-0.12	-0.37 - 0.13	1.00	-0.65	-1.3 - 0	0.05
:MEDITECH	0.1	0.56	-0.21 - 1.33	0.49	0.34	-0.26 - 0.94	1.00	1.24*	0.11 - 2.37	0.02	0.92	-0.04 - 1.88	0.07	0.30	-0.57 - 1.17	1.00	-0.01	-0.93 - 0.91	1.00	0.77*	0.08 - 1.45	0.02	1.29	-0.08 - 2.66	0.09
	0.5	0.16	-0.37 - 0.68	1.00	0.26	-0.26 - 0.78	1.00	0.86	-0.01 - 1.74	0.06	0.54	-0.19 - 1.26	0.43	0.41	-0.24 - 1.06	0.95	0.74	-0.07 - 1.54	0.11	0.46*	0.05 - 0.86	0.02	0.50	-0.51 - 1.52	1.00
	0.9	0.31	-0.3 - 0.91	1.00	0.50	-0.05 - 1.05	0.11	0.21	-0.74 - 1.17	1.00	0.54	-0.3 - 1.37	0.85	-0.02	-0.77 - 0.73	1.00	0.10	-0.8 - 1	1.00	-0.04	-0.41 - 0.33	1.00	0.58	-0.41 - 1.57	1.00
:Epic	0.1	-0.53	-1.83 - 0.76	1.00	-1.00*	-1.99 - -0.02	0.04	-0.01	-1.48 - 1.46	1.00	-0.63	-1.95 - 0.69	1.00	0.18	-1.28 - 1.64	1.00	-1.15	-2.74 - 0.44	0.49	0.38	-0.54 - 1.29	1.00	0.23	-2.12 - 2.59	1.00

©2020 Murphy ZR et al. *JAMA Network Open*.

		0.5	-0.08	-0.88 - 0.72	1.00	-0.21	-0.99 - 0.58	1.00	0.25	-1.07 - 1.57	1.00	0.46	-0.62 - 1.55	1.00	0.27	-0.71 - 1.25	1.00	0.00	-1.21 - 1.22	1.00	0.00	-0.62 - 0.61	1.00	0.55	-0.97 - 2.08	1.00
		0.9	0.12	-1.05 - 1.29	1.00	0.32	-0.51 - 1.15	1.00	-0.59	-2.48 - 1.3	1.00	0.43	-0.8 - 1.65	1.00	0.66	-0.43 - 1.74	1.00	0.28	-0.84 - 1.39	1.00	-0.03	-0.59 - 0.53	1.00	-0.95	-2.48 - 0.59	1.00
	:Cerner	0.1	0.40	-0.33 - 1.13	1.00	0.11	-0.46 - 0.67	1.00	0.19	-0.75 - 1.13	1.00	0.05	-0.77 - 0.87	1.00	0.59	-0.25 - 1.42	0.57	0.10	-0.74 - 0.94	1.00	0.42	-0.17 - 1.01	0.55	-0.01	-1.2 - 1.17	1.00
		0.5	-0.07	-0.56 - 0.42	1.00	0.40	-0.09 - 0.88	0.24	0.05	-0.76 - 0.86	1.00	0.17	-0.5 - 0.84	1.00	0.06	-0.54 - 0.67	1.00	0.17	-0.58 - 0.92	1.00	0.26	-0.12 - 0.63	0.65	0.27	-0.66 - 1.21	1.00
		0.9	-0.38	-1.03 - 0.26	1.00	0.21	-0.38 - 0.8	1.00	-0.70	-1.62 - 0.22	0.38	-0.70	-1.51 - 0.12	0.17	0.05	-0.7 - 0.8	1.00	-0.30	-1.27 - 0.67	1.00	-0.07	-0.49 - 0.35	1.00	-0.14	-1.19 - 0.91	1.00
	:McKesson	0.1	0.16	-0.53 - 0.86	1.00	0.14	-0.44 - 0.72	1.00	0.00	-0.95 - 0.96	1.00	-0.07	-0.93 - 0.78	1.00	0.69	-0.17 - 1.55	0.27	0.16	-0.67 - 0.98	1.00	0.88***	0.28 - 1.47	0.00	0.67	-0.58 - 1.93	1.00
		0.5	-0.10	-0.6 - 0.4	1.00	0.38	-0.11 - 0.88	0.33	0.10	-0.73 - 0.92	1.00	0.13	-0.56 - 0.81	1.00	0.29	-0.33 - 0.9	1.00	0.35	-0.42 - 1.11	1.00	0.47**	0.08 - 0.85	0.01	0.30	-0.66 - 1.26	1.00
		0.9	-0.13	-0.69 - 0.43	1.00	0.04	-0.49 - 0.56	1.00	-0.10	-1.04 - 0.83	1.00	-0.12	-0.93 - 0.68	1.00	0.15	-0.56 - 0.86	1.00	-0.21	-1.14 - 0.72	1.00	0.20	-0.16 - 0.56	1.00	-0.21	-1.18 - 0.77	1.00
Patient Electronic Access (Available)		0.1	0.45	-0.03 - 0.93	0.08	0.25	-0.13 - 0.63	0.80	0.30	-0.39 - 0.99	1.00	-0.50	-1.07 - 0.07	0.15	0.10	-0.48 - 0.67	1.00	0.20	-0.41 - 0.8	1.00	-0.19	-0.61 - 0.23	1.00	0.28	-0.56 - 1.12	1.00
		0.5	0.01	-0.34 - 0.36	1.00	-0.12	-0.46 - 0.22	1.00	-0.18	-0.76 - 0.4	1.00	-0.16	-0.64 - 0.31	1.00	0.12	-0.31 - 0.55	1.00	-0.04	-0.57 - 0.5	1.00	0.16	-0.11 - 0.42	1.00	-0.08	-0.75 - 0.59	1.00
		0.9	-0.02	-0.43 - 0.38	1.00	-0.30	-0.69 - 0.09	0.33	0.00	-0.65 - 0.66	1.00	-0.07	-0.59 - 0.46	1.00	0.02	-0.47 - 0.51	1.00	-0.07	-0.72 - 0.59	1.00	0.01	-0.26 - 0.29	1.00	0.13	-0.62 - 0.87	1.00
	:MEDITECH	0.1	-0.21	-0.87 - 0.44	1.00	-0.10	-0.64 - 0.45	1.00	0.28	-0.71 - 1.26	1.00	0.71	-0.08 - 1.49	0.12	0.13	-0.7 - 0.95	1.00	0.09	-0.71 - 0.88	1.00	0.65*	0.07 - 1.23	0.02	0.72	-0.44 - 1.89	0.99
		0.5	0.22	-0.25 - 0.69	1.00	0.14	-0.32 - 0.61	1.00	0.23	-0.55 - 1.02	1.00	0.27	-0.38 - 0.91	1.00	0.30	-0.28 - 0.88	1.00	0.35	-0.36 - 1.07	1.00	-0.02	-0.38 - 0.34	1.00	0.27	-0.63 - 1.18	1.00
		0.9	-0.07	-0.59 - 0.45	1.00	0.28	-0.23 - 0.8	1.00	-0.44	-1.31 - 0.43	1.00	-0.30	-1.02 - 0.43	1.00	0.23	-0.43 - 0.89	1.00	0.09	-0.78 - 0.95	1.00	-0.03	-0.38 - 0.33	1.00	-0.37	-1.36 - 0.63	1.00
	:Epic	0.1	-0.25	-1.52 - 1.02	1.00	0.94	-0.29 - 2.16	0.36	-1.45	-3.84 - 0.94	1.00	0.16	-1.43 - 1.74	1.00	0.26	-1.29 - 1.8	1.00	0.15	-1.52 - 1.83	1.00	-0.20	-1.3 - 0.89	1.00	0.85	-1.32 - 3.02	1.00
		0.5	-0.52	-1.53 - 0.48	1.00	0.17	-0.82 - 1.17	1.00	-0.03	-1.69 - 1.64	1.00	0.28	-1.09 - 1.65	1.00	-0.44	-1.67 - 0.8	1.00	0.79	-0.74 - 2.33	1.00	-0.18	-0.95 - 0.6	1.00	0.20	-1.73 - 2.12	1.00
		0.9	0.37	-0.97 - 1.72	1.00	-0.12	-1.44 - 1.21	1.00	-1.08	-3.03 - 0.88	1.00	-1.00	-2.38 - 0.38	0.49	-0.48	-2.29 - 1.33	1.00	-0.60	-3.19 - 1.98	1.00	-0.38	-1.32 - 0.56	1.00	-0.56	-3.51 - 2.38	1.00
	:Cerner	0.1	-0.42	-1.12 - 0.29	1.00	-0.35	-0.94 - 0.23	1.00	-0.24	-1.24 - 0.75	1.00	0.04	-0.81 - 0.88	1.00	0.40	-0.4 - 1.2	1.00	-0.28	-1.16 - 0.6	1.00	0.33	-0.26 - 0.92	1.00	-0.24	-1.49 - 1.02	1.00
		0.5	-0.13	-0.63 - 0.36	1.00	0.06	-0.43 - 0.55	1.00	0.28	-0.54 - 1.1	1.00	-0.24	-0.91 - 0.44	1.00	0.09	-0.52 - 0.7	1.00	-0.09	-0.85 - 0.67	1.00	0.03	-0.35 - 0.41	1.00	-0.37	-1.32 - 0.58	1.00
		0.9	-0.06	-0.65 - 0.53	1.00	0.15	-0.4 - 0.71	1.00	0.31	-0.66 - 1.28	1.00	-0.11	-0.92 - 0.69	1.00	0.09	-0.64 - 0.83	1.00	-0.24	-1.14 - 0.66	1.00	0.20	-0.18 - 0.59	1.00	-0.57	-1.58 - 0.44	1.00
	:McKesson	0.1	-1.01**	-1.8 - -0.22	0.00	-0.79**	-1.38 - -0.21	0.00	-1.47**	-2.68 - -0.26	0.01	0.08	-0.82 - 0.97	1.00	-0.73	-1.66 - 0.2	0.31	-0.88	-1.91 - 0.15	0.18	0.24	-0.47 - 0.95	1.00	-1.99**	-3.45 - -0.52	0.00
		0.5	-0.30	-0.9 - 0.3	1.00	0.07	-0.52 - 0.66	1.00	-0.07	-1.07 - 0.92	1.00	0.38	-0.44 - 1.2	1.00	-0.02	-0.76 - 0.72	1.00	-0.48	-1.39 - 0.43	1.00	-0.09	-0.55 - 0.37	1.00	-0.42	-1.56 - 0.73	1.00
		0.9	0.33	-0.4 - 1.05	1.00	0.21	-0.53 - 0.95	1.00	0.57	-0.59 - 1.73	1.00	0.53	-0.51 - 1.56	1.00	-0.17	-1.09 - 0.75	1.00	0.41	-0.91 - 1.74	1.00	0.11	-0.37 - 0.6	1.00	-0.01	-1.34 - 1.32	1.00
Patient Electronic Access (Accessed)		0.1	-0.22	-0.86 - 0.42	1.00	0.39	-0.17 - 0.94	0.60	-0.51	-1.48 - 0.46	1.00	-0.10	-0.9 - 0.69	1.00	0.24	-0.53 - 1.02	1.00	0.02	-0.79 - 0.82	1.00	0.38	-0.15 - 0.9	0.51	0.12	-1 - 1.23	1.00
		0.5	-0.15	-0.53 - 0.22	1.00	-0.21	-0.57 - 0.16	1.00	0.06	-0.56 - 0.68	1.00	-0.20	-0.7 - 0.31	1.00	0.07	-0.38 - 0.53	1.00	-0.23	-0.8 - 0.34	1.00	-0.06	-0.35 - 0.22	1.00	-0.18	-0.9 - 0.53	1.00
		0.9	-0.07	-0.42 - 0.27	1.00	0.18	-0.29 - 0.64	1.00	-0.30	-0.86 - 0.26	1.00	0.19	-0.32 - 0.7	1.00	-0.06	-0.73 - 0.62	1.00	-0.33	-1.18 - 0.53	1.00	-0.02	-0.2 - 0.16	1.00	0.05	-0.59 - 0.69	1.00
	:MEDITECH	0.1	0.21	-1.26 - 1.67	1.00	-0.71	-2.01 - 0.59	1.00	0.49	-1 - 1.98	1.00	0.13	-1.05 - 1.3	1.00	-0.51	-2.27 - 1.25	1.00	0.13	-1.71 - 1.97	1.00	-0.33	-1.1 - 0.43	1.00	-0.55	-2.25 - 1.15	1.00
		0.5	0.18	-0.49 - 0.86	1.00	-0.15	-0.82 - 0.52	1.00	0.06	-1.06 - 1.18	1.00	-0.12	-1.04 - 0.81	1.00	-0.43	-1.27 - 0.4	1.00	-0.12	-1.15 - 0.92	1.00	-0.26	-0.78 - 0.26	1.00	-0.55	-1.85 - 0.75	1.00
		0.9	0.56	-0.01 - 1.12	0.06	-0.64	-1.29 - 0.02	0.07	0.50	-0.46 - 1.46	1.00	-0.14	-0.99 - 0.71	1.00	-0.92*	-1.81 - -0.02	0.04	0.30	-0.8 - 1.41	1.00	-0.08	-0.84 - 0.68	1.00	-0.56	-1.67 - 0.55	1.00

©2020 Murphy ZR et al. *JAMA Network Open*.

:Epic	0.1	0.71	-0.09 - 1.51	0.13	-0.07	-0.76 - 0.63	1.00	1.07	-0.03 - 2.18	0.07	0.66	-0.38 - 1.71	0.89	-0.10	-1.05 - 0.86	1.00	0.17	-0.76 - 1.1	1.00	0.03	-0.69 - 0.74	1.00	1.03	-0.47 - 2.53	0.64
	0.5	0.44	-0.05 - 0.93	0.13	0.64**	0.15 - 1.12	0.00	0.39	-0.43 - 1.2	1.00	0.99***	0.32 - 1.65	0.00	0.26	-0.34 - 0.87	1.00	0.64	-0.11 - 1.39	0.18	0.15	-0.22 - 0.53	1.00	1.08*	0.14 - 2.02	0.01
	0.9	0.15	-0.35 - 0.65	1.00	0.08	-0.49 - 0.65	1.00	1.34***	0.55 - 2.12	0.00	0.80**	0.12 - 1.49	0.01	0.11	-0.7 - 0.91	1.00	0.62	-0.36 - 1.6	0.91	0.26	-0.06 - 0.58	0.25	1.08*	0.13 - 2.03	0.01
:Cerner	0.1	0.74	-0.24 - 1.72	0.39	0.17	-0.65 - 0.98	1.00	0.65	-0.82 - 2.12	1.00	0.20	-0.89 - 1.28	1.00	0.61	-0.45 - 1.67	1.00	-0.19	-1.3 - 0.93	1.00	-0.02	-0.66 - 0.62	1.00	-0.23	-1.74 - 1.28	1.00
	0.5	0.58*	0.03 - 1.13	0.03	0.51	-0.04 - 1.06	0.09	0.05	-0.86 - 0.97	1.00	0.76	0 - 1.51	0.05	0.67	-0.01 - 1.35	0.06	0.55	-0.3 - 1.4	0.81	0.30	-0.13 - 0.72	0.59	0.86	-0.2 - 1.92	0.25
	0.9	0.91***	0.34 - 1.48	0.00	0.29	-0.34 - 0.91	1.00	0.90	-0.09 - 1.9	0.12	1.17**	0.21 - 2.12	0.01	0.46	-0.46 - 1.38	1.00	1.22*	0.15 - 2.29	0.01	0.18	-0.21 - 0.58	1.00	1.14	-0.08 - 2.36	0.09
:McKesson	0.1	-0.33	-1.78 - 1.12	1.00	-0.49	-1.66 - 0.68	1.00	1.00	-1.19 - 3.18	1.00	0.17	-1.6 - 1.93	1.00	0.09	-1.25 - 1.42	1.00	0.55	-1.26 - 2.35	1.00	-0.29	-1.15 - 0.58	1.00	-0.19	-2.69 - 2.3	1.00
	0.5	0.35	-0.42 - 1.11	1.00	0.61	-0.14 - 1.37	0.26	0.52	-0.74 - 1.79	1.00	0.63	-0.41 - 1.67	1.00	-0.29	-1.23 - 0.65	1.00	0.77	-0.4 - 1.93	0.77	0.03	-0.55 - 0.62	1.00	0.86	-0.61 - 2.32	1.00
	0.9	0.17	-0.55 - 0.89	1.00	-0.09	-0.92 - 0.74	1.00	0.79	-0.32 - 1.89	0.54	0.20	-0.82 - 1.22	1.00	0.18	-0.73 - 1.09	1.00	0.35	-0.85 - 1.56	1.00	0.08	-0.69 - 0.85	1.00	0.04	-2.05 - 2.12	1.00
Patient-Specific Education Resources	0.1	-0.08	-0.31 - 0.16	1.00	0.04	-0.16 - 0.25	1.00	-0.08	-0.4 - 0.25	1.00	0.07	-0.23 - 0.37	1.00	0.07	-0.22 - 0.36	1.00	-0.06	-0.37 - 0.25	1.00	0.09	-0.1 - 0.29	1.00	-0.23	-0.66 - 0.19	1.00
	0.5	0.01	-0.17 - 0.18	1.00	0.08	-0.09 - 0.25	1.00	0.21	-0.08 - 0.5	0.46	0.19	-0.05 - 0.42	0.32	0.18	-0.04 - 0.39	0.24	0.24	-0.03 - 0.5	0.13	0.02	-0.12 - 0.15	1.00	0.29	-0.04 - 0.63	0.15
	0.9	0.03	-0.18 - 0.23	1.00	0.20*	0.02 - 0.38	0.02	0.29	-0.02 - 0.6	0.10	0.16	-0.12 - 0.44	1.00	0.18	-0.08 - 0.44	0.57	0.24	-0.09 - 0.57	0.44	0.05	-0.09 - 0.19	1.00	0.21	-0.15 - 0.56	1.00
:MEDITECH	0.1	0.08	-0.29 - 0.46	1.00	-0.08	-0.39 - 0.24	1.00	0.44	-0.06 - 0.93	0.14	-0.03	-0.46 - 0.41	1.00	-0.21	-0.64 - 0.23	1.00	0.54*	0.06 - 1.03	0.01	-0.14	-0.49 - 0.2	1.00	0.27	-0.43 - 0.96	1.00
	0.5	-0.01	-0.28 - 0.26	1.00	0.06	-0.2 - 0.33	1.00	-0.32	-0.77 - 0.12	0.47	-0.09	-0.45 - 0.28	1.00	-0.11	-0.44 - 0.23	1.00	-0.27	-0.68 - 0.14	0.75	-0.03	-0.24 - 0.18	1.00	-0.01	-0.52 - 0.51	1.00
	0.9	0.06	-0.28 - 0.39	1.00	-0.01	-0.33 - 0.31	1.00	-0.47	-0.97 - 0.03	0.09	0.02	-0.43 - 0.48	1.00	-0.21	-0.6 - 0.19	1.00	-0.18	-0.7 - 0.34	1.00	-0.04	-0.27 - 0.19	1.00	0.02	-0.52 - 0.57	1.00
:Epic	0.1	0.30	-0.3 - 0.9	1.00	-0.09	-0.63 - 0.46	1.00	0.51	-0.41 - 1.43	1.00	0.10	-0.58 - 0.78	1.00	-0.07	-0.84 - 0.69	1.00	-0.08	-0.98 - 0.82	1.00	0.07	-0.36 - 0.49	1.00	-0.20	-1.24 - 0.84	1.00
	0.5	0.13	-0.28 - 0.54	1.00	0.05	-0.36 - 0.46	1.00	-0.23	-0.92 - 0.45	1.00	-0.07	-0.63 - 0.49	1.00	0.21	-0.3 - 0.72	1.00	0.04	-0.59 - 0.67	1.00	0.00	-0.31 - 0.32	1.00	-0.23	-1.02 - 0.56	1.00
	0.9	0.07	-0.44 - 0.59	1.00	-0.06	-0.56 - 0.44	1.00	0.18	-0.67 - 1.04	1.00	0.05	-0.67 - 0.76	1.00	0.04	-0.54 - 0.62	1.00	0.45	-0.32 - 1.22	1.00	0.06	-0.32 - 0.44	1.00	0.50	-0.51 - 1.51	1.00
:Cerner	0.1	-0.02	-0.46 - 0.42	1.00	-0.11	-0.46 - 0.25	1.00	-0.16	-0.77 - 0.45	1.00	-0.23	-0.71 - 0.24	1.00	-0.02	-0.49 - 0.44	1.00	0.21	-0.29 - 0.71	1.00	-0.12	-0.45 - 0.2	1.00	-0.06	-0.73 - 0.61	1.00
	0.5	-0.18	-0.47 - 0.11	1.00	-0.37**	-0.66 - -0.08	0.00	-0.60**	-1.09 - -0.11	0.01	-0.19	-0.59 - 0.22	1.00	-0.33	-0.69 - 0.04	0.12	-0.10	-0.55 - 0.35	1.00	-0.10	-0.33 - 0.12	1.00	-0.45	-1.01 - 0.12	0.29
	0.9	0.00	-0.36 - 0.36	1.00	-0.36*	-0.69 - -0.03	0.02	-0.79***	-1.35 - -0.23	0.00	-0.30	-0.78 - 0.18	0.95	-0.02	-0.45 - 0.41	1.00	-0.43	-0.99 - 0.12	0.32	-0.08	-0.31 - 0.16	1.00	-0.56	-1.19 - 0.07	0.13
:McKesson	0.1	0.18	-0.21 - 0.56	1.00	-0.32*	-0.64 - -0.01	0.03	0.27	-0.28 - 0.82	1.00	-0.05	-0.52 - 0.42	1.00	-0.29	-0.77 - 0.2	1.00	0.12	-0.41 - 0.64	1.00	0.05	-0.27 - 0.37	1.00	0.09	-0.63 - 0.82	1.00
	0.5	0.05	-0.24 - 0.33	1.00	-0.09	-0.37 - 0.19	1.00	0.19	-0.28 - 0.65	1.00	-0.01	-0.39 - 0.38	1.00	-0.18	-0.53 - 0.16	1.00	-0.03	-0.46 - 0.4	1.00	-0.04	-0.26 - 0.18	1.00	-0.33	-0.87 - 0.22	1.00
	0.9	-0.25	-0.59 - 0.08	0.36	-0.36*	-0.69 - -0.03	0.02	-0.15	-0.66 - 0.35	1.00	-0.42	-0.87 - 0.03	0.08	-0.22	-0.66 - 0.22	1.00	-0.22	-0.73 - 0.29	1.00	-0.01	-0.24 - 0.22	1.00	-0.40	-0.93 - 0.12	0.36
Medication Reconciliation	0.1	0.60***	0.18 - 1.02	0.00	0.36	0 - 0.73	0.05	0.58	-0.07 - 1.24	0.14	-0.23	-0.83 - 0.36	1.00	0.38	-0.16 - 0.91	0.56	-0.01	-0.59 - 0.57	1.00	-0.03	-0.44 - 0.38	1.00	0.04	-0.76 - 0.84	1.00
	0.5	0.33	-0.01 - 0.67	0.07	0.23	-0.11 - 0.57	0.67	0.77**	0.20 - 1.34	0.00	-0.18	-0.65 - 0.29	1.00	0.38	-0.04 - 0.8	0.13	0.37	-0.15 - 0.9	0.53	0.28*	0.01 - 0.54	0.03	0.06	-0.6 - 0.72	1.00
	0.9	0.45*	0.03 - 0.86	0.03	0.00	-0.37 - 0.36	1.00	0.84**	0.21 - 1.47	0.00	0.17	-0.38 - 0.71	1.00	0.42	-0.13 - 0.98	0.37	0.44	-0.22 - 1.1	0.72	-0.05	-0.31 - 0.22	1.00	0.40	-0.31 - 1.11	1.00
:MEDITECH	0.1	-0.35	-0.99 - 0.29	1.00	-0.18	-0.72 - 0.37	1.00	-0.44	-1.31 - 0.43	1.00	0.73	-0.09 - 1.56	0.14	-0.10	-0.88 - 0.68	1.00	-0.18	-1.02 - 0.66	1.00	0.20	-0.36 - 0.76	1.00	0.14	-1.01 - 1.29	1.00
	0.5	-0.08	-0.55 - 0.4	1.00	0.03	-0.44 - 0.5	1.00	-0.36	-1.15 - 0.43	1.00	0.34	-0.31 - 1	1.00	-0.32	-0.91 - 0.27	1.00	-0.33	-1.06 - 0.4	1.00	-0.14	-0.51 - 0.22	1.00	-0.03	-0.94 - 0.89	1.00

©2020 Murphy ZR et al. *JAMA Network Open*.

		0.9	-0.15	-0.7 - 0.4	1.00	0.09	-0.43 - 0.62	1.00	-0.37	-1.29 - 0.56	1.00	0.46	-0.34 - 1.26	1.00	-0.36	-1.09 - 0.37	1.00	-0.32	-1.25 - 0.61	1.00	-0.04	-0.43 - 0.36	1.00	-0.19	-1.17 - 0.78	1.00
	:Epic	0.1	-1.19**	-2.16 - -0.22	0.01	-0.41	-1.27 - 0.44	1.00	-0.80	-2.15 - 0.56	1.00	-0.15	-1.27 - 0.97	1.00	-0.81	-1.9 - 0.29	0.44	-0.16	-1.42 - 1.09	1.00	-0.02	-0.9 - 0.85	1.00	0.73	-0.94 - 2.4	1.00
		0.5	-0.60	-1.35 - 0.15	0.27	-0.23	-0.97 - 0.51	1.00	-1.37*	-2.61 - -0.13	0.02	0.27	-0.75 - 1.29	1.00	-0.61	-1.53 - 0.31	0.73	-0.99	-2.13 - 0.15	0.16	-0.43	-1 - 0.15	0.43	-0.29	-1.72 - 1.14	1.00
		0.9	-0.59	-1.61 - 0.42	1.00	0.21	-0.64 - 1.06	1.00	-1.09	-2.81 - 0.62	0.89	-0.54	-1.84 - 0.75	1.00	-0.78	-2.01 - 0.46	0.93	-0.97	-2.37 - 0.43	0.60	-0.17	-0.67 - 0.34	1.00	-0.95	-2.83 - 0.92	1.00
	:Cerner	0.1	-0.41	-1.26 - 0.44	1.00	0.01	-0.7 - 0.72	1.00	0.00	-1.08 - 1.07	1.00	1.36***	0.46 - 2.26	0.00	-0.18	-1.19 - 0.82	1.00	-0.05	-1.02 - 0.93	1.00	0.81*	0.05 - 1.56	0.03	0.29	-1.03 - 1.61	1.00
		0.5	0.01	-0.59 - 0.6	1.00	-0.29	-0.88 - 0.3	1.00	-0.11	-1.09 - 0.88	1.00	-0.61	-1.42 - 0.21	0.41	-0.22	-0.96 - 0.51	1.00	-0.24	-1.15 - 0.67	1.00	-0.01	-0.47 - 0.45	1.00	0.25	-0.89 - 1.4	1.00
		0.9	-0.11	-0.88 - 0.66	1.00	-0.04	-0.71 - 0.63	1.00	-0.47	-1.68 - 0.74	1.00	-0.46	-1.46 - 0.54	1.00	0.17	-0.76 - 1.1	1.00	-0.49	-1.65 - 0.67	1.00	0.18	-0.31 - 0.67	1.00	-0.09	-1.41 - 1.23	1.00
	:McKesson	0.1	0.84	-0.12 - 1.79	0.15	0.70	-0.05 - 1.46	0.09	1.32	-0.01 - 2.65	0.05	1.48**	0.29 - 2.67	0.00	0.77	-0.34 - 1.89	0.60	-0.16	-1.27 - 0.96	1.00	0.65	-0.08 - 1.38	0.14	2.77***	1.12 - 4.43	0.00
		0.5	0.17	-0.48 - 0.82	1.00	-0.07	-0.71 - 0.57	1.00	-0.39	-1.46 - 0.68	1.00	0.63	-0.25 - 1.52	0.52	-0.03	-0.83 - 0.77	1.00	-0.22	-1.21 - 0.77	1.00	0.15	-0.35 - 0.65	1.00	0.76	-0.49 - 2	1.00
		0.9	0.02	-0.79 - 0.82	1.00	-0.04	-0.74 - 0.65	1.00	-0.70	-1.93 - 0.54	1.00	-0.31	-1.38 - 0.77	1.00	-0.93	-1.93 - 0.07	0.09	-0.56	-1.78 - 0.67	1.00	0.10	-0.38 - 0.58	1.00	0.09	-1.22 - 1.39	1.00
Electronic Health Information Exchange		0.1	0.10	-0.22 - 0.41	1.00	-0.06	-0.27 - 0.14	1.00	0.18	-0.18 - 0.55	1.00	0.16	-0.19 - 0.5	1.00	-0.03	-0.34 - 0.28	1.00	-0.23	-0.57 - 0.12	0.75	0.15	-0.1 - 0.39	1.00	-0.13	-0.64 - 0.39	1.00
		0.5	0.10	-0.1 - 0.3	1.00	0.05	-0.15 - 0.25	1.00	0.19	-0.14 - 0.52	1.00	-0.06	-0.34 - 0.21	1.00	-0.02	-0.27 - 0.22	1.00	-0.01	-0.32 - 0.3	1.00	-0.03	-0.19 - 0.12	1.00	0.07	-0.32 - 0.46	1.00
		0.9	0.24*	0.03 - 0.46	0.02	0.02	-0.18 - 0.22	1.00	0.56***	0.25 - 0.88	0.00	0.04	-0.26 - 0.33	1.00	0.03	-0.24 - 0.3	1.00	0.30	-0.05 - 0.65	0.17	-0.12	-0.27 - 0.03	0.34	0.21	-0.17 - 0.59	1.00
	:MEDITECH	0.1	0.08	-0.35 - 0.51	1.00	0.04	-0.25 - 0.34	1.00	0.05	-0.49 - 0.58	1.00	-0.03	-0.52 - 0.45	1.00	0.14	-0.32 - 0.61	1.00	0.22	-0.27 - 0.71	1.00	-0.13	-0.47 - 0.21	1.00	0.45	-0.24 - 1.15	0.77
		0.5	-0.06	-0.34 - 0.21	1.00	0.03	-0.24 - 0.31	1.00	-0.02	-0.48 - 0.43	1.00	0.22	-0.16 - 0.6	1.00	-0.13	-0.47 - 0.21	1.00	0.16	-0.26 - 0.58	1.00	0.13	-0.09 - 0.34	1.00	0.16	-0.37 - 0.69	1.00
		0.9	-0.10	-0.4 - 0.2	1.00	0.11	-0.17 - 0.39	1.00	-0.38	-0.85 - 0.09	0.26	0.08	-0.34 - 0.5	1.00	0.09	-0.3 - 0.47	1.00	0.00	-0.51 - 0.5	1.00	0.05	-0.17 - 0.26	1.00	0.10	-0.44 - 0.64	1.00
	:Epic	0.1	-0.02	-0.42 - 0.38	1.00	0.02	-0.29 - 0.33	1.00	0.10	-0.41 - 0.61	1.00	0.25	-0.22 - 0.71	1.00	0.26	-0.17 - 0.69	1.00	0.36	-0.12 - 0.84	0.39	0.06	-0.29 - 0.4	1.00	0.43	-0.27 - 1.14	1.00
		0.5	0.04	-0.24 - 0.32	1.00	-0.01	-0.29 - 0.27	1.00	0.08	-0.39 - 0.55	1.00	0.18	-0.21 - 0.56	1.00	0.08	-0.27 - 0.43	1.00	0.12	-0.31 - 0.55	1.00	0.14	-0.08 - 0.36	0.91	0.04	-0.5 - 0.59	1.00
		0.9	-0.12	-0.43 - 0.19	1.00	0.07	-0.22 - 0.36	1.00	-0.44	-0.93 - 0.05	0.12	0.10	-0.33 - 0.53	1.00	-0.03	-0.42 - 0.35	1.00	-0.23	-0.74 - 0.28	1.00	0.16	-0.06 - 0.37	0.44	-0.18	-0.76 - 0.39	1.00
	:Cerner	0.1	-0.03	-0.46 - 0.39	1.00	-0.02	-0.34 - 0.31	1.00	-0.26	-0.79 - 0.27	1.00	0.19	-0.28 - 0.65	1.00	-0.24	-0.69 - 0.22	1.00	0.27	-0.22 - 0.76	1.00	0.08	-0.26 - 0.41	1.00	0.47	-0.25 - 1.19	0.82
		0.5	-0.11	-0.39 - 0.17	1.00	0.07	-0.21 - 0.34	1.00	-0.21	-0.67 - 0.26	1.00	0.17	-0.22 - 0.55	1.00	-0.08	-0.42 - 0.27	1.00	-0.18	-0.61 - 0.25	1.00	0.21	0 - 0.43	0.06	0.17	-0.37 - 0.7	1.00
		0.9	-0.10	-0.41 - 0.21	1.00	0.14	-0.14 - 0.41	1.00	-0.45	-0.93 - 0.03	0.09	-0.04	-0.45 - 0.38	1.00	-0.10	-0.47 - 0.27	1.00	-0.56**	-1.04 - -0.08	0.01	0.26**	0.05 - 0.47	0.00	-0.03	-0.56 - 0.51	1.00
	:McKesson	0.1	-0.01	-0.58 - 0.56	1.00	0.43*	0.04 - 0.82	0.02	-0.12	-0.85 - 0.61	1.00	0.56	-0.02 - 1.15	0.07	0.05	-0.59 - 0.7	1.00	0.12	-0.54 - 0.77	1.00	0.20	-0.25 - 0.64	1.00	0.40	-0.5 - 1.31	1.00
		0.5	-0.03	-0.38 - 0.32	1.00	-0.07	-0.42 - 0.27	1.00	0.03	-0.56 - 0.61	1.00	0.10	-0.38 - 0.58	1.00	0.04	-0.39 - 0.47	1.00	0.09	-0.45 - 0.63	1.00	0.11	-0.16 - 0.38	1.00	0.23	-0.44 - 0.91	1.00
		0.9	-0.46**	-0.81 - -0.1	0.00	0.02	-0.32 - 0.37	1.00	-0.23	-0.81 - 0.34	1.00	0.12	-0.41 - 0.64	1.00	0.34	-0.15 - 0.83	0.64	-0.50	-1.19 - 0.19	0.49	0.11	-0.19 - 0.41	1.00	0.31	-0.33 - 0.94	1.00
MEDITECH		0.1	1.87*	0.17 - 3.56	0.02	-0.30	-1.83 - 1.24	1.00	0.85	-1.53 - 3.22	1.00	0.73	-1.32 - 2.78	1.00	-0.34	-2.47 - 1.79	1.00	0.01	-2.02 - 2.04	1.00	1.57*	0.12 - 3.02	0.02	1.65	-1.31 - 4.62	1.00
		0.5	-0.37	-1.52 - 0.78	1.00	-0.87	-2.01 - 0.27	0.36	-0.69	-2.6 - 1.22	1.00	-0.26	-1.83 - 1.31	1.00	-0.18	-1.6 - 1.23	1.00	-1.32	-3.07 - 0.44	0.40	0.04	-0.85 - 0.93	1.00	-0.64	-2.85 - 1.57	1.00
		0.9	0.31	-1.03 - 1.65	1.00	-1.51**	-2.78 - -0.24	0.01	-0.88	-3.02 - 1.26	1.00	-1.14	-2.93 - 0.66	0.90	-2.06**	-3.73 - -0.38	0.01	-1.09	-3.17 - 0.99	1.00	0.50	-0.45 - 1.44	1.00	-0.19	-2.45 - 2.06	1.00

©2020 Murphy ZR et al. *JAMA Network Open*.

	:Num Beds 100-199	0.1	-1.81	-3.78 - 0.16	0.10	0.43	-1.17 - 2.03	1.00	-0.21	-2.9 - 2.48	1.00	-0.70	-3.07 - 1.68	1.00	1.85	-0.51 - 4.21	0.31	-0.70	-2.98 - 1.58	1.00	-0.35	-2.02 - 1.31	1.00	-1.06	-4.5 - 2.39	1.00
		0.5	1.40	-0.01 - 2.8	0.05	1.02	-0.37 - 2.41	0.46	1.14	-1.19 - 3.47	1.00	-0.05	-1.97 - 1.87	1.00	1.25	-0.48 - 2.99	0.49	0.80	-1.35 - 2.94	1.00	0.75	-0.33 - 1.84	0.60	1.60	-1.1 - 4.3	1.00
		0.9	1.04	-0.66 - 2.75	1.00	1.38	-0.09 - 2.85	0.09	1.88	-0.72 - 4.47	0.49	0.21	-2.02 - 2.44	1.00	1.33	-0.67 - 3.33	0.73	0.27	-2.26 - 2.81	1.00	0.31	-0.76 - 1.38	1.00	-1.08	-3.86 - 1.7	1.00
	:Num Beds 200-299	0.1	-0.63	-3.3 - 2.03	1.00	0.29	-1.91 - 2.49	1.00	0.43	-3.11 - 3.97	1.00	-1.39	-4.6 - 1.83	1.00	1.71	-1.32 - 4.74	1.00	-1.07	-4.07 - 1.94	1.00	-0.11	-2.33 - 2.1	1.00	0.68	-3.6 - 4.96	1.00
		0.5	0.75	-1.08 - 2.58	1.00	0.16	-1.65 - 1.97	1.00	-0.55	-3.58 - 2.48	1.00	-0.81	-3.31 - 1.68	1.00	0.26	-1.99 - 2.51	1.00	-0.62	-3.41 - 2.18	1.00	0.18	-1.23 - 1.59	1.00	-0.08	-3.59 - 3.43	1.00
		0.9	-0.12	-2.09 - 1.84	1.00	0.49	-1.41 - 2.39	1.00	0.55	-2.55 - 3.65	1.00	-0.31	-3.05 - 2.43	1.00	2.31	-0.4 - 5.02	0.18	-1.52	-4.64 - 1.61	1.00	-0.79	-2.2 - 0.62	1.00	-3.17	-6.5 - 0.15	0.08
	:Num Beds 300-399	0.1	-1.08	-4.63 - 2.47	1.00	2.75	-0.3 - 5.81	0.12	2.92	-2.05 - 7.9	1.00	2.55	-1.56 - 6.66	0.99	1.53	-2.75 - 5.81	1.00	0.72	-4.02 - 5.45	1.00	0.15	-2.68 - 2.98	1.00	-1.99	-9.09 - 5.12	1.00
		0.5	1.70	-0.69 - 4.08	0.54	1.68	-0.68 - 4.05	0.53	0.17	-3.78 - 4.12	1.00	0.02	-3.24 - 3.28	1.00	1.30	-1.64 - 4.24	1.00	0.69	-2.95 - 4.33	1.00	0.31	-1.53 - 2.15	1.00	0.98	-3.6 - 5.56	1.00
		0.9	2.45	-0.24 - 5.15	0.11	2.09	-0.44 - 4.61	0.22	2.18	-2.06 - 6.42	1.00	0.00	-3.58 - 3.59	1.00	4.42***	1.26 - 7.58	0.00	1.16	-3.11 - 5.43	1.00	-0.46	-2.11 - 1.2	1.00	-0.27	-4.55 - 4.01	1.00
	:Num Beds 400-499	0.1	-1.56	-7.72 - 4.61	1.00	1.23	-3.84 - 6.3	1.00	3.94	-3.47 - 11.36	1.00	1.35	-5.2 - 7.91	1.00	1.49	-4.96 - 7.93	1.00	1.71	-5.38 - 8.8	1.00	-1.00	-5.19 - 3.19	1.00	-2.93	-12.67 - 6.82	1.00
		0.5	0.96	-2.85 - 4.76	1.00	0.86	-2.91 - 4.63	1.00	3.08	-3.24 - 9.39	1.00	-1.58	-6.78 - 3.62	1.00	0.80	-3.89 - 5.49	1.00	2.19	-3.63 - 8	1.00	0.71	-2.23 - 3.64	1.00	-1.27	-8.57 - 6.04	1.00
		0.9	0.67	-3.1 - 4.44	1.00	-0.43	-5.94 - 5.07	1.00	0.53	-6.59 - 7.65	1.00	0.83	-4.99 - 6.65	1.00	4.51	-1.75 - 10.77	0.50	-0.71	-10.61 - 9.19	1.00	-0.87	-4.36 - 2.62	1.00	-4.42	-12.57 - 3.74	1.00
	:Num Beds 500+	0.1	-5.60*	-10.84 - -0.36	0.03	-4.50*	-8.74 - -0.25	0.03	-2.95	-10.86 - 4.96	1.00	-3.62	-10.32 - 3.09	1.00	-2.74	-8.88 - 3.4	1.00	-3.04	-9.38 - 3.3	1.00	-1.86	-6.49 - 2.76	1.00	-10.26*	-19.47 - -1.06	0.02
		0.5	1.36	-2.12 - 4.83	1.00	2.19	-1.24 - 5.63	0.88	1.04	-4.71 - 6.79	1.00	0.54	-4.2 - 5.28	1.00	-0.63	-4.91 - 3.65	1.00	0.38	-4.92 - 5.68	1.00	0.45	-2.23 - 3.12	1.00	0.55	-6.11 - 7.21	1.00
		0.9	0.64	-3.64 - 4.92	1.00	2.19	-1.68 - 6.05	1.00	0.25	-6.51 - 7.01	1.00	-0.04	-5.77 - 5.69	1.00	2.31	-3.15 - 7.78	1.00	-2.93	-9.78 - 3.92	1.00	0.25	-2.47 - 2.98	1.00	-3.29	-10.89 - 4.3	1.00
Epic		0.1	1.43	-0.83 - 3.69	0.92	-0.78	-3.06 - 1.49	1.00	2.67	-0.97 - 6.31	0.46	0.60	-1.99 - 3.19	1.00	0.04	-2.76 - 2.83	1.00	1.94	-1.44 - 5.32	1.00	0.35	-1.71 - 2.41	1.00	-0.82	-5.02 - 3.38	1.00
		0.5	0.38	-1.35 - 2.1	1.00	-0.97	-2.68 - 0.74	1.00	0.07	-2.79 - 2.93	1.00	-0.51	-2.86 - 1.85	1.00	-0.48	-2.6 - 1.65	1.00	-1.11	-3.74 - 1.53	1.00	0.16	-1.17 - 1.49	1.00	0.40	-2.92 - 3.71	1.00
		0.9	0.74	-1.61 - 3.09	1.00	-1.61	-3.94 - 0.73	0.62	0.38	-3.56 - 4.32	1.00	-0.45	-3.41 - 2.52	1.00	0.31	-2.77 - 3.4	1.00	-2.40	-6.3 - 1.49	1.00	0.12	-1.31 - 1.56	1.00	0.13	-4.74 - 5	1.00
	:Num Beds 100-199	0.1	-0.24	-2.39 - 1.9	1.00	1.82*	0.01 - 3.64	0.05	-0.19	-3.23 - 2.84	1.00	0.36	-2.31 - 3.03	1.00	1.70	-0.93 - 4.32	0.84	-1.97	-4.61 - 0.67	0.42	0.32	-1.5 - 2.15	1.00	2.92	-0.91 - 6.75	0.37
		0.5	0.17	-1.4 - 1.73	1.00	0.58	-0.97 - 2.13	1.00	-0.68	-3.28 - 1.92	1.00	-0.74	-2.88 - 1.4	1.00	-0.44	-2.37 - 1.49	1.00	-0.46	-2.85 - 1.93	1.00	0.45	-0.76 - 1.65	1.00	-0.50	-3.5 - 2.51	1.00
		0.9	-0.50	-2.33 - 1.32	1.00	0.03	-1.67 - 1.72	1.00	-1.15	-4.05 - 1.75	1.00	-0.30	-2.75 - 2.16	1.00	-1.55	-3.72 - 0.63	0.54	0.51	-2.4 - 3.42	1.00	0.97	-0.17 - 2.11	0.18	-1.11	-4.21 - 1.99	1.00
	:Num Beds 200-299	0.1	-0.73	-3.43 - 1.97	1.00	0.99	-1.31 - 3.28	1.00	-1.98	-5.78 - 1.81	1.00	-1.55	-4.73 - 1.62	1.00	-0.18	-3.21 - 2.85	1.00	-2.77	-6.19 - 0.66	0.26	0.25	-1.95 - 2.45	1.00	1.15	-3.35 - 5.64	1.00
		0.5	-0.99	-2.88 - 0.9	1.00	0.86	-1.01 - 2.73	1.00	-1.54	-4.68 - 1.59	1.00	-1.79	-4.37 - 0.8	0.61	-0.29	-2.62 - 2.04	1.00	-1.11	-4 - 1.78	1.00	0.26	-1.2 - 1.72	1.00	-0.26	-3.89 - 3.37	1.00
		0.9	-1.46	-3.5 - 0.58	0.51	0.40	-1.63 - 2.42	1.00	-2.02	-5.37 - 1.33	1.00	0.32	-2.4 - 3.03	1.00	0.80	-1.83 - 3.43	1.00	-0.24	-3.67 - 3.2	1.00	-0.01	-1.38 - 1.37	1.00	-1.71	-5.28 - 1.86	1.00
	:Num Beds 300-399	0.1	-2.00	-5.24 - 1.23	0.99	1.29	-1.47 - 4.05	1.00	-2.02	-6.72 - 2.67	1.00	1.69	-2.1 - 5.49	1.00	-1.02	-4.93 - 2.89	1.00	-3.39	-7.51 - 0.72	0.23	-0.86	-3.48 - 1.75	1.00	-1.41	-7.8 - 4.98	1.00
		0.5	0.14	-2.16 - 2.44	1.00	0.05	-2.23 - 2.32	1.00	-1.30	-5.11 - 2.51	1.00	-0.55	-3.69 - 2.59	1.00	-0.96	-3.79 - 1.87	1.00	-0.26	-3.77 - 3.25	1.00	-0.15	-1.92 - 1.62	1.00	-1.25	-5.66 - 3.16	1.00
		0.9	-0.70	-3.34 - 1.94	1.00	0.22	-2.27 - 2.71	1.00	-1.08	-5.46 - 3.3	1.00	-1.60	-5.26 - 2.05	1.00	0.38	-2.76 - 3.52	1.00	0.83	-3.34 - 5	1.00	0.30	-1.36 - 1.97	1.00	-1.40	-5.5 - 2.7	1.00
	:Num Beds 400-499	0.1	-0.70	-5.94 - 4.54	1.00	-0.34	-4.47 - 3.78	1.00	2.20	-3.49 - 7.89	1.00	0.64	-4.41 - 5.68	1.00	0.68	-4.3 - 5.65	1.00	-2.83	-8.84 - 3.18	1.00	-0.87	-3.74 - 2.01	1.00	2.02	-6.18 - 10.23	1.00
		0.5	-0.60	-3.58 - 2.38	1.00	0.81	-2.14 - 3.76	1.00	1.95	-2.99 - 6.89	1.00	-1.44	-5.51 - 2.63	1.00	-0.29	-3.96 - 3.38	1.00	0.16	-4.39 - 4.71	1.00	0.98	-1.32 - 3.28	1.00	-1.45	-7.17 - 4.27	1.00

©2020 Murphy ZR et al. *JAMA Network Open*.

		0.9	-1.22	-3.45 - 1.01	1.00	0.51	-3.1 - 4.13	1.00	0.06	-5.67 - 5.78	1.00	2.10	-2.02 - 6.22	1.00	1.29	-3.8 - 6.38	1.00	-0.34	-7.56 - 6.88	1.00	-0.09	-3.22 - 3.03	1.00	-3.95	-10.96 - 3.05	1.00
	:Num Beds 500+	0.1	-2.60	-6.17 - 0.97	0.47	-1.88	-4.63 - 0.87	0.65	-1.31	-5.78 - 3.15	1.00	1.88	-2.58 - 6.34	1.00	-2.06	-6.03 - 1.9	1.00	-0.80	-4.7 - 3.11	1.00	0.10	-3.17 - 3.38	1.00	0.64	-5.08 - 6.35	1.00
		0.5	-0.08	-2.47 - 2.3	1.00	0.25	-2.11 - 2.61	1.00	0.34	-3.61 - 4.3	1.00	0.53	-2.72 - 3.79	1.00	-2.08	-5.02 - 0.86	0.55	0.17	-3.47 - 3.81	1.00	0.21	-1.62 - 2.05	1.00	-1.53	-6.11 - 3.04	1.00
		0.9	-1.48	-4.09 - 1.14	1.00	0.97	-1.53 - 3.46	1.00	-3.48	-7.95 - 1	0.33	0.94	-2.59 - 4.47	1.00	0.02	-3.65 - 3.68	1.00	-0.09	-4.88 - 4.71	1.00	0.21	-1.5 - 1.91	1.00	-4.14	-9.25 - 0.97	0.25
Cerner		0.1	1.92*	0.10 - 3.73	0.03	-0.95	-2.44 - 0.55	0.90	0.09	-2.32 - 2.5	1.00	1.31	-0.79 - 3.42	0.96	-0.62	-2.76 - 1.52	1.00	0.86	-1.19 - 2.9	1.00	1.30	-0.3 - 2.89	0.25	3.03	-0.02 - 6.07	0.05
		0.5	-0.35	-1.64 - 0.93	1.00	-0.40	-1.67 - 0.88	1.00	-1.54	-3.68 - 0.59	0.49	0.62	-1.14 - 2.38	1.00	-0.47	-2.06 - 1.11	1.00	-0.30	-2.27 - 1.66	1.00	-0.17	-1.16 - 0.83	1.00	1.24	-1.23 - 3.71	1.00
		0.9	-0.61	-2.05 - 0.83	1.00	-0.82	-2.2 - 0.57	1.00	-0.64	-2.96 - 1.67	1.00	-0.22	-2.06 - 1.61	1.00	-1.96*	-3.69 - -0.22	0.01	0.12	-2.3 - 2.55	1.00	-0.14	-1.06 - 0.78	1.00	1.47	-0.9 - 3.83	0.99
	:Num Beds 100-199	0.1	-0.56	-2.79 - 1.67	1.00	2.00*	0.17 - 3.83	0.02	1.30	-1.69 - 4.29	1.00	-1.18	-3.84 - 1.48	1.00	0.77	-1.82 - 3.36	1.00	-2.10	-4.71 - 0.52	0.27	-0.32	-2.2 - 1.56	1.00	-0.62	-4.45 - 3.21	1.00
		0.5	0.45	-1.15 - 2.06	1.00	0.55	-1.04 - 2.14	1.00	0.54	-2.11 - 3.2	1.00	0.03	-2.16 - 2.22	1.00	1.35	-0.63 - 3.32	0.65	-1.67	-4.12 - 0.77	0.65	0.82	-0.41 - 2.06	0.74	-0.33	-3.41 - 2.75	1.00
		0.9	1.40	-0.53 - 3.33	0.48	1.21	-0.51 - 2.94	0.57	0.43	-2.62 - 3.48	1.00	0.28	-2.24 - 2.81	1.00	1.34	-0.97 - 3.65	1.00	-2.20	-5.23 - 0.82	0.47	0.81	-0.38 - 1.99	0.67	-1.38	-4.49 - 1.73	1.00
	:Num Beds 200-299	0.1	0.16	-2.7 - 3.02	1.00	1.58	-0.59 - 3.75	0.48	1.15	-2.83 - 5.12	1.00	-3.72*	-6.95 - -0.5	0.01	1.37	-1.89 - 4.64	1.00	-2.06	-5.48 - 1.36	1.00	0.56	-1.76 - 2.87	1.00	-1.43	-6.14 - 3.28	1.00
		0.5	-0.14	-2.1 - 1.81	1.00	0.34	-1.59 - 2.28	1.00	-0.26	-3.49 - 2.98	1.00	-1.79	-4.46 - 0.88	0.71	1.13	-1.27 - 3.54	1.00	-1.34	-4.32 - 1.64	1.00	0.37	-1.14 - 1.87	1.00	-0.19	-3.94 - 3.56	1.00
		0.9	0.11	-1.97 - 2.2	1.00	-0.25	-2.4 - 1.9	1.00	1.17	-2.3 - 4.64	1.00	-0.16	-2.99 - 2.67	1.00	1.54	-1.17 - 4.26	1.00	-1.91	-5.26 - 1.45	1.00	-0.16	-1.56 - 1.25	1.00	-3.11	-6.64 - 0.43	0.15
	:Num Beds 300-399	0.1	0.98	-2.35 - 4.31	1.00	4.22***	1.42 - 7.02	0.00	4.68*	0.24 - 9.12	0.03	-0.91	-4.56 - 2.74	1.00	2.64	-1.2 - 6.48	0.63	0.08	-3.87 - 4.02	1.00	0.58	-2.02 - 3.17	1.00	-0.93	-7.15 - 5.29	1.00
		0.5	2.06	-0.22 - 4.35	0.12	1.58	-0.68 - 3.84	0.58	1.38	-2.41 - 5.16	1.00	-0.17	-3.29 - 2.95	1.00	1.83	-0.98 - 4.65	0.80	1.59	-1.89 - 5.08	1.00	1.15	-0.61 - 2.91	0.80	-0.52	-4.9 - 3.86	1.00
		0.9	2.14	-0.44 - 4.73	0.22	1.75	-0.65 - 4.15	0.47	1.61	-2.76 - 5.98	1.00	-0.53	-4.02 - 2.95	1.00	4.57***	1.57 - 7.57	0.00	0.20	-4.12 - 4.51	1.00	0.51	-1.06 - 2.07	1.00	-0.83	-4.68 - 3.02	1.00
	:Num Beds 400-499	0.1	-2.87	-8.37 - 2.62	1.00	0.75	-3.67 - 5.16	1.00	1.69	-4 - 7.38	1.00	-2.37	-7.46 - 2.72	1.00	0.24	-4.68 - 5.16	1.00	-2.23	-8.38 - 3.92	1.00	-2.81	-6.01 - 0.39	0.15	-4.41	-12.14 - 3.32	1.00
		0.5	-1.58	-4.75 - 1.58	1.00	-0.66	-3.79 - 2.48	1.00	1.89	-3.36 - 7.14	1.00	-2.60	-6.92 - 1.73	1.00	-0.03	-3.93 - 3.87	1.00	-1.28	-6.12 - 3.55	1.00	0.78	-1.66 - 3.22	1.00	-4.52	-10.6 - 1.56	0.42
		0.9	0.60	-2.06 - 3.27	1.00	-0.13	-4.03 - 3.78	1.00	0.35	-5.94 - 6.64	1.00	1.97	-2.43 - 6.36	1.00	3.31	-2.13 - 8.75	1.00	-1.26	-8.71 - 6.18	1.00	0.13	-3.15 - 3.41	1.00	-5.93	-12.93 - 1.07	0.19
	:Num Beds 500+	0.1	-1.30	-5.14 - 2.55	1.00	-0.89	-3.74 - 1.97	1.00	0.42	-3.98 - 4.81	1.00	-1.04	-5.5 - 3.42	1.00	0.27	-3.82 - 4.36	1.00	-1.54	-5.55 - 2.47	1.00	-0.04	-3.44 - 3.35	1.00	-3.11	-8.97 - 2.75	1.00
		0.5	0.44	-2.03 - 2.9	1.00	-0.01	-2.45 - 2.43	1.00	2.46	-1.63 - 6.55	1.00	1.21	-2.16 - 4.58	1.00	-0.20	-3.23 - 2.84	1.00	0.61	-3.16 - 4.38	1.00	0.43	-1.47 - 2.33	1.00	-1.77	-6.51 - 2.96	1.00
		0.9	0.00	-2.68 - 2.69	1.00	1.27	-1.38 - 3.92	1.00	0.55	-4.15 - 5.26	1.00	1.05	-2.58 - 4.69	1.00	3.47	-0.46 - 7.4	0.14	1.00	-3.87 - 5.87	1.00	1.06	-0.66 - 2.77	1.00	-2.37	-7.82 - 3.08	1.00
McKesson		0.1	0.69	-1.16 - 2.54	1.00	-0.90	-2.47 - 0.66	1.00	-0.11	-2.72 - 2.51	1.00	-0.50	-2.93 - 1.92	1.00	-0.45	-2.77 - 1.88	1.00	1.05	-1.37 - 3.47	1.00	0.12	-1.43 - 1.67	1.00	-2.13	-5.63 - 1.37	1.00
		0.5	0.00	-1.38 - 1.38	1.00	-0.28	-1.64 - 1.09	1.00	0.21	-2.08 - 2.5	1.00	1.63	-0.26 - 3.52	0.17	-0.82	-2.53 - 0.88	1.00	1.17	-0.94 - 3.28	1.00	0.02	-1.04 - 1.09	1.00	2.01	-0.64 - 4.66	0.38
		0.9	-0.86	-2.46 - 0.73	1.00	-1.74*	-3.28 - -0.2	0.01	-0.98	-3.51 - 1.56	1.00	-1.89	-4.02 - 0.25	0.14	-2.36**	-4.39 - -0.33	0.01	-0.87	-3.5 - 1.76	1.00	-0.11	-1.1 - 0.89	1.00	1.33	-1.59 - 4.25	1.00
	:Num Beds 100-199	0.1	-1.08	-3.57 - 1.41	1.00	-0.05	-1.99 - 1.89	1.00	0.57	-2.92 - 4.06	1.00	-2.91*	-5.8 - -0.01	0.05	-0.11	-3.11 - 2.89	1.00	-2.29	-5.26 - 0.67	0.34	0.53	-1.5 - 2.56	1.00	0.83	-3.62 - 5.27	1.00
		0.5	-1.51	-3.27 - 0.24	0.17	-0.27	-2.01 - 1.47	1.00	-1.75	-4.67 - 1.16	1.00	-3.22**	-5.62 - -0.82	0.00	0.09	-2.07 - 2.25	1.00	-3.38**	-6.06 - -0.7	0.00	-0.73	-2.09 - 0.62	1.00	-4.82***	-8.19 - -1.45	0.00
		0.9	0.89	-1.2 - 2.98	1.00	0.72	-1.21 - 2.64	1.00	-0.70	-4.16 - 2.76	1.00	0.64	-2.27 - 3.55	1.00	2.39	-0.26 - 5.04	0.12	-1.49	-4.81 - 1.82	1.00	-0.20	-1.58 - 1.19	1.00	-4.15*	-7.76 - -0.55	0.01
	:Num Beds 200-299	0.1	-2.62	-5.5 - 0.25	0.11	-0.82	-3.25 - 1.6	1.00	-0.33	-4.5 - 3.83	1.00	-1.58	-5.03 - 1.87	1.00	-2.48	-5.99 - 1.03	0.55	-2.24	-5.67 - 1.19	0.79	-1.42	-3.9 - 1.07	1.00	-1.66	-6.37 - 3.05	1.00

©2020 Murphy ZR et al. *JAMA Network Open*.

	0.5	-0.85	-2.97 - 1.26	1.00	-0.23	-2.33 - 1.87	1.00	-1.88	-5.39 - 1.63	1.00	-3.17*	-6.06 - -0.27	0.02	0.80	-1.81 - 3.41	1.00	-2.48	-5.72 - 0.75	0.35	-1.36	-3 - 0.27	0.21	-3.00	-7.07 - 1.06	0.44
	0.9	-1.36	-3.78 - 1.06	1.00	0.30	-1.92 - 2.51	1.00	0.63	-3 - 4.27	1.00	0.68	-2.67 - 4.03	1.00	1.36	-1.93 - 4.65	1.00	-1.72	-5.74 - 2.29	1.00	-1.05	-2.74 - 0.63	0.96	-4.45*	-8.61 - -0.3	0.03
:Num Beds 300-399	0.1	-2.76	-7.25 - 1.73	1.00	-0.55	-4.32 - 3.23	1.00	0.20	-5.64 - 6.03	1.00	-0.54	-5.58 - 4.51	1.00	-0.49	-5.56 - 4.57	1.00	-2.78	-8.37 - 2.81	1.00	0.26	-3.26 - 3.78	1.00	-2.83	-11.13 - 5.46	1.00
	0.5	-1.21	-4.03 - 1.62	1.00	-0.60	-3.4 - 2.19	1.00	-0.92	-5.6 - 3.76	1.00	-2.98	-6.84 - 0.88	0.34	-0.26	-3.74 - 3.22	1.00	-2.93	-7.24 - 1.39	0.67	-0.89	-3.06 - 1.29	1.00	-4.63	-10.06 - 0.79	0.18
	0.9	-1.50	-4.99 - 1.99	1.00	-0.13	-3.18 - 2.92	1.00	-0.51	-5.96 - 4.95	1.00	-1.79	-6.45 - 2.88	1.00	3.31	-0.76 - 7.38	0.25	-1.61	-7.51 - 4.3	1.00	-1.35	-3.32 - 0.63	0.65	-5.50	-11.08 - 0.08	0.06
:Num Beds 400-499	0.1	-3.19	-8.87 - 2.5	1.00	-1.44	-5.63 - 2.76	1.00	-2.10	-9.29 - 5.1	1.00	2.89	-3.75 - 9.52	1.00	-0.79	-7.32 - 5.74	1.00	-3.33	-10.43 - 3.77	1.00	1.95	-1.43 - 5.32	1.00	2.71	-8.48 - 13.9	1.00
	0.5	-0.04	-3.48 - 3.4	1.00	1.13	-2.27 - 4.53	1.00	2.45	-3.25 - 8.15	1.00	-1.61	-6.3 - 3.09	1.00	0.91	-3.33 - 5.14	1.00	0.60	-4.65 - 5.85	1.00	0.19	-2.46 - 2.84	1.00	-3.30	-9.9 - 3.29	1.00
	0.9	2.70	-0.31 - 5.71	0.13	0.46	-4.03 - 4.95	1.00	2.56	-3.58 - 8.7	1.00	3.78	-1.02 - 8.59	0.30	4.09	-1.1 - 9.29	0.30	-0.09	-8.76 - 8.58	1.00	0.96	-2.12 - 4.03	1.00	-2.27	-10.24 - 5.7	1.00
:Num Beds 500+	0.1	-1.97	-7.56 - 3.63	1.00	-3.40	-8.18 - 1.38	0.53	0.46	-7.31 - 8.22	1.00	1.94	-5.03 - 8.92	1.00	-3.44	-10.16 - 3.28	1.00	0.20	-6.78 - 7.18	1.00	0.69	-4.2 - 5.58	1.00	-2.22	-12.12 - 7.68	1.00
	0.5	-0.95	-4.59 - 2.69	1.00	-0.29	-3.89 - 3.31	1.00	2.62	-3.42 - 8.65	1.00	-0.13	-5.11 - 4.84	1.00	-0.29	-4.77 - 4.2	1.00	0.70	-4.86 - 6.26	1.00	-0.36	-3.17 - 2.45	1.00	-1.89	-8.88 - 5.09	1.00
	0.9	0.18	-4.44 - 4.8	1.00	0.57	-4.1 - 5.25	1.00	1.96	-5.19 - 9.11	1.00	3.41	-2.83 - 9.64	1.00	2.45	-3.87 - 8.76	1.00	-1.57	-9 - 5.86	1.00	0.57	-2.44 - 3.58	1.00	-2.47	-11.38 - 6.45	1.00
Years of MU attestation	0.1	0.34	-0.03 - 0.71	0.11	0.12	-0.18 - 0.42	1.00	0.52*	0.03 - 1.02	0.03	0.38	-0.05 - 0.8	0.15	0.45*	0.04 - 0.86	0.02	0.39	-0.03 - 0.82	0.10	0.34*	0.02 - 0.66	0.03	0.71*	0.06 - 1.36	0.02
	0.5	0.09	-0.16 - 0.34	1.00	0.09	-0.15 - 0.34	1.00	0.35	-0.06 - 0.77	0.18	0.30	-0.04 - 0.64	0.13	0.13	-0.17 - 0.44	1.00	0.22	-0.16 - 0.6	1.00	0.07	-0.12 - 0.26	1.00	0.01	-0.46 - 0.49	1.00
	0.9	-0.12	-0.38 - 0.14	1.00	-0.08	-0.33 - 0.18	1.00	0.22	-0.18 - 0.62	1.00	0.24	-0.11 - 0.58	0.67	-0.07	-0.4 - 0.27	1.00	-0.11	-0.54 - 0.32	1.00	0.03	-0.16 - 0.22	1.00	0.06	-0.43 - 0.55	1.00
Total Inpatient Revenue	0.1	0.05	-0.01 - 0.11	0.20	-0.01	-0.05 - 0.03	1.00	0.08	-0.01 - 0.18	0.11	0.02	-0.05 - 0.08	1.00	0.04	-0.04 - 0.12	1.00	-0.02	-0.08 - 0.03	1.00	-0.01	-0.05 - 0.03	1.00	0.06	-0.03 - 0.14	0.68
	0.5	0.00	-0.04 - 0.04	1.00	0.00	-0.04 - 0.04	1.00	-0.01	-0.07 - 0.06	1.00	0.01	-0.04 - 0.06	1.00	0.02	-0.03 - 0.06	1.00	-0.05	-0.12 - 0.01	0.12	0.00	-0.03 - 0.03	1.00	0.05	-0.03 - 0.12	1.00
	0.9	0.02	-0.02 - 0.07	1.00	0.00	-0.05 - 0.05	1.00	0.02	-0.06 - 0.09	1.00	0.04	-0.03 - 0.11	1.00	0.01	-0.06 - 0.07	1.00	0.02	-0.07 - 0.12	1.00	0.00	-0.03 - 0.04	1.00	0.03	-0.05 - 0.1	1.00
Discharges Medicare Percent	0.1	4.75***	1.27 - 8.23	0.00	1.70	-1.1 - 4.51	1.00	0.67	-4.16 - 5.5	1.00	3.71	-0.37 - 7.79	0.11	-1.38	-5.51 - 2.75	1.00	-3.03	-6.98 - 0.92	0.36	1.74	-1.19 - 4.67	1.00	-0.15	-5.96 - 5.65	1.00
	0.5	2.16	-0.24 - 4.57	0.12	1.16	-1.22 - 3.54	1.00	4.62*	0.63 - 8.61	0.01	-0.08	-3.37 - 3.21	1.00	0.65	-2.32 - 3.61	1.00	0.80	-2.88 - 4.47	1.00	1.82	-0.03 - 3.68	0.06	0.59	-4.03 - 5.21	1.00
	0.9	4.61***	1.43 - 7.79	0.00	2.25	-0.57 - 5.07	0.28	6.74***	1.98 - 11.50	0.00	0.97	-3.14 - 5.08	1.00	1.42	-2.15 - 4.99	1.00	1.58	-3.17 - 6.32	1.00	1.80	-0.12 - 3.72	0.09	3.86	-1.66 - 9.37	0.58
Discharges Medicaid Percent	0.1	-5.83***	-9.26 - -2.39	0.00	-1.48	-4.37 - 1.41	1.00	-5.73**	-10.37 - -1.09	0.01	-11.74***	-15.77 - -7.7	0.00	-3.85	-7.92 - 0.22	0.08	-4.74*	-9.25 - -0.24	0.03	-4.46***	-7.23 - -1.69	0.00	-8.87***	-15.15 - -2.6	0.00
	0.5	-4.67***	-7.01 - -2.32	0.00	-1.16	-3.48 - 1.16	1.00	-7.21***	-11.1 - -3.33	0.00	-7.45***	-10.65 - -4.24	0.00	-4.36***	-7.25 - -1.47	0.00	-3.58*	-7.16 - 0	0.05	-2.88***	-4.69 - -1.08	0.00	-8.88***	-13.38 - -4.38	0.00
	0.9	-2.75	-5.63 - 0.13	0.08	-1.35	-3.92 - 1.23	1.00	-4.45	-9.08 - 0.18	0.07	-7.38***	-11.19 - -3.57	0.00	-2.28	-5.78 - 1.22	0.81	-1.88	-6.09 - 2.32	1.00	-1.01	-2.86 - 0.84	1.00	-9.58***	-14.63 - -4.54	0.00
Teaching Hospital Yes	0.1	-0.96***	-1.64 - -0.29	0.00	0.14	-0.47 - 0.75	1.00	-1.14**	-2.05 - -0.22	0.00	-0.60	-1.48 - 0.28	0.67	-0.74	-1.56 - 0.08	0.12	-0.33	-1.23 - 0.58	1.00	-0.89***	-1.46 - -0.33	0.00	-0.99	-2.22 - 0.24	0.27
	0.5	-0.57*	-1.09 - -0.04	0.02	-0.41	-0.93 - 0.11	0.28	-0.63	-1.5 - 0.24	0.49	-0.53	-1.25 - 0.19	0.43	-0.73*	-1.37 - -0.08	0.02	-0.60	-1.4 - 0.21	0.43	-0.73***	-1.13 - -0.32	0.00	-1.19**	-2.2 - -0.18	0.01
	0.9	-0.94***	-1.55 - -0.34	0.00	-0.69**	-1.25 - -0.13	0.00	-1.41***	-2.35 - -0.46	0.00	-1.19***	-2.04 - -0.34	0.00	-0.72	-1.48 - 0.04	0.08	-1.26**	-2.21 - -0.31	0.00	-0.42*	-0.81 - -0.04	0.02	-1.59***	-2.65 - -0.53	0.00
Vendor Count	0.1	-0.20	-0.45 - 0.04	0.21	0.02	-0.19 - 0.23	1.00	-0.24	-0.57 - 0.1	0.53	-0.01	-0.31 - 0.29	1.00	-0.01	-0.29 - 0.27	1.00	-0.30*	-0.58 - -0.02	0.03	0.05	-0.14 - 0.24	1.00	-0.34	-0.75 - 0.08	0.27

©2020 Murphy ZR et al. *JAMA Network Open*.

	0.5	-0.03	-0.19 - 0.14	1.00	0.01	-0.15 - 0.18	1.00	-0.14	-0.41 - 0.13	1.00	0.02	-0.2 - 0.25	1.00	-0.09	-0.29 - 0.11	1.00	0.06	-0.19 - 0.31	1.00	0.13*	0.00 - 0.25	0.04	-0.10	-0.41 - 0.22	1.00
	0.9	0.10	-0.08 - 0.29	1.00	0.14	-0.03 - 0.3	0.20	-0.21	-0.47 - 0.05	0.30	0.18	-0.06 - 0.43	0.36	0.07	-0.17 - 0.32	1.00	0.13	-0.17 - 0.44	1.00	0.02	-0.11 - 0.15	1.00	0.09	-0.21 - 0.39	1.00
Criteria Coverage Percent	0.1	-0.01	-0.02 - 0.01	1.00	-0.01	-0.03 - 0	0.08	0.00	-0.03 - 0.02	1.00	0.01	-0.02 - 0.03	1.00	0.00	-0.03 - 0.02	1.00	0.01	-0.02 - 0.03	1.00	0.00	-0.02 - 0.01	1.00	-0.01	-0.04 - 0.03	1.00
	0.5	-0.02**	-0.03 - 0	0.00	-0.01	-0.02 - 0	0.21	-0.01	-0.03 - 0.01	1.00	-0.01	-0.03 - 0.01	0.65	-0.01	-0.03 - 0	0.14	-0.03**	-0.05 - -0.01	0.00	-0.01	-0.02 - 0	1.00	-0.02	-0.04 - 0.01	0.76
	0.9	-0.01	-0.02 - 0.01	0.85	-0.01	-0.02 - 0.01	1.00	-0.01	-0.04 - 0.01	1.00	-0.02*	-0.04 - 0	0.04	-0.03***	-0.05 - -0.01	0.00	-0.02	-0.04 - 0	0.10	-0.01	-0.02 - 0	0.06	-0.03*	-0.05 - 0	0.05
Magnet Yes	0.1	2.22***	1.24 - 3.20	0.00	1.44***	0.52 - 2.35	0.00	1.88**	0.45 - 3.32	0.00	1.50**	0.23 - 2.77	0.01	1.64**	0.42 - 2.87	0.00	1.88***	0.59 - 3.16	0.00	1.22***	0.37 - 2.07	0.00	2.42***	0.70 - 4.14	0.00
	0.5	1.69***	0.95 - 2.43	0.00	0.92**	0.19 - 1.65	0.00	1.67**	0.44 - 2.89	0.00	2.01***	1.00 - 3.02	0.00	1.53***	0.62 - 2.44	0.00	1.70***	0.57 - 2.82	0.00	0.60*	0.03 - 1.17	0.03	3.63***	2.21 - 5.05	0.00
	0.9	0.99*	0.11 - 1.87	0.01	0.43	-0.39 - 1.25	1.00	1.11	-0.24 - 2.46	0.23	1.12	-0.01 - 2.24	0.05	0.57	-0.52 - 1.66	1.00	1.43*	0.07 - 2.80	0.03	0.62*	0.05 - 1.18	0.02	2.42***	0.88 - 3.96	0.00
CMI	0.1	1.84**	0.45 - 3.24	0.00	1.51***	0.40 - 2.61	0.00	1.04	-0.79 - 2.86	1.00	5.85***	4.18 - 7.53	0.00	0.95	-0.79 - 2.69	1.00	0.40	-1.27 - 2.07	1.00	3.82***	2.48 - 5.15	0.00	7.62***	5.13 - 10.12	0.00
	0.5	1.31**	0.28 - 2.35	0.00	2.33***	1.31 - 3.36	0.00	1.01	-0.71 - 2.73	1.00	5.29***	3.87 - 6.71	0.00	0.57	-0.7 - 1.85	1.00	0.24	-1.35 - 1.82	1.00	2.37***	1.57 - 3.17	0.00	7.30***	5.31 - 9.29	0.00
	0.9	2.47***	1.05 - 3.88	0.00	1.77***	0.54 - 3.00	0.00	1.93	-0.36 - 4.21	0.20	5.59***	3.73 - 7.46	0.00	2.27**	0.51 - 4.03	0.00	1.26	-0.96 - 3.47	1.00	1.63***	0.90 - 2.37	0.00	6.16***	3.95 - 8.38	0.00
const	0.1	74.29***	72.73 - 75.86	0.00	77.05***	75.68 - 78.43	0.00	59.43***	57.20 - 61.66	0.00	46.75***	44.81 - 48.70	0.00	59.13***	57.16 - 61.10	0.00	62.12***	60.19 - 64.05	0.00	83.91***	82.51 - 85.31	0.00	64.07***	61.20 - 66.94	0.00
	0.5	81.36***	80.17 - 82.54	0.00	81.84***	80.67 - 83.02	0.00	69.77***	67.81 - 71.73	0.00	54.18***	52.56 - 55.80	0.00	64.97***	63.51 - 66.42	0.00	68.75***	66.94 - 70.55	0.00	89.26***	88.35 - 90.18	0.00	74.87***	72.60 - 77.14	0.00
	0.9	85.91***	84.50 - 87.31	0.00	86.81***	85.50 - 88.12	0.00	77.55***	75.28 - 79.82	0.00	60.97***	59.17 - 62.78	0.00	72.57***	70.91 - 74.24	0.00	76.81***	74.63 - 78.98	0.00	91.93***	91.04 - 92.82	0.00	83.07***	80.77 - 85.36	0.00
Hospital Ownerhsip (ref Non-profit)																									
Government	0.1	-0.31	-1.21 - 0.58	1.00	0.32	-0.47 - 1.11	1.00	0.02	-1.27 - 1.3	1.00	0.20	-0.91 - 1.32	1.00	-0.78	-1.83 - 0.26	0.40	0.85	-0.27 - 1.97	0.39	-0.45	-1.17 - 0.28	1.00	-0.73	-2.37 - 0.92	1.00
	0.5	-0.15	-0.8 - 0.49	1.00	0.38	-0.26 - 1.02	1.00	-0.09	-1.16 - 0.98	1.00	-0.57	-1.45 - 0.31	0.83	0.10	-0.69 - 0.9	1.00	0.18	-0.8 - 1.17	1.00	-0.30	-0.8 - 0.2	1.00	-0.61	-1.84 - 0.63	1.00
	0.9	-0.07	-0.82 - 0.68	1.00	0.21	-0.48 - 0.9	1.00	0.17	-0.96 - 1.3	1.00	0.01	-0.99 - 1.02	1.00	0.24	-0.65 - 1.13	1.00	0.46	-0.73 - 1.65	1.00	0.11	-0.38 - 0.61	1.00	-0.74	-2.03 - 0.55	1.00
Proprietary	0.1	-1.34***	-2.2 - -0.49	0.00	-0.59	-1.29 - 0.12	0.21	-1.35*	-2.51 - -0.18	0.01	-2.80***	-3.81 - -1.8	0.00	-1.26**	-2.24 - -0.29	0.00	-1.54***	-2.57 - -0.52	0.00	-0.61	-1.31 - 0.08	0.13	-1.72*	-3.2 - -0.23	0.01
	0.5	-1.83***	-2.43 - -1.23	0.00	-0.78**	-1.37 - -0.18	0.00	-1.56***	-2.55 - -0.56	0.00	-3.19***	-4.01 - -2.37	0.00	-1.76***	-2.5 - -1.02	0.00	-1.22**	-2.14 - -0.3	0.00	-0.91***	-1.38 - -0.45	0.00	-2.44***	-3.59 - -1.28	0.00
	0.9	-1.54***	-2.26 - -0.81	0.00	-1.18***	-1.83 - -0.53	0.00	-1.82***	-2.91 - -0.74	0.00	-2.28***	-3.25 - -1.32	0.00	-1.95***	-2.83 - -1.06	0.00	-1.36**	-2.52 - -0.2	0.01	-0.70***	-1.16 - -0.24	0.00	-2.45***	-3.65 - -1.26	0.00
Number of Beds (ref 1-99)																									
100-199	0.1	-0.50	-2 - 1.01	1.00	-2.32***	-3.57 - -1.07	0.00	-3.60***	-5.59 - -1.62	0.00	-1.21	-3.03 - 0.61	0.75	-2.94***	-4.8 - -1.09	0.00	-1.37	-3.13 - 0.39	0.32	-1.00	-2.26 - 0.26	0.29	-3.04*	-5.66 - -0.42	0.01
	0.5	-2.28***	-3.4 - -1.17	0.00	-2.29***	-3.39 - -1.18	0.00	-3.77***	-5.62 - -1.92	0.00	-1.88**	-3.4 - -0.36	0.00	-2.57***	-3.94 - -1.19	0.00	-2.18**	-3.89 - -0.48	0.00	-1.64***	-2.5 - -0.78	0.00	-3.57***	-5.71 - -1.43	0.00
	0.9	-2.73***	-4.09 - -1.37	0.00	-3.18***	-4.37 - -2	0.00	-4.78***	-6.93 - -2.63	0.00	-3.21***	-4.99 - -1.43	0.00	-3.58***	-5.13 - -2.03	0.00	-3.12***	-5.2 - -1.04	0.00	-1.75***	-2.62 - -0.89	0.00	-2.08	-4.19 - 0.03	0.06

©2020 Murphy ZR et al. *JAMA Network Open*.

	200-299	0.1	-2.12	-4.27 - 0.03	0.06	-2.81***	-4.5 - -1.12	0.00	-4.87***	-7.85 - -1.89	0.00	-0.96	-3.48 - 1.57	1.00	-2.98**	-5.46 - -0.5	0.01	-2.47	-4.99 - 0.04	0.06	-2.20**	-3.87 - -0.53	0.00	-5.04***	-8.48 - -1.6	0.00
		0.5	-2.75***	-4.24 - -1.27	0.00	-3.37***	-4.84 - -1.91	0.00	-5.12***	-7.58 - -2.67	0.00	-2.85***	-4.88 - -0.83	0.00	-3.27***	-5.09 - -1.44	0.00	-3.51***	-5.77 - -1.25	0.00	-1.95***	-3.09 - -0.81	0.00	-5.16***	-8 - -2.32	0.00
		0.9	-2.65***	-4.19 - -1.11	0.00	-3.58***	-5.14 - -2.03	0.00	-6.76***	-9.31 - -4.22	0.00	-4.30***	-6.42 - -2.17	0.00	-6.24***	-8.36 - -4.12	0.00	-4.45***	-6.97 - -1.93	0.00	-1.67***	-2.79 - -0.55	0.00	-2.50	-5.02 - 0.01	0.05
	300-399	0.1	-1.89	-4.8 - 1.01	0.81	-4.44***	-6.91 - -1.96	0.00	-6.96***	-10.95 - -2.98	0.00	-4.54***	-7.75 - -1.33	0.00	-3.09	-6.6 - 0.41	0.14	-4.94***	-8.53 - -1.35	0.00	-2.14	-4.38 - 0.09	0.07	-3.82	-9.56 - 1.92	0.73
		0.5	-3.88***	-5.83 - -1.94	0.00	-3.86***	-5.78 - -1.93	0.00	-6.06***	-9.29 - -2.84	0.00	-4.00***	-6.66 - -1.35	0.00	-3.52***	-5.91 - -1.12	0.00	-5.24***	-8.21 - -2.26	0.00	-2.27***	-3.77 - -0.77	0.00	-4.54**	-8.28 - -0.81	0.01
		0.9	-4.96***	-7.17 - -2.76	0.00	-5.05***	-7.1 - -3	0.00	-8.68***	-12.27 - -5.09	0.00	-4.35***	-7.09 - -1.61	0.00	-8.17***	-10.57 - -5.76	0.00	-6.72***	-10.23 - -3.2	0.00	-2.59***	-3.9 - -1.29	0.00	-4.37***	-7.55 - -1.18	0.00
	400-499	0.1	-0.45	-5.5 - 4.6	1.00	-1.56	-5.63 - 2.5	1.00	-6.65**	-12.04 - -1.25	0.01	-2.21	-6.99 - 2.58	1.00	-2.10	-6.84 - 2.64	1.00	-3.15	-8.67 - 2.36	1.00	-0.27	-3.08 - 2.55	1.00	-2.49	-10.12 - 5.14	1.00
		0.5	-1.92	-4.75 - 0.92	0.68	-2.96*	-5.77 - -0.15	0.03	-6.92***	-11.62 - -2.22	0.00	-2.10	-5.97 - 1.77	1.00	-2.24	-5.74 - 1.25	0.86	-5.26**	-9.59 - -0.93	0.01	-2.27*	-4.46 - -0.08	0.04	-3.05	-8.49 - 2.39	1.00
		0.9	-4.03***	-5.91 - -2.15	0.00	-4.01*	-7.49 - -0.53	0.01	-9.20***	-14.51 - -3.89	0.00	-7.01***	-11.08 - -2.93	0.00	-8.34***	-13.37 - -3.32	0.00	-6.81	-13.86 - 0.24	0.07	-2.05	-4.94 - 0.84	0.55	-1.29	-7.5 - 4.92	1.00
	500+	0.1	-0.46	-3.69 - 2.77	1.00	-0.15	-2.79 - 2.5	1.00	-4.78*	-8.95 - -0.61	0.01	-3.14	-7.51 - 1.23	0.51	-0.58	-4.23 - 3.07	1.00	-4.84**	-8.58 - -1.09	0.00	-1.77	-4.81 - 1.27	1.00	-2.13	-7.59 - 3.34	1.00
		0.5	-2.67**	-4.92 - -0.42	0.01	-3.53***	-5.75 - -1.3	0.00	-6.51***	-10.24 - -2.78	0.00	-5.04***	-8.12 - -1.97	0.00	-2.01	-4.78 - 0.77	0.49	-4.79***	-8.23 - -1.35	0.00	-2.43***	-4.17 - -0.7	0.00	-4.82*	-9.14 - -0.5	0.02
		0.9	-3.80***	-6.1 - -1.5	0.00	-5.40***	-7.83 - -2.98	0.00	-8.06***	-11.9 - -4.21	0.00	-6.93***	-10 - -3.86	0.00	-6.95***	-10.62 - -3.28	0.00	-7.38***	-11.63 - -3.13	0.00	-3.39***	-5 - -1.78	0.00	-3.14	-7.58 - 1.31	0.56
Geographic Division (ref East North Central)																										
	East South Central	0.1	0.87	-0.47 - 2.22	0.82	2.75***	1.59 - 3.91	0.00	0.98	-0.9 - 2.87	1.00	0.25	-1.48 - 1.97	1.00	1.32	-0.29 - 2.93	0.24	4.22***	2.48 - 5.97	0.00	-1.67***	-2.75 - -0.59	0.00	2.59*	0.11 - 5.06	0.03
		0.5	0.30	-0.65 - 1.25	1.00	3.14***	2.20 - 4.08	0.00	-0.60	-2.18 - 0.98	1.00	0.36	-0.95 - 1.66	1.00	1.63***	0.46 - 2.81	0.00	3.67***	2.22 - 5.13	0.00	-1.46***	-2.19 - -0.72	0.00	0.78	-1.05 - 2.61	1.00
		0.9	-0.31	-1.37 - 0.75	1.00	2.78***	1.77 - 3.80	0.00	-0.70	-2.41 - 1.01	1.00	-0.17	-1.66 - 1.31	1.00	0.63	-0.71 - 1.96	1.00	2.25**	0.57 - 3.93	0.00	-1.34***	-2.07 - -0.62	0.00	0.24	-1.71 - 2.2	1.00
	Mid Atlantic	0.1	-1.87***	-2.98 - -0.75	0.00	-1.47***	-2.49 - -0.45	0.00	-3.43***	-4.98 - -1.88	0.00	-3.08***	-4.48 - -1.68	0.00	-2.69***	-4 - -1.39	0.00	-5.12***	-6.58 - -3.66	0.00	-1.73***	-2.64 - -0.82	0.00	-5.22***	-7.25 - -3.19	0.00
		0.5	-1.82***	-2.64 - -1	0.00	-1.93***	-2.74 - -1.12	0.00	-3.38***	-4.74 - -2.02	0.00	-3.23***	-4.35 - -2.11	0.00	-2.12***	-3.13 - -1.11	0.00	-3.59***	-4.84 - -2.34	0.00	-1.56***	-2.19 - -0.93	0.00	-5.17***	-6.74 - -3.6	0.00
		0.9	-0.76	-1.75 - 0.23	0.35	-1.23***	-2.12 - -0.33	0.00	-1.43	-2.93 - 0.07	0.08	-2.90***	-4.22 - -1.58	0.00	-1.38*	-2.58 - -0.19	0.01	-3.77***	-5.24 - -2.3	0.00	-1.00***	-1.63 - -0.36	0.00	-3.84***	-5.54 - -2.14	0.00

©2020 Murphy ZR et al. *JAMA Network Open*.

Mountain	0.1	-2.18***	-3.68 - -0.68	0.00	-2.27***	-3.48 - -1.06	0.00	-0.74	-2.75 - 1.26	1.00	-0.40	-2.2 - 1.39	1.00	-0.63	-2.37 - 1.12	1.00	-1.55	-3.45 - 0.35	0.24	-0.49	-1.73 - 0.74	1.00	0.10	-2.46 - 2.66	1.00
	0.5	-2.26***	-3.29 - -1.23	0.00	-1.81***	-2.83 - -0.79	0.00	-1.13	-2.84 - 0.58	0.77	0.13	-1.28 - 1.54	1.00	0.24	-1.03 - 1.52	1.00	-0.81	-2.39 - 0.76	1.00	-0.33	-1.13 - 0.47	1.00	-0.38	-2.36 - 1.6	1.00
	0.9	-2.35***	-3.52 - -1.18	0.00	-1.18*	-2.24 - -0.11	0.02	-1.71	-3.55 - 0.12	0.09	0.18	-1.39 - 1.76	1.00	-0.80	-2.19 - 0.6	1.00	-1.01	-2.81 - 0.78	1.00	-0.50	-1.28 - 0.28	0.85	-1.18	-3.05 - 0.7	0.94
New England	0.1	1.30	-0.4 - 3	0.36	1.76**	0.34 - 3.18	0.00	1.10	-1.09 - 3.3	1.00	1.39	-0.5 - 3.28	0.45	0.90	-1.07 - 2.86	1.00	-0.94	-3.01 - 1.13	1.00	1.36*	0.01 - 2.71	0.05	2.24	-0.65 - 5.13	0.34
	0.5	1.11	-0.05 - 2.27	0.08	0.97	-0.18 - 2.12	0.19	0.07	-1.85 - 2	1.00	2.24***	0.65 - 3.82	0.00	0.67	-0.76 - 2.1	1.00	-2.63***	-4.4 - -0.85	0.00	0.56	-0.34 - 1.46	0.96	-0.04	-2.27 - 2.19	1.00
	0.9	1.11	-0.25 - 2.48	0.24	0.21	-1.02 - 1.43	1.00	-0.59	-2.66 - 1.47	1.00	1.92*	0.07 - 3.77	0.04	0.68	-1.05 - 2.41	1.00	-2.36*	-4.52 - -0.2	0.02	0.18	-0.71 - 1.07	1.00	0.27	-2.15 - 2.7	1.00
Pacific	0.1	-3.30***	-4.51 - -2.09	0.00	-2.58***	-3.62 - -1.54	0.00	-3.26***	-4.97 - -1.55	0.00	-3.17***	-4.62 - -1.71	0.00	-1.46*	-2.91 - -0.01	0.05	-5.24***	-6.79 - -3.69	0.00	-1.61***	-2.59 - -0.63	0.00	-3.10***	-5.32 - -0.88	0.00
	0.5	-2.87***	-3.74 - -2	0.00	-1.55***	-2.41 - -0.69	0.00	-3.17***	-4.61 - -1.73	0.00	-2.42***	-3.6 - -1.23	0.00	-0.67	-1.74 - 0.4	0.97	-3.79***	-5.12 - -2.46	0.00	-1.63***	-2.3 - -0.96	0.00	-2.92***	-4.59 - -1.25	0.00
	0.9	-2.66***	-3.64 - -1.69	0.00	-1.17**	-2.11 - -0.24	0.00	-3.89***	-5.47 - -2.3	0.00	-1.57**	-2.89 - -0.24	0.01	-1.22*	-2.39 - -0.06	0.03	-4.56***	-6.1 - -3.02	0.00	-1.37***	-2.02 - -0.72	0.00	-1.63	-3.34 - 0.07	0.07
South Atlantic	0.1	-1.97***	-2.98 - -0.97	0.00	-0.63	-1.48 - 0.22	0.44	-1.95***	-3.36 - -0.53	0.00	-1.80***	-3.06 - -0.53	0.00	-1.18*	-2.37 - 0	0.05	0.18	-1.13 - 1.49	1.00	-1.99***	-2.78 - -1.2	0.00	-1.72	-3.54 - 0.1	0.08
	0.5	-1.23***	-1.97 - -0.49	0.00	0.53	-0.2 - 1.27	0.48	-1.94***	-3.17 - -0.71	0.00	-1.18**	-2.19 - -0.16	0.01	0.13	-0.78 - 1.04	1.00	0.58	-0.55 - 1.71	1.00	-1.60***	-2.17 - -1.03	0.00	-1.20	-2.62 - 0.22	0.20
	0.9	-1.00*	-1.88 - -0.12	0.01	0.70	-0.09 - 1.49	0.14	-2.24***	-3.54 - -0.95	0.00	-1.08	-2.23 - 0.08	0.09	-0.13	-1.19 - 0.93	1.00	0.64	-0.71 - 2	1.00	-1.30***	-1.87 - -0.73	0.00	-1.41	-2.91 - 0.09	0.09
West North Central	0.1	-1.30*	-2.58 - -0.01	0.05	0.10	-1 - 1.19	1.00	-0.96	-2.63 - 0.7	1.00	-0.50	-2.01 - 1.01	1.00	-0.71	-2.17 - 0.75	1.00	-0.01	-1.55 - 1.53	1.00	-0.51	-1.52 - 0.49	1.00	0.01	-2.23 - 2.25	1.00
	0.5	-1.49***	-2.39 - -0.59	0.00	-0.11	-1 - 0.78	1.00	-1.72*	-3.21 - -0.22	0.01	0.40	-0.83 - 1.63	1.00	-0.91	-2.02 - 0.2	0.24	0.28	-1.09 - 1.66	1.00	-0.30	-0.99 - 0.39	1.00	0.17	-1.56 - 1.9	1.00
	0.9	-1.17*	-2.2 - -0.13	0.01	-0.21	-1.18 - 0.76	1.00	-1.74*	-3.35 - -0.12	0.02	0.39	-1.08 - 1.86	1.00	-0.60	-1.93 - 0.72	1.00	0.56	-1.07 - 2.2	1.00	0.18	-0.54 - 0.89	1.00	-0.57	-2.41 - 1.26	1.00
West South Central	0.1	0.12	-0.99 - 1.23	1.00	1.88***	0.95 - 2.81	0.00	0.81	-0.7 - 2.31	1.00	0.36	-0.94 - 1.66	1.00	1.20	-0.08 - 2.48	0.09	3.34***	1.91 - 4.77	0.00	-2.14***	-3.03 - -1.26	0.00	2.52**	0.64 - 4.41	0.00
	0.5	-0.22	-1.01 - 0.57	1.00	1.55***	0.76 - 2.33	0.00	-0.13	-1.45 - 1.18	1.00	0.72	-0.37 - 1.8	0.76	1.60***	0.62 - 2.58	0.00	3.67***	2.46 - 4.89	0.00	-1.32***	-1.93 - -0.71	0.00	1.50	-0.02 - 3.02	0.06
	0.9	0.19	-0.7 - 1.08	1.00	1.80***	0.98 - 2.62	0.00	1.10	-0.27 - 2.46	0.27	0.84	-0.35 - 2.03	0.54	1.92***	0.86 - 2.98	0.00	2.48***	1.07 - 3.89	0.00	-1.54***	-2.14 - -0.94	0.00	1.05	-0.48 - 2.57	0.64
NCHS Code (ref Large central metro)																									
Large fringe metro	0.1	2.18***	1.23 - 3.13	0.00	0.69	-0.15 - 1.53	0.23	2.67***	1.32 - 4.02	0.00	2.21***	1.07 - 3.35	0.00	2.06***	0.91 - 3.22	0.00	0.63	-0.54 - 1.81	1.00	1.80***	0.99 - 2.62	0.00	2.97***	1.29 - 4.66	0.00
	0.5	1.03***	0.34 - 1.72	0.00	0.47	-0.21 - 1.15	0.61	1.42**	0.28 - 2.56	0.00	1.14**	0.20 - 2.08	0.01	1.08**	0.23 - 1.92	0.00	0.49	-0.56 - 1.54	1.00	0.94***	0.41 - 1.47	0.00	1.70**	0.38 - 3.02	0.00
	0.9	0.42	-0.38 - 1.23	1.00	0.25	-0.46 - 0.96	1.00	0.73	-0.49 - 1.94	1.00	1.36**	0.29 - 2.43	0.00	0.90	-0.07 - 1.88	0.10	0.36	-0.84 - 1.56	1.00	0.51	0 - 1.03	0.06	0.88	-0.5 - 2.26	0.89

©2020 Murphy ZR et al. *JAMA Network Open*.

	Medium metro	0.1	2.06***	1.14 - 2.97	0.00	0.02	-0.79 - 0.83	1.00	2.14***	0.84 - 3.45	0.00	1.67***	0.56 - 2.78	0.00	1.29**	0.20 - 2.39	0.01	0.47	-0.73 - 1.67	1.00	2.35***	1.57 - 3.13	0.00	2.78***	1.14 - 4.42	0.00
		0.5	1.14***	0.47 - 1.80	0.00	0.48	-0.18 - 1.14	0.49	1.28*	0.18 - 2.39	0.01	1.18**	0.27 - 2.09	0.00	1.09**	0.27 - 1.91	0.00	0.18	-0.84 - 1.2	1.00	1.24***	0.73 - 1.76	0.00	1.82***	0.54 - 3.10	0.00
		0.9	0.63	-0.16 - 1.41	0.27	0.23	-0.46 - 0.92	1.00	1.24*	0.04 - 2.44	0.04	1.39**	0.35 - 2.44	0.00	0.91	-0.02 - 1.84	0.06	0.81	-0.35 - 1.97	0.60	0.84***	0.33 - 1.35	0.00	0.55	-0.82 - 1.92	1.00
	Small metro	0.1	2.35***	1.25 - 3.46	0.00	0.62	-0.36 - 1.6	0.89	4.00***	2.47 - 5.53	0.00	2.40***	1.08 - 3.72	0.00	2.46***	1.18 - 3.74	0.00	0.78	-0.61 - 2.16	1.00	2.44***	1.53 - 3.36	0.00	3.14***	1.13 - 5.14	0.00
		0.5	1.52***	0.71 - 2.32	0.00	0.60	-0.2 - 1.39	0.39	2.51***	1.18 - 3.84	0.00	0.78	-0.32 - 1.88	0.53	1.37***	0.38 - 2.36	0.00	0.80	-0.42 - 2.03	0.79	1.17***	0.55 - 1.79	0.00	1.41	-0.13 - 2.95	0.11
		0.9	0.70	-0.28 - 1.69	0.53	0.46	-0.39 - 1.31	1.00	1.45	-0.02 - 2.92	0.06	0.95	-0.29 - 2.18	0.35	0.69	-0.48 - 1.86	1.00	0.03	-1.46 - 1.52	1.00	0.70*	0.07 - 1.33	0.02	0.79	-0.83 - 2.4	1.00
	Micropolitan	0.1	2.67***	1.45 - 3.89	0.00	1.01	-0.04 - 2.07	0.07	5.43***	3.71 - 7.15	0.00	1.42	-0.03 - 2.87	0.06	2.72***	1.29 - 4.14	0.00	0.50	-1.02 - 2.02	1.00	2.73***	1.72 - 3.73	0.00	1.43	-0.74 - 3.61	0.76
		0.5	1.52***	0.67 - 2.37	0.00	1.03**	0.19 - 1.87	0.01	2.89***	1.49 - 4.30	0.00	-0.36	-1.52 - 0.81	1.00	1.25**	0.20 - 2.29	0.01	0.72	-0.58 - 2.01	1.00	0.87**	0.22 - 1.53	0.00	-0.04	-1.68 - 1.59	1.00
		0.9	1.08*	0.10 - 2.07	0.02	1.04**	0.16 - 1.92	0.01	2.51***	0.92 - 4.10	0.00	0.49	-0.81 - 1.78	1.00	1.02	-0.13 - 2.17	0.14	0.26	-1.26 - 1.78	1.00	0.60	-0.02 - 1.23	0.06	-0.86	-2.58 - 0.85	1.00
	Noncore	0.1	3.42***	1.63 - 5.22	0.00	2.71***	1.22 - 4.20	0.00	6.69***	4.36 - 9.03	0.00	1.66	-0.49 - 3.81	0.35	4.49***	2.47 - 6.51	0.00	1.83	-0.4 - 4.06	0.23	1.70**	0.34 - 3.05	0.00	4.52***	1.42 - 7.62	0.00
		0.5	2.45***	1.22 - 3.67	0.00	2.74***	1.52 - 3.95	0.00	4.60***	2.56 - 6.63	0.00	0.41	-1.26 - 2.09	1.00	3.60***	2.08 - 5.11	0.00	2.01*	0.13 - 3.88	0.03	1.28**	0.34 - 2.23	0.00	2.52*	0.16 - 4.88	0.03
		0.9	3.30***	1.96 - 4.64	0.00	3.98***	2.76 - 5.20	0.00	3.26***	1.04 - 5.49	0.00	3.98***	2.23 - 5.73	0.00	3.87***	2.25 - 5.50	0.00	0.56	-1.57 - 2.7	1.00	0.76	-0.17 - 1.69	0.24	2.34	-0.02 - 4.69	0.05

CPOE = computerized provider order entry, MU = meaningful use, CMI = case mix index, NCHS = national center for health statistics

eTable 7. Adjusted Quantile Regression Results for Medicare Spending per Beneficiary (MSPB) and Hospital-Acquired Infection (HAI) Outcomes at 0.1, 0.5, and 0.9 Quantiles

Variable	τ	MSPB			CLABSI			CAUTI			SSI-Colon			MRSA Bacteremia			C diff Infection		
		**β**	**99.6% CI**	**p**	**β**	**99.6% CI**	**p**	**β**	**99.6% CI**	**p**	**β**	**99.6% CI**	**p**	**β**	**99.6% CI**	**p**	**β**	**99.6% CI**	**p**
CPOE for Medication Orders (by order)	0.1	-0.44	-1.42 - 0.54	1.00	0.01	-4.13 - 4.15	1.00	0.10	-5.24 - 5.43	1.00	0.78	-7.23 - 8.79	1.00	2.23	-6.68 - 11.13	1.00	2.87	-3.07 - 8.8	1.00
	0.5	-0.57	-1.34 - 0.2	0.43	-1.71	-9.21 - 5.79	1.00	3.73	-4.01 - 11.48	1.00	-3.61	-17.37 - 10.15	1.00	0.79	-12.52 - 14.11	1.00	2.75	-2.54 - 8.04	1.00
	0.9	-0.49	-1.37 - 0.4	1.00	-3.95	-18.06 - 10.16	1.00	7.04	-3.58 - 17.66	0.75	-10.47	-28.02 - 7.09	1.00	-8.11	-28.98 - 12.77	1.00	-4.65	-12.46 - 3.16	1.00
:MEDITECH	0.1	-0.77	-2.15 - 0.61	1.00	-0.14	-6.14 - 5.87	1.00	0.04	-7.22 - 7.3	1.00	0.41	-11.58 - 12.4	1.00	-1.65	-15 - 11.69	1.00	1.31	-6.79 - 9.41	1.00
	0.5	0.16	-0.97 - 1.29	1.00	-7.59	-18.04 - 2.87	0.48	0.22	-10.69 - 11.14	1.00	5.14	-13.75 - 24.04	1.00	-11.82	-30.96 - 7.32	1.00	-2.22	-9.88 - 5.45	1.00
	0.9	-0.49	-1.85 - 0.88	1.00	-4.23	-24.45 - 15.98	1.00	-19.75**	-35.9 - -3.6	0.01	-1.70	-23.93 - 20.52	1.00	25.81	-4.7 - 56.32	0.19	-3.34	-14.34 - 7.66	1.00
:Epic	0.1	-0.41	-2.36 - 1.54	1.00	0.62	-5.96 - 7.2	1.00	0.01	-8.19 - 8.22	1.00	5.64	-13.84 - 25.11	1.00	-3.08	-19.83 - 13.68	1.00	-1.42	-13.81 - 10.96	1.00
	0.5	-1.00	-2.75 - 0.74	1.00	-2.97	-16.8 - 10.86	1.00	-0.82	-16.05 - 14.42	1.00	15.78	-9.46 - 41.01	0.96	-13.29	-38.9 - 12.31	1.00	-0.84	-12.59 - 10.9	1.00
	0.9	-0.46	-2.92 - 2.01	1.00	3.11	-21.5 - 27.73	1.00	-20.42	-44.1 - 3.25	0.17	5.32	-23.97 - 34.6	1.00	-21.20	-59.62 - 17.22	1.00	2.48	-15.11 - 20.08	1.00
:Cerner	0.1	0.29	-1.42 - 1.99	1.00	0.14	-6.32 - 6.61	1.00	-0.25	-7.53 - 7.03	1.00	0.44	-12.02 - 12.89	1.00	-4.18	-17.55 - 9.19	1.00	-4.22	-13.57 - 5.12	1.00
	0.5	-0.04	-1.3 - 1.23	1.00	0.25	-10.53 - 11.04	1.00	-0.84	-12.38 - 10.71	1.00	12.42	-6.99 - 31.84	0.87	-0.80	-20.34 - 18.74	1.00	-1.48	-10.05 - 7.09	1.00
	0.9	-0.21	-1.54 - 1.13	1.00	5.29	-15.08 - 25.66	1.00	-4.85	-22.34 - 12.64	1.00	14.83	-10.46 - 40.13	1.00	11.29	-19.21 - 41.79	1.00	11.56	-1.84 - 24.95	0.17
:McKesson	0.1	1.60	-0.05 - 3.25	0.07	0.35	-6.47 - 7.16	1.00	-0.24	-7.78 - 7.29	1.00	0.78	-10.6 - 12.16	1.00	3.12	-11.45 - 17.69	1.00	-4.25	-13.71 - 5.21	1.00
	0.5	1.11	-0.18 - 2.41	0.17	4.03	-6.84 - 14.89	1.00	-4.12	-15.82 - 7.58	1.00	8.72	-11.42 - 28.86	1.00	5.31	-15.65 - 26.27	1.00	-9.06*	-17.81 - -0.31	0.04
	0.9	1.44	-0.15 - 3.04	0.12	13.97	-7.88 - 35.81	0.87	-11.39	-32.42 - 9.63	1.00	11.94	-13.69 - 37.57	1.00	39.54***	11.84 - 67.25	0.00	15.92***	4.28 - 27.56	0.00
CPOE for Laboratory Orders	0.1	0.21	-0.43 - 0.86	1.00	0.04	-2.9 - 2.99	1.00	0.06	-3.31 - 3.43	1.00	2.53	-4.16 - 9.22	1.00	-0.47	-7.91 - 6.98	1.00	-0.49	-4.44 - 3.45	1.00
	0.5	0.02	-0.51 - 0.55	1.00	3.10	-1.94 - 8.14	1.00	-2.65	-7.93 - 2.64	1.00	1.64	-7.85 - 11.13	1.00	1.76	-7.59 - 11.1	1.00	-1.96	-5.6 - 1.67	1.00
	0.9	-0.14	-0.75 - 0.47	1.00	0.09	-9.55 - 9.73	1.00	1.08	-7.03 - 9.19	1.00	6.33	-5.88 - 18.54	1.00	11.73	-5.93 - 29.38	0.74	3.34	-2.76 - 9.44	1.00
:MEDITECH	0.1	0.12	-0.88 - 1.11	1.00	0.00	-4.87 - 4.87	1.00	-0.14	-5.56 - 5.28	1.00	-0.81	-11.56 - 9.94	1.00	-1.32	-12.34 - 9.69	1.00	1.99	-4.05 - 8.03	1.00
	0.5	-0.15	-1.02 - 0.72	1.00	-1.32	-9.42 - 6.78	1.00	-0.18	-8.74 - 8.38	1.00	-3.39	-18.15 - 11.37	1.00	-11.59	-26.35 - 3.17	0.31	1.48	-4.43 - 7.39	1.00
	0.9	0.11	-0.92 - 1.15	1.00	8.51	-8 - 25.02	1.00	0.94	-13.24 - 15.12	1.00	-8.78	-26.03 - 8.46	1.00	-24.91	-50.8 - 0.99	0.07	-0.96	-9.69 - 7.76	1.00
:Epic	0.1	0.74	-0.63 - 2.11	1.00	-0.28	-4.58 - 4.03	1.00	0.14	-5.18 - 5.46	1.00	-3.08	-17.03 - 10.86	1.00	0.45	-12.68 - 13.59	1.00	0.85	-9.49 - 11.18	1.00
	0.5	0.82	-0.49 - 2.14	0.95	7.14	-2.88 - 17.16	0.53	0.85	-10.3 - 12.01	1.00	-10.25	-28.27 - 7.78	1.00	7.06	-12.08 - 26.2	1.00	5.82	-3.03 - 14.67	0.77
	0.9	0.43	-1.56 - 2.43	1.00	4.40	-14.24 - 23.03	1.00	8.46	-7.94 - 24.86	1.00	2.71	-20.01 - 25.42	1.00	4.00	-28.43 - 36.43	1.00	4.58	-9.58 - 18.74	1.00
:Cerner	0.1	-0.38	-1.42 - 0.67	1.00	-0.08	-4.34 - 4.19	1.00	-0.21	-4.49 - 4.07	1.00	-4.14	-13.02 - 4.74	1.00	1.48	-8.14 - 11.1	1.00	1.57	-3.59 - 6.72	1.00
	0.5	-0.27	-1.07 - 0.54	1.00	-2.51	-9.36 - 4.34	1.00	1.03	-6.36 - 8.42	1.00	-11.93	-24.72 - 0.85	0.09	1.21	-11.54 - 13.96	1.00	2.71	-2.76 - 8.19	1.00

©2020 Murphy ZR et al. *JAMA Network Open*.

		0.9	0.29	-0.59 - 1.18	1.00	1.55	-11.62 - 14.72	1.00	2.45	-9.37 - 14.28	1.00	-9.50	-27.04 - 8.04	1.00	-11.18	-33.01 - 10.65	1.00	-7.76	-16.78 - 1.27	0.17
	:McKesson	0.1	-0.76	-1.75 - 0.23	0.36	0.17	-3.8 - 4.14	1.00	-0.07	-4.27 - 4.14	1.00	-2.72	-11.93 - 6.49	1.00	0.22	-10.04 - 10.48	1.00	0.34	-5.79 - 6.47	1.00
		0.5	0.07	-0.75 - 0.89	1.00	-1.13	-8.2 - 5.94	1.00	2.48	-5.05 - 10.02	1.00	1.95	-11.02 - 14.92	1.00	1.19	-12.13 - 14.5	1.00	2.25	-3.33 - 7.84	1.00
		0.9	-0.04	-0.98 - 0.9	1.00	3.23	-9.55 - 16.01	1.00	4.03	-7.99 - 16.04	1.00	-4.00	-19.85 - 11.85	1.00	-15.90	-38.72 - 6.92	0.59	-1.52	-9.49 - 6.46	1.00
Patient Electronic Access (Available)		0.1	-0.03	-0.7 - 0.65	1.00	-0.30	-3.09 - 2.49	1.00	-0.11	-3.47 - 3.25	1.00	2.71	-3.86 - 9.28	1.00	-1.12	-8.26 - 6.02	1.00	1.19	-3.37 - 5.76	1.00
		0.5	0.18	-0.39 - 0.76	1.00	-5.24*	-10.44 - -0.04	0.05	2.02	-3.51 - 7.56	1.00	4.95	-4.91 - 14.81	1.00	3.79	-5.78 - 13.36	1.00	0.56	-3.36 - 4.47	1.00
		0.9	-0.08	-0.82 - 0.65	1.00	4.07	-3.48 - 11.63	1.00	-4.03	-13.07 - 5	1.00	16.88**	3.97 - 29.79	0.00	4.34	-9.38 - 18.06	1.00	1.42	-3.87 - 6.71	1.00
	:MEDITECH	0.1	0.09	-0.8 - 0.98	1.00	0.25	-3.49 - 3.99	1.00	0.19	-3.89 - 4.28	1.00	-2.16	-10.88 - 6.57	1.00	0.80	-8.29 - 9.88	1.00	-2.00	-7.48 - 3.48	1.00
		0.5	0.10	-0.68 - 0.88	1.00	7.85*	1.00 - 14.69	0.01	-3.84	-11.07 - 3.39	1.00	0.19	-12.31 - 12.68	1.00	-1.50	-13.88 - 10.89	1.00	1.00	-4.25 - 6.26	1.00
		0.9	0.54	-0.43 - 1.51	1.00	-1.89	-12.61 - 8.83	1.00	-10.06	-22.4 - 2.29	0.25	-13.84	-29.69 - 2	0.15	3.43	-14.58 - 21.45	1.00	6.01	-1.69 - 13.71	0.32
	:Epic	0.1	-1.22	-3.24 - 0.79	1.00	0.28	-9.57 - 10.12	1.00	0.79	-6.57 - 8.16	1.00	-4.63	-19.78 - 10.51	1.00	-2.63	-18.62 - 13.37	1.00	-1.09	-11.21 - 9.03	1.00
		0.5	-1.75*	-3.41 - -0.09	0.03	2.83	-10.66 - 16.32	1.00	-4.35	-19.16 - 10.47	1.00	-15.57	-39.64 - 8.5	0.83	0.61	-23.41 - 24.63	1.00	4.27	-7.12 - 15.67	1.00
		0.9	-2.21*	-4.34 - -0.08	0.04	-3.86	-27.8 - 20.07	1.00	0.59	-24.71 - 25.9	1.00	-12.17	-44.1 - 19.76	1.00	7.56	-26.32 - 41.44	1.00	-3.54	-20.05 - 12.96	1.00
	:Cerner	0.1	0.14	-0.82 - 1.09	1.00	0.05	-3.62 - 3.72	1.00	0.33	-3.91 - 4.56	1.00	-3.08	-11.49 - 5.33	1.00	1.17	-8.46 - 10.8	1.00	-0.74	-6.81 - 5.33	1.00
		0.5	0.30	-0.52 - 1.12	1.00	4.62	-2.12 - 11.37	0.64	-1.98	-9.3 - 5.34	1.00	-4.35	-16.92 - 8.21	1.00	3.34	-9.08 - 15.77	1.00	-1.50	-7.05 - 4.06	1.00
		0.9	0.55	-0.43 - 1.53	1.00	-4.44	-14.94 - 6.07	1.00	0.69	-11.12 - 12.51	1.00	-19.96**	-37.11 - -2.82	0.01	-1.53	-20.34 - 17.28	1.00	-0.77	-8.56 - 7.03	1.00
	:McKesson	0.1	0.38	-0.79 - 1.56	1.00	0.57	-4.97 - 6.1	1.00	0.09	-5.04 - 5.23	1.00	-2.06	-12.97 - 8.85	1.00	-0.28	-12.77 - 12.2	1.00	-4.08	-11.46 - 3.3	1.00
		0.5	-0.54	-1.53 - 0.45	1.00	5.18	-3.54 - 13.89	1.00	3.64	-5.82 - 13.1	1.00	-4.90	-20.33 - 10.53	1.00	-0.67	-16.76 - 15.42	1.00	2.46	-4.2 - 9.13	1.00
		0.9	-1.43**	-2.58 - -0.27	0.00	-7.46	-21.75 - 6.83	1.00	4.89	-11.84 - 21.62	1.00	-21.03**	-38.02 - -4.05	0.00	21.32	-6.41 - 49.05	0.35	2.60	-6.91 - 12.12	1.00
Patient Electronic Access (Accessed)		0.1	-0.07	-0.91 - 0.76	1.00	-0.08	-2.69 - 2.53	1.00	0.01	-3.35 - 3.37	1.00	4.84	-8.79 - 18.46	1.00	4.37	-7.25 - 15.98	1.00	0.45	-4.77 - 5.68	1.00
		0.5	-0.42	-1.03 - 0.2	0.68	3.08	-2.71 - 8.87	1.00	1.52	-4.64 - 7.68	1.00	0.01	-12.02 - 12.03	1.00	-2.40	-13.15 - 8.35	1.00	-2.35	-6.48 - 1.78	1.00
		0.9	-0.71	-1.68 - 0.26	0.45	-1.52	-19.19 - 16.15	1.00	0.27	-12.64 - 13.19	1.00	15.31	-1.56 - 32.18	0.11	-2.85	-16.85 - 11.15	1.00	-5.78	-14.07 - 2.51	0.59
	:MEDITECH	0.1	0.73	-0.62 - 2.08	1.00	0.40	-5.09 - 5.9	1.00	-0.03	-8.71 - 8.66	1.00	-7.25	-22.48 - 7.99	1.00	-2.72	-15.76 - 10.31	1.00	1.50	-6.3 - 9.3	1.00
		0.5	0.29	-0.82 - 1.41	1.00	-4.29	-13.79 - 5.2	1.00	-1.74	-11.98 - 8.5	1.00	-12.05	-29.96 - 5.87	0.70	13.93	-3.61 - 31.48	0.29	7.65*	0.15 - 15.15	0.04
		0.9	1.37*	0.16 - 2.57	0.01	-8.19	-29.14 - 12.75	1.00	6.30	-9.08 - 21.68	1.00	-31.49	-67.35 - 4.37	0.15	0.18	-24.18 - 24.54	1.00	18.73***	6.41 - 31.05	0.00
	:Epic	0.1	0.24	-0.81 - 1.29	1.00	-0.04	-3.61 - 3.54	1.00	-0.37	-4.65 - 3.92	1.00	-4.71	-18.51 - 9.08	1.00	-7.31	-20.32 - 5.71	1.00	-1.69	-7.84 - 4.46	1.00
		0.5	0.24	-0.57 - 1.05	1.00	-4.66	-11.8 - 2.48	0.80	-2.81	-10.61 - 4.98	1.00	4.28	-9.71 - 18.27	1.00	0.95	-12.33 - 14.23	1.00	2.93	-2.55 - 8.42	1.00
		0.9	0.45	-0.75 - 1.64	1.00	-1.97	-20.55 - 16.61	1.00	4.43	-10.77 - 19.63	1.00	-16.19	-36.44 - 4.06	0.28	-7.72	-27.31 - 11.86	1.00	5.53	-4.22 - 15.27	1.00
	:Cerner	0.1	-1.06	-2.27 - 0.15	0.15	0.30	-4.27 - 4.88	1.00	0.21	-5.2 - 5.63	1.00	-5.55	-18.29 - 7.19	1.00	-4.77	-18.89 - 9.35	1.00	-0.23	-7.39 - 6.93	1.00
		0.5	-0.44	-1.36 - 0.47	1.00	1.25	-6.45 - 8.95	1.00	1.48	-6.95 - 9.9	1.00	-3.28	-17.42 - 10.86	1.00	-7.77	-21.86 - 6.31	1.00	-1.78	-7.92 - 4.37	1.00

©2020 Murphy ZR et al. *JAMA Network Open*.

		0.9	-0.30	-1.56 - 0.97	1.00	1.88	-14.66 - 18.42	1.00	-9.16	-23.03 - 4.71	0.76	-15.70	-34.38 - 2.98	0.20	-10.60	-26.94 - 5.74	0.82	2.32	-9.08 - 13.72	1.00
	:McKesson	0.1	0.02	-1.39 - 1.42	1.00	-0.09	-8.05 - 7.86	1.00	0.11	-6.01 - 6.23	1.00	-2.19	-23.55 - 19.18	1.00	-1.33	-15.75 - 13.1	1.00	4.17	-7.63 - 15.96	1.00
		0.5	0.02	-1.24 - 1.28	1.00	-4.35	-14.46 - 5.77	1.00	3.88	-6.85 - 14.62	1.00	-3.57	-22.81 - 15.68	1.00	4.37	-14.82 - 23.55	1.00	1.48	-7 - 9.96	1.00
		0.9	0.21	-2 - 2.43	1.00	17.93	-0.78 - 36.64	0.07	12.53	-4 - 29.07	0.38	-6.69	-31.82 - 18.44	1.00	38.35***	16.42 - 60.28	0.00	-3.54	-17.81 - 10.72	1.00
Patient-Specific Education Resources		0.1	-0.40**	-0.74 - -0.06	0.01	0.00	-1.38 - 1.39	1.00	-0.03	-1.51 - 1.44	1.00	-0.41	-3.9 - 3.07	1.00	-1.33	-5.25 - 2.58	1.00	-1.13	-3.27 - 1.02	1.00
		0.5	-0.13	-0.41 - 0.16	1.00	-0.68	-3.16 - 1.81	1.00	-0.44	-3.1 - 2.21	1.00	2.05	-2.48 - 6.58	1.00	-2.01	-6.61 - 2.59	1.00	-0.53	-2.47 - 1.42	1.00
		0.9	0.17	-0.2 - 0.53	1.00	2.97	-1.73 - 7.66	0.91	-0.52	-4.6 - 3.55	1.00	-5.71	-11.78 - 0.37	0.09	0.36	-7.17 - 7.89	1.00	-1.60	-4.66 - 1.47	1.00
	:MEDITECH	0.1	0.21	-0.36 - 0.79	1.00	-0.11	-2.53 - 2.31	1.00	0.04	-2.3 - 2.37	1.00	0.06	-5.37 - 5.5	1.00	0.84	-5.12 - 6.81	1.00	1.44	-1.71 - 4.58	1.00
		0.5	0.16	-0.29 - 0.6	1.00	0.99	-2.88 - 4.87	1.00	0.32	-3.8 - 4.45	1.00	0.78	-6 - 7.56	1.00	-0.92	-8.23 - 6.4	1.00	-0.98	-3.97 - 2	1.00
		0.9	-0.29	-0.83 - 0.24	1.00	-1.21	-8.15 - 5.74	1.00	5.22	-0.77 - 11.2	0.16	8.88	-0.14 - 17.91	0.06	-4.12	-15.92 - 7.69	1.00	-1.27	-5.64 - 3.1	1.00
	:Epic	0.1	-0.17	-1.02 - 0.68	1.00	-0.01	-2.81 - 2.79	1.00	-0.30	-3.59 - 2.99	1.00	-3.93	-10.32 - 2.46	1.00	1.58	-5.77 - 8.94	1.00	2.93	-2.16 - 8.03	1.00
		0.5	-0.02	-0.7 - 0.66	1.00	-2.77	-7.98 - 2.45	1.00	2.14	-3.88 - 8.16	1.00	-2.55	-12.23 - 7.13	1.00	-2.93	-12.32 - 6.47	1.00	-1.32	-5.91 - 3.27	1.00
		0.9	0.37	-0.48 - 1.22	1.00	-3.31	-13.93 - 7.31	1.00	-1.11	-10.91 - 8.7	1.00	7.67	-5.95 - 21.28	1.00	-8.23	-23.69 - 7.24	1.00	1.82	-5.59 - 9.23	1.00
	:Cerner	0.1	0.54	-0.09 - 1.16	0.17	0.05	-2.12 - 2.22	1.00	-0.13	-2.42 - 2.15	1.00	-0.65	-5.69 - 4.38	1.00	0.48	-5.14 - 6.11	1.00	0.99	-2.56 - 4.54	1.00
		0.5	0.19	-0.3 - 0.68	1.00	1.90	-1.92 - 5.71	1.00	-2.74	-6.97 - 1.49	0.83	-3.92	-10.81 - 2.97	1.00	3.49	-3.48 - 10.47	1.00	2.46	-0.82 - 5.73	0.40
		0.9	-0.34	-0.9 - 0.23	1.00	-1.94	-8.92 - 5.03	1.00	-3.62	-10.39 - 3.15	1.00	-2.88	-11.47 - 5.71	1.00	3.70	-7.17 - 14.58	1.00	3.18	-1.73 - 8.09	0.82
	:McKesson	0.1	0.33	-0.19 - 0.85	0.91	-0.19	-2.48 - 2.11	1.00	0.08	-2.12 - 2.27	1.00	0.32	-4.51 - 5.15	1.00	0.72	-4.96 - 6.41	1.00	0.75	-2.51 - 4.01	1.00
		0.5	0.23	-0.24 - 0.7	1.00	-0.66	-4.42 - 3.11	1.00	1.84	-2.29 - 5.97	1.00	-2.60	-9.4 - 4.2	1.00	3.31	-3.96 - 10.58	1.00	0.48	-2.66 - 3.62	1.00
		0.9	0.16	-0.39 - 0.71	1.00	-5.15	-11.59 - 1.28	0.27	-0.12	-6.72 - 6.47	1.00	9.65	-0.09 - 19.39	0.05	-1.86	-14.27 - 10.56	1.00	-0.33	-5.23 - 4.57	1.00
Medication Reconciliation		0.1	0.29	-0.38 - 0.96	1.00	-0.04	-2.94 - 2.85	1.00	0.02	-2.92 - 2.96	1.00	-0.83	-7.1 - 5.45	1.00	0.04	-7.55 - 7.64	1.00	-2.09	-6.52 - 2.34	1.00
		0.5	0.03	-0.53 - 0.6	1.00	4.10	-0.89 - 9.1	0.23	0.42	-4.83 - 5.68	1.00	-2.73	-12.08 - 6.62	1.00	4.67	-4.75 - 14.09	1.00	-2.55	-6.41 - 1.3	0.75
		0.9	-0.03	-0.68 - 0.62	1.00	-7.98	-16.35 - 0.38	0.08	-1.83	-9.93 - 6.28	1.00	8.95	-2.72 - 20.62	0.36	6.83	-7.12 - 20.78	1.00	2.87	-2.62 - 8.35	1.00
	:MEDITECH	0.1	-0.53	-1.48 - 0.42	1.00	-0.12	-4.12 - 3.89	1.00	-0.17	-4.36 - 4.03	1.00	1.58	-7 - 10.16	1.00	-0.54	-10.19 - 9.11	1.00	0.21	-5.35 - 5.78	1.00
		0.5	-0.15	-0.94 - 0.64	1.00	-5.62	-12.49 - 1.25	0.24	-2.07	-9.29 - 5.15	1.00	-2.67	-15.02 - 9.68	1.00	-4.91	-17.75 - 7.93	1.00	-1.08	-6.41 - 4.25	1.00
		0.9	0.15	-0.77 - 1.08	1.00	4.99	-6.71 - 16.7	1.00	-2.45	-13.79 - 8.89	1.00	-15.78	-32.05 - 0.5	0.07	1.25	-17.31 - 19.81	1.00	-3.79	-11.2 - 3.62	1.00
	:Epic	0.1	0.07	-1.63 - 1.77	1.00	0.11	-5.43 - 5.65	1.00	0.85	-5.34 - 7.04	1.00	-1.83	-13.63 - 9.96	1.00	1.04	-12.95 - 15.04	1.00	1.74	-6.01 - 9.49	1.00
		0.5	0.33	-0.9 - 1.56	1.00	1.49	-8.05 - 11.02	1.00	4.52	-6.32 - 15.36	1.00	-1.18	-18.52 - 16.16	1.00	3.63	-13.92 - 21.17	1.00	4.42	-3.9 - 12.74	1.00
		0.9	0.12	-1.42 - 1.67	1.00	6.51	-10.95 - 23.96	1.00	-15.30	-33.19 - 2.6	0.18	-14.78	-37 - 7.44	0.73	13.51	-14.26 - 41.28	1.00	0.66	-11.83 - 13.15	1.00
	:Cerner	0.1	-0.02	-1.23 - 1.2	1.00	0.39	-3.99 - 4.77	1.00	-0.32	-5.04 - 4.41	1.00	0.03	-9.68 - 9.74	1.00	0.09	-10.98 - 11.15	1.00	4.26	-1.89 - 10.41	0.61
		0.5	0.12	-0.87 - 1.1	1.00	-2.52	-10.43 - 5.38	1.00	-5.03	-13.5 - 3.43	1.00	3.34	-11.15 - 17.82	1.00	-4.89	-20.09 - 10.31	1.00	1.71	-4.94 - 8.35	1.00

©2020 Murphy ZR et al. *JAMA Network Open*.

	0.9	-0.36	-1.43 - 0.71	1.00	0.21	-14.75 - 15.16	1.00	-5.63	-18.37 - 7.12	1.00	-7.10	-25.71 - 11.5	1.00	-31.36**	-54.8 - -7.91	0.00	-0.87	-10.9 - 9.15	1.00
:McKesson	0.1	-1.42*	-2.69 - -0.14	0.02	-0.19	-6.12 - 5.73	1.00	-0.11	-5.44 - 5.22	1.00	-1.57	-11.59 - 8.45	1.00	-0.59	-14.41 - 13.23	1.00	3.71	-5.17 - 12.6	1.00
	0.5	-1.35**	-2.42 - -0.28	0.00	-8.39	-17.18 - 0.4	0.08	0.38	-9.35 - 10.1	1.00	-1.90	-18.56 - 14.75	1.00	-8.05	-25.18 - 9.07	1.00	3.69	-3.52 - 10.91	1.00
	0.9	-3.31***	-4.67 - -1.95	0.00	-10.09	-25.2 - 5.03	0.72	1.57	-12.41 - 15.55	1.00	-3.78	-24.73 - 17.16	1.00	-24.94*	-48.38 - -1.51	0.03	-4.76	-15.25 - 5.74	1.00
Electronic Health Information Exchange	0.1	-0.29	-0.71 - 0.13	0.63	0.12	-1.57 - 1.81	1.00	0.06	-2.01 - 2.14	1.00	0.01	-3.52 - 3.55	1.00	-0.99	-5.47 - 3.5	1.00	-0.58	-2.96 - 1.79	1.00
	0.5	-0.11	-0.45 - 0.22	1.00	1.29	-1.66 - 4.25	1.00	-0.68	-3.84 - 2.48	1.00	0.78	-4.62 - 6.17	1.00	0.50	-5.03 - 6.02	1.00	1.61	-0.65 - 3.86	0.53
	0.9	-0.04	-0.44 - 0.36	1.00	5.15*	0.16 - 10.13	0.04	1.15	-3.92 - 6.23	1.00	4.48	-2.18 - 11.14	0.70	0.91	-8.97 - 10.79	1.00	0.50	-2.86 - 3.86	1.00
:MEDITECH	0.1	0.05	-0.5 - 0.6	1.00	-0.18	-2.8 - 2.44	1.00	-0.11	-2.73 - 2.52	1.00	0.18	-4.49 - 4.85	1.00	0.05	-6.55 - 6.65	1.00	-0.47	-3.75 - 2.82	1.00
	0.5	0.27	-0.19 - 0.72	1.00	1.90	-2.29 - 6.08	1.00	-0.82	-5.17 - 3.53	1.00	-1.49	-8.82 - 5.83	1.00	-1.77	-9.41 - 5.88	1.00	-3.90**	-6.98 - -0.82	0.00
	0.9	-0.26	-0.78 - 0.26	1.00	1.65	-5.94 - 9.24	1.00	0.01	-7.31 - 7.33	1.00	-3.01	-11.97 - 5.95	1.00	-8.03	-21.67 - 5.61	1.00	-4.36	-8.93 - 0.21	0.08
:Epic	0.1	0.35	-0.22 - 0.92	1.00	-0.23	-2.54 - 2.09	1.00	-0.01	-2.7 - 2.67	1.00	0.85	-4.1 - 5.81	1.00	0.75	-5.01 - 6.51	1.00	1.43	-1.89 - 4.75	1.00
	0.5	0.25	-0.21 - 0.72	1.00	-3.42	-7.37 - 0.53	0.16	0.70	-3.62 - 5.01	1.00	0.87	-6.24 - 7.99	1.00	-1.36	-8.71 - 5.99	1.00	-1.54	-4.71 - 1.62	1.00
	0.9	-0.08	-0.64 - 0.49	1.00	-3.32	-10.18 - 3.55	1.00	-4.65	-11.72 - 2.42	0.77	-4.33	-13.33 - 4.67	1.00	-4.50	-16.7 - 7.7	1.00	0.08	-4.51 - 4.66	1.00
:Cerner	0.1	0.13	-0.41 - 0.67	1.00	-0.17	-2.46 - 2.13	1.00	-0.12	-2.74 - 2.49	1.00	0.59	-4.19 - 5.36	1.00	0.89	-4.69 - 6.46	1.00	-1.32	-4.49 - 1.85	1.00
	0.5	-0.17	-0.64 - 0.29	1.00	-0.90	-4.78 - 2.98	1.00	-0.12	-4.35 - 4.1	1.00	1.04	-5.9 - 7.97	1.00	-0.44	-7.53 - 6.65	1.00	-3.89**	-7.03 - -0.76	0.00
	0.9	-0.28	-0.84 - 0.28	1.00	-5.05	-11.59 - 1.48	0.34	-4.59	-11.07 - 1.9	0.55	0.38	-8.21 - 8.98	1.00	0.22	-12.02 - 12.46	1.00	-3.29	-7.91 - 1.33	0.53
:McKesson	0.1	0.37	-0.4 - 1.14	1.00	-0.18	-3.47 - 3.11	1.00	-0.03	-3.17 - 3.1	1.00	0.20	-5.43 - 5.83	1.00	-0.74	-7.91 - 6.44	1.00	0.82	-3.98 - 5.63	1.00
	0.5	-0.20	-0.78 - 0.39	1.00	-0.72	-5.49 - 4.05	1.00	-1.24	-6.34 - 3.86	1.00	-3.88	-12.38 - 4.62	1.00	-1.69	-10.73 - 7.34	1.00	-3.07	-7 - 0.85	0.31
	0.9	-0.29	-0.93 - 0.36	1.00	-2.38	-10.24 - 5.48	1.00	-4.71	-13.3 - 3.88	1.00	1.27	-9.62 - 12.15	1.00	1.39	-14.5 - 17.29	1.00	-5.84	-11.71 - 0.03	0.05
MEDITECH	0.1	0.24	-1.96 - 2.43	1.00	0.34	-20.51 - 21.2	1.00	-0.13	-14.72 - 14.46	1.00	-5.90	-31.39 - 19.58	1.00	-2.41	-69.85 - 65.03	1.00	2.59	-10.52 - 15.71	1.00
	0.5	-0.91	-2.81 - 0.99	1.00	-14.13	-44.71 - 16.46	1.00	-3.22	-27.53 - 21.08	1.00	10.76	-33.08 - 54.61	1.00	23.36	-52.8 - 99.52	1.00	18.85***	6.00 - 31.70	0.00
	0.9	3.50***	1.49 - 5.51	0.00	-20.09	-89.77 - 49.6	1.00	-34.34	-75.39 - 6.7	0.21	39.52	-13.92 - 92.96	0.44	-70.88	-190.32 - 48.56	1.00	-0.99	-19.86 - 17.88	1.00
:Num Beds 100-199	0.1	-0.02	-2.7 - 2.66	1.00	-0.27	-21.46 - 20.92	1.00	0.01	-15.17 - 15.2	1.00	2.69	-27.98 - 33.35	1.00	1.89	-68.47 - 72.25	1.00	-4.05	-20.39 - 12.29	1.00
	0.5	0.03	-2.3 - 2.35	1.00	-4.54	-37.23 - 28.15	1.00	-15.12	-41.97 - 11.72	1.00	-14.29	-62.77 - 34.19	1.00	-10.39	-89.57 - 68.8	1.00	-17.71*	-33.39 - -2.03	0.01
	0.9	-6.32***	-8.98 - -3.66	0.00	-10.73	-83.73 - 62.26	1.00	3.89	-40.27 - 48.04	1.00	-85.03***	-142.6 - -27.46	0.00	88.37	-40.18 - 216.91	0.63	10.48	-11.05 - 32.01	1.00
:Num Beds 200-299	0.1	2.16	-1.1 - 5.42	0.75	1.45	-20.5 - 23.41	1.00	-5.76	-23.44 - 11.91	1.00	-8.44	-42.88 - 26.01	1.00	-2.49	-73.5 - 68.51	1.00	8.89	-13.33 - 31.1	1.00
	0.5	1.89	-1.13 - 4.91	0.96	15.33	-19.25 - 49.91	1.00	-5.48	-35.92 - 24.96	1.00	-39.80	-92.93 - 13.34	0.41	-7.34	-88.24 - 73.55	1.00	-3.66	-24.12 - 16.8	1.00
	0.9	-1.39	-5 - 2.21	1.00	11.60	-63.67 - 86.88	1.00	55.27*	6.33 - 104.20	0.01	-41.89	-108.19 - 24.41	0.92	100.28	-28.17 - 228.74	0.32	31.85*	1.97 - 61.74	0.03
:Num Beds 300-399	0.1	2.06	-2.64 - 6.76	1.00	-6.14	-30.22 - 17.94	1.00	-22.47*	-42.78 - -2.15	0.02	9.50	-31.75 - 50.74	1.00	26.34	-48.71 - 101.39	1.00	3.16	-23.92 - 30.24	1.00

©2020 Murphy ZR et al. *JAMA Network Open*.

	0.5	1.94	-2.01 - 5.88	1.00	12.51	-26.2 - 51.22	1.00	6.95	-29.59 - 43.48	1.00	-64.37*	-126.09 - -2.65	0.03	29.58	-57.34 - 116.5	1.00	-4.50	-31.16 - 22.16	1.00
	0.9	-1.46	-6.31 - 3.39	1.00	31.17	-49.16 - 111.51	1.00	83.90***	25.43 - 142.36	0.00	-73.11	-150.55 - 4.34	0.08	132.20	-7.62 - 272.03	0.08	32.73	-11.68 - 77.14	0.44
:Num Beds 400-499	0.1	3.46	-6.07 - 13	1.00	10.20	-20.98 - 41.38	1.00	-16.19	-45.93 - 13.55	1.00	2.73	-53.58 - 59.04	1.00	35.45	-49.96 - 120.86	1.00	-5.88	-50.11 - 38.34	1.00
	0.5	2.76	-3.54 - 9.05	1.00	5.77	-45.47 - 57.01	1.00	31.50	-21.9 - 84.9	1.00	3.47	-83.26 - 90.2	1.00	13.09	-91.8 - 117.98	1.00	-6.69	-48.94 - 35.57	1.00
	0.9	-2.67	-9.79 - 4.46	1.00	1.77	-89.74 - 93.29	1.00	46.50	-37.97 - 130.98	1.00	-0.25	-120.69 - 120.19	1.00	55.09	-117.55 - 227.74	1.00	6.59	-62.04 - 75.23	1.00
:Num Beds 500+	0.1	-0.09	-6.64 - 6.46	1.00	4.46	-25.43 - 34.35	1.00	-3.32	-32.76 - 26.12	1.00	5.15	-53.32 - 63.63	1.00	-12.56	-97.35 - 72.24	1.00	32.77	-10.25 - 75.79	0.37
	0.5	1.40	-4.34 - 7.13	1.00	12.47	-35.52 - 60.47	1.00	-9.65	-58.81 - 39.51	1.00	-24.19	-104.35 - 55.96	1.00	-28.33	-128.89 - 72.23	1.00	9.89	-28.63 - 48.41	1.00
	0.9	0.32	-6.91 - 7.55	1.00	-2.33	-101.49 - 96.82	1.00	50.23	-29.76 - 130.22	0.94	-36.24	-139.11 - 66.64	1.00	32.08	-122.11 - 186.27	1.00	14.61	-47.75 - 76.97	1.00
Epic	0.1	0.88	-2.57 - 4.33	1.00	0.02	-25.66 - 25.69	1.00	-0.34	-20.25 - 19.57	1.00	0.12	-33.07 - 33.31	1.00	-4.46	-84.13 - 75.21	1.00	-3.01	-22.72 - 16.69	1.00
	0.5	-0.54	-3.39 - 2.31	1.00	-4.62	-41.1 - 31.86	1.00	27.47	-5.12 - 60.05	0.20	34.19	-20.03 - 88.42	0.92	35.78	-64.81 - 136.38	1.00	5.51	-13.76 - 24.78	1.00
	0.9	-0.69	-4.2 - 2.82	1.00	-12.34	-93.47 - 68.79	1.00	31.69	-21.16 - 84.53	1.00	10.65	-63.37 - 84.67	1.00	130.95*	17.96 - 243.95	0.01	-21.10	-51.67 - 9.48	0.62
:Num Beds 100-199	0.1	0.31	-2.67 - 3.28	1.00	-0.09	-24.94 - 24.75	1.00	-0.37	-18.1 - 17.36	1.00	4.04	-28.37 - 36.44	1.00	6.08	-73.54 - 85.71	1.00	11.46	-6.62 - 29.54	0.91
	0.5	0.97	-1.62 - 3.55	1.00	2.03	-32.91 - 36.96	1.00	-27.43	-57.41 - 2.55	0.11	-23.98	-74.18 - 26.22	1.00	-23.61	-121.93 - 74.72	1.00	-9.47	-26.92 - 7.98	1.00
	0.9	-2.23	-5.03 - 0.57	0.28	-18.97	-98.01 - 60.06	1.00	-24.16	-74.26 - 25.94	1.00	-33.37	-94.05 - 27.3	1.00	-85.70	-184.35 - 12.94	0.16	6.67	-17.24 - 30.57	1.00
:Num Beds 200-299	0.1	1.07	-2.31 - 4.44	1.00	0.65	-24.19 - 25.48	1.00	0.90	-18.3 - 20.09	1.00	13.23	-22.09 - 48.54	1.00	3.03	-76.76 - 82.83	1.00	3.79	-18.37 - 25.95	1.00
	0.5	1.67	-1.46 - 4.79	1.00	1.32	-35.06 - 37.7	1.00	-29.11	-61.88 - 3.65	0.14	-49.55	-103.79 - 4.7	0.11	-34.66	-134.22 - 64.89	1.00	-4.74	-25.84 - 16.36	1.00
	0.9	1.53	-2.19 - 5.25	1.00	-6.42	-87.27 - 74.42	1.00	4.69	-48.18 - 57.57	1.00	-15.52	-84.11 - 53.07	1.00	-134.88**	-236.51 - -33.25	0.00	22.29	-8.75 - 53.33	0.51
:Num Beds 300-399	0.1	1.80	-2.61 - 6.22	1.00	-13.43	-39.64 - 12.78	1.00	1.08	-20.94 - 23.1	1.00	21.19	-20.37 - 62.76	1.00	43.85	-39.96 - 127.67	1.00	11.71	-14.25 - 37.67	1.00
	0.5	1.92	-1.88 - 5.72	1.00	1.10	-38.28 - 40.48	1.00	-17.15	-54.33 - 20.03	1.00	-46.79	-107.34 - 13.77	0.34	-8.16	-111.5 - 95.19	1.00	-12.00	-37.71 - 13.71	1.00
	0.9	2.77	-1.84 - 7.38	1.00	13.06	-73.25 - 99.38	1.00	-1.19	-60.12 - 57.74	1.00	-11.94	-84.74 - 60.86	1.00	-90.41	-202.36 - 21.53	0.26	32.26	-7.7 - 72.23	0.26
:Num Beds 400-499	0.1	2.71	-4.26 - 9.67	1.00	-0.08	-28.7 - 28.53	1.00	-23.14	-46.79 - 0.52	0.06	-35.83	-78.74 - 7.08	0.21	5.98	-81 - 92.96	1.00	1.94	-32.31 - 36.2	1.00
	0.5	3.79	-1.14 - 8.71	0.35	-2.49	-47.43 - 42.45	1.00	-27.54	-72.22 - 17.14	1.00	-18.66	-90.49 - 53.18	1.00	-35.74	-145.63 - 74.15	1.00	-2.83	-35.92 - 30.25	1.00
	0.9	4.52	-0.42 - 9.46	0.11	-6.92	-91.2 - 77.36	1.00	-7.44	-78.45 - 63.57	1.00	56.37	-44.33 - 157.07	1.00	-112.76	-236.5 - 10.98	0.11	19.68	-23.17 - 62.53	1.00
:Num Beds 500+	0.1	0.01	-4.13 - 4.15	1.00	11.54	-14.91 - 37.99	1.00	9.44	-12.74 - 31.63	1.00	-1.54	-45.29 - 42.21	1.00	8.84	-75.94 - 93.63	1.00	17.58	-11.9 - 47.06	1.00
	0.5	2.38	-1.56 - 6.32	1.00	14.68	-25.21 - 54.57	1.00	-32.18	-70.01 - 5.66	0.19	-35.99	-97.23 - 25.25	1.00	-23.55	-127.51 - 80.4	1.00	2.81	-23.68 - 29.31	1.00
	0.9	-0.05	-5.13 - 5.02	1.00	4.73	-84.06 - 93.53	1.00	-24.57	-87.29 - 38.16	1.00	-10.83	-82.69 - 61.03	1.00	-89.65	-205.31 - 26.01	0.33	27.88	-10.76 - 66.51	0.50
Cerner	0.1	-0.63	-3.19 - 1.93	1.00	0.13	-24.66 - 24.92	1.00	-0.39	-16.55 - 15.77	1.00	-3.96	-34.3 - 26.37	1.00	-6.49	-90.23 - 77.25	1.00	-2.98	-17.78 - 11.82	1.00
	0.5	-3.06***	-5.19 - -0.93	0.00	-6.54	-42.66 - 29.57	1.00	13.00	-15.04 - 41.05	1.00	30.03	-18.57 - 78.63	1.00	8.72	-92.01 - 109.44	1.00	18.25**	3.85 - 32.65	0.00
	0.9	-1.19	-3.52 - 1.15	1.00	35.78	-36.54 - 108.1	1.00	1.34	-44.16 - 46.85	1.00	-11.34	-71.37 - 48.69	1.00	-65.59	-253.82 - 122.64	1.00	0.98	-19.17 - 21.13	1.00

©2020 Murphy ZR et al. *JAMA Network Open*.

:Num Beds 100-199	0.1	0.90	-2.21 - 4	1.00	-0.25	-26.54 - 26.04	1.00	-0.12	-17.57 - 17.33	1.00	1.03	-33.92 - 35.99	1.00	6.30	-80.08 - 92.68	1.00	10.13	-8.28 - 28.54	1.00
	0.5	2.40	-0.25 - 5.04	0.12	3.70	-34.3 - 41.69	1.00	-8.36	-38.95 - 22.24	1.00	-37.77	-90.91 - 15.38	0.54	0.23	-102.22 - 102.69	1.00	-22.31**	-40.18 - -4.43	0.00
	0.9	-2.00	-4.95 - 0.94	0.66	-56.51	-130.74 - 17.72	0.37	-22.40	-71.44 - 26.64	1.00	-19.67	-84.72 - 45.38	1.00	116.14	-73.16 - 305.44	1.00	-7.31	-32.15 - 17.53	1.00
:Num Beds 200-299	0.1	0.23	-3.24 - 3.69	1.00	0.47	-26.31 - 27.24	1.00	-6.00	-24.93 - 12.93	1.00	7.51	-30.66 - 45.68	1.00	0.82	-85.13 - 86.77	1.00	9.59	-13.63 - 32.81	1.00
	0.5	2.51	-0.72 - 5.73	0.33	11.30	-28.16 - 50.75	1.00	-10.85	-44.47 - 22.78	1.00	-47.22	-104.52 - 10.07	0.23	-19.48	-123.44 - 84.49	1.00	-14.30	-36.06 - 7.47	0.78
	0.9	2.72	-0.88 - 6.31	0.39	-44.64	-121.36 - 32.09	1.00	12.69	-40.52 - 65.89	1.00	-10.06	-83.42 - 63.3	1.00	20.26	-170.23 - 210.75	1.00	-2.49	-34.31 - 29.32	1.00
:Num Beds 300-399	0.1	1.78	-2.68 - 6.24	1.00	-16.92	-44.58 - 10.74	1.00	-9.97	-30.62 - 10.69	1.00	-3.59	-45.94 - 38.75	1.00	22.22	-66.25 - 110.69	1.00	10.65	-15.05 - 36.36	1.00
	0.5	1.63	-2.14 - 5.4	1.00	4.43	-37.32 - 46.17	1.00	-11.93	-49.13 - 25.26	1.00	-80.14**	-142.41 - -17.88	0.00	0.40	-106.17 - 106.98	1.00	-15.26	-40.8 - 10.27	1.00
	0.9	3.12	-1.56 - 7.8	0.73	-44.28	-126.62 - 38.06	1.00	11.68	-46.73 - 70.08	1.00	-43.12	-116.79 - 30.55	1.00	76.36	-117.74 - 270.45	1.00	2.69	-36.07 - 41.45	1.00
:Num Beds 400-499	0.1	3.30	-4.28 - 10.89	1.00	-3.79	-34.16 - 26.59	1.00	-29.57**	-53.93 - -5.2	0.01	-60.70**	-106.69 - -14.7	0.00	-2.69	-97.06 - 91.67	1.00	-14.40	-51.98 - 23.18	1.00
	0.5	4.81	-0.42 - 10.04	0.10	11.57	-37.15 - 60.3	1.00	-30.17	-77.31 - 16.96	0.87	-31.42	-108.32 - 45.48	1.00	-26.70	-141.91 - 88.52	1.00	-21.83	-57.07 - 13.41	0.99
	0.9	4.98	-0.3 - 10.25	0.08	-27.76	-109.67 - 54.16	1.00	16.91	-55.17 - 89	1.00	25.86	-85.99 - 137.7	1.00	66.64	-137.02 - 270.31	1.00	10.38	-35.7 - 56.45	1.00
:Num Beds 500+	0.1	0.19	-4.17 - 4.55	1.00	-3.31	-31.25 - 24.63	1.00	4.78	-16.9 - 26.46	1.00	-6.47	-52.94 - 40	1.00	8.90	-80.27 - 98.06	1.00	8.90	-23.07 - 40.87	1.00
	0.5	2.54	-1.53 - 6.62	0.97	1.17	-42.04 - 44.38	1.00	-18.54	-57.64 - 20.55	1.00	-49.85	-114.73 - 15.03	0.35	-5.47	-114.01 - 103.07	1.00	-13.16	-40.56 - 14.24	1.00
	0.9	0.66	-4.52 - 5.84	1.00	-46.43	-132.84 - 39.98	1.00	-13.29	-77.17 - 50.58	1.00	28.93	-47.7 - 105.56	1.00	87.37	-109.58 - 284.32	1.00	9.24	-31.24 - 49.71	1.00
McKesson	0.1	1.14	-1.81 - 4.09	1.00	-0.16	-23.53 - 23.22	1.00	0.09	-18.39 - 18.58	1.00	4.29	-33.77 - 42.35	1.00	-12.98	-93.84 - 67.89	1.00	-3.74	-20.3 - 12.83	1.00
	0.5	-0.78	-3.06 - 1.5	1.00	-8.73	-46.82 - 29.35	1.00	8.37	-21.46 - 38.21	1.00	22.78	-30.84 - 76.41	1.00	-54.64	-144.95 - 35.66	1.00	-3.77	-19.14 - 11.59	1.00
	0.9	-0.14	-2.96 - 2.69	1.00	-49.35	-140.41 - 41.72	1.00	-12.31	-55.67 - 31.05	1.00	33.70	-27.61 - 95.02	1.00	-38.84	-185.5 - 107.83	1.00	-17.51	-41.8 - 6.79	0.50
:Num Beds 100-199	0.1	0.62	-2.92 - 4.16	1.00	0.91	-24.08 - 25.89	1.00	-0.07	-19.85 - 19.71	1.00	-7.89	-51.57 - 35.8	1.00	13.52	-71.95 - 98.99	1.00	4.65	-15.99 - 25.29	1.00
	0.5	1.85	-1.06 - 4.75	0.89	12.48	-28.11 - 53.07	1.00	-13.27	-46.71 - 20.16	1.00	-21.55	-80.71 - 37.61	1.00	91.01	-3.09 - 185.11	0.07	1.76	-17.74 - 21.27	1.00
	0.9	3.44*	0.04 - 6.84	0.05	54.65	-35.37 - 144.67	1.00	14.20	-35.74 - 64.14	1.00	-37.00	-107.94 - 33.94	1.00	76.02	-71.96 - 224	1.00	27.01	-1.48 - 55.5	0.08
:Num Beds 200-299	0.1	-2.09	-5.94 - 1.77	1.00	1.26	-23.87 - 26.39	1.00	11.11	-8.96 - 31.17	1.00	-16.83	-62.23 - 28.58	1.00	32.62	-52.6 - 117.84	1.00	-0.64	-26.13 - 24.85	1.00
	0.5	0.55	-2.95 - 4.05	1.00	15.92	-26.02 - 57.86	1.00	-2.91	-39.31 - 33.5	1.00	-26.49	-89.16 - 36.17	1.00	81.33	-13.22 - 175.88	0.17	2.94	-20.87 - 26.75	1.00
	0.9	-0.73	-4.76 - 3.29	1.00	75.21	-16.92 - 167.33	0.24	20.14	-36.09 - 76.38	1.00	-8.68	-87.91 - 70.56	1.00	86.15	-61.9 - 234.2	1.00	29.98	-8.1 - 68.05	0.30
:Num Beds 300-399	0.1	1.00	-4.7 - 6.7	1.00	-4.67	-33.44 - 24.11	1.00	-2.31	-26.17 - 21.55	1.00	20.35	-31.69 - 72.39	1.00	39.18	-51.47 - 129.84	1.00	-3.10	-36.13 - 29.92	1.00
	0.5	2.32	-2.35 - 6.98	1.00	30.83	-16 - 77.65	0.77	19.64	-23.89 - 63.16	1.00	-28.57	-101.24 - 44.1	1.00	71.57	-30.84 - 173.99	0.58	1.03	-30.43 - 32.48	1.00
	0.9	3.47	-2.44 - 9.38	1.00	80.47	-21.48 - 182.42	0.30	74.37*	3.52 - 145.21	0.03	-38.29	-134.74 - 58.15	1.00	162.11*	8.47 - 315.75	0.03	56.82*	6.56 - 107.08	0.01
:Num Beds 400-499	0.1	1.29	-6.72 - 9.31	1.00	2.39	-27.54 - 32.32	1.00	-31.34**	-58.27 - -4.41	0.01	-61.81*	-116.98 - -6.63	0.02	29.52	-63.99 - 123.03	1.00	0.70	-36.92 - 38.31	1.00
	0.5	0.13	-5.55 - 5.81	1.00	20.62	-31.59 - 72.83	1.00	-9.35	-60.21 - 41.51	1.00	-52.56	-136.26 - 31.15	0.94	65.02	-44.58 - 174.62	1.00	9.33	-28.81 - 47.47	1.00
	0.9	3.27	-2.52 - 9.07	1.00	62.04	-36.7 - 160.78	0.94	32.15	-53.46 - 117.76	1.00	13.79	-97.14 - 124.72	1.00	91.08	-81.72 - 263.88	1.00	27.29	-23.83 - 78.41	1.00

©2020 Murphy ZR et al. *JAMA Network Open*.

:Num Beds 500+	0.1	1.17	-6.19 - 8.53	1.00	-2.51	-35.83 - 30.8	1.00	8.87	-21.59 - 39.32	1.00	-44.53	-111.59 - 22.54	0.74	10.89	-87.28 - 109.06	1.00	20.17	-29.98 - 70.33	1.00
	0.5	2.33	-3.69 - 8.34	1.00	2.03	-51.63 - 55.69	1.00	18.98	-34.12 - 72.08	1.00	-49.80	-136.39 - 36.79	1.00	88.51	-23.19 - 200.21	0.29	14.27	-26.11 - 54.65	1.00
	0.9	-1.11	-8.81 - 6.59	1.00	28.59	-87.53 - 144.7	1.00	27.43	-63.81 - 118.66	1.00	-28.09	-146.89 - 90.71	1.00	153.76	-25.43 - 332.95	0.17	15.99	-50.26 - 82.24	1.00
Years of MU attestation	0.1	0.01	-0.45 - 0.47	1.00	0.03	-2 - 2.05	1.00	-0.01	-2.18 - 2.16	1.00	-0.09	-4.72 - 4.54	1.00	0.00	-4.7 - 4.7	1.00	-0.46	-3.34 - 2.43	1.00
	0.5	0.03	-0.38 - 0.44	1.00	-2.36	-5.76 - 1.04	0.60	0.99	-2.7 - 4.68	1.00	-5.18	-11.36 - 1	0.20	5.25	-1.12 - 11.62	0.23	-0.91	-3.69 - 1.86	1.00
	0.9	-0.26	-0.78 - 0.25	1.00	-2.10	-7.78 - 3.58	1.00	1.33	-4.64 - 7.31	1.00	-7.97*	-15.89 - -0.05	0.05	-0.83	-10.32 - 8.65	1.00	3.55	-0.76 - 7.86	0.23
Total Inpatient Revenue	0.1	0.05	-0.06 - 0.17	1.00	0.26	-0.04 - 0.57	0.16	0.23	-0.07 - 0.54	0.36	0.23	-0.5 - 0.96	1.00	0.24	-0.55 - 1.03	1.00	0.38	-0.18 - 0.93	0.67
	0.5	0.03	-0.04 - 0.09	1.00	0.07	-0.38 - 0.53	1.00	-0.16	-0.68 - 0.36	1.00	-0.06	-0.9 - 0.77	1.00	-0.13	-0.94 - 0.67	1.00	0.11	-0.33 - 0.55	1.00
	0.9	-0.02	-0.09 - 0.05	1.00	-0.03	-0.89 - 0.83	1.00	-0.36	-1.32 - 0.6	1.00	-0.91	-2.03 - 0.21	0.25	-0.59	-1.96 - 0.77	1.00	-0.30	-1.19 - 0.59	1.00
Discharges Medicare Percent	0.1	6.35**	1.46 - 11.23	0.00	-2.01	-23.5 - 19.48	1.00	0.69	-20.55 - 21.92	1.00	2.87	-40.91 - 46.65	1.00	4.04	-41.73 - 49.81	1.00	48.20***	19.27 - 77.13	0.00
	0.5	2.16	-1.82 - 6.13	1.00	-2.62	-36.08 - 30.84	1.00	-16.15	-53.3 - 21	1.00	-3.70	-66.28 - 58.88	1.00	65.84*	2.72 - 128.97	0.03	71.10***	44.22 - 97.98	0.00
	0.9	-0.89	-5.71 - 3.92	1.00	-3.29	-61.94 - 55.36	1.00	-40.60	-104.01 - 22.81	0.87	-107.25**	-193.11 - -21.39	0.00	86.52	-23.09 - 196.13	0.30	87.95***	47.31 - 128.59	0.00
Discharges Medicaid Percent	0.1	-2.10	-6.17 - 1.96	1.00	1.29	-20.88 - 23.46	1.00	-0.14	-20.8 - 20.52	1.00	-0.21	-44.17 - 43.74	1.00	23.16	-23.22 - 69.54	1.00	-14.25	-41.92 - 13.42	1.00
	0.5	-4.92**	-8.79 - -1.05	0.00	29.19	-5.29 - 63.66	0.19	-0.04	-36.33 - 36.25	1.00	-13.85	-75.4 - 47.69	1.00	72.86**	11.06 - 134.66	0.01	-27.45*	-53.53 - -1.36	0.03
	0.9	-2.46	-6.66 - 1.73	1.00	4.71	-57.91 - 67.33	1.00	-34.44	-93.46 - 24.59	1.00	89.33**	17.69 - 160.98	0.00	109.04*	12.07 - 206.01	0.02	22.71	-15.72 - 61.13	1.00
Teaching Hospital Yes	0.1	0.44	-0.5 - 1.38	1.00	0.05	-3.88 - 3.97	1.00	0.10	-3.94 - 4.14	1.00	0.41	-7.98 - 8.81	1.00	2.86	-6.28 - 12	1.00	-1.52	-7.14 - 4.1	1.00
	0.5	0.08	-0.79 - 0.95	1.00	1.06	-5.45 - 7.58	1.00	0.94	-6.32 - 8.2	1.00	10.75	-1.12 - 22.62	0.12	-0.01	-11.99 - 11.97	1.00	-4.85	-10.7 - 1	0.22
	0.9	0.04	-0.94 - 1.02	1.00	3.75	-7.78 - 15.28	1.00	2.62	-9.63 - 14.86	1.00	7.48	-7.58 - 22.54	1.00	-20.47**	-37.78 - -3.16	0.01	-15.38***	-24.16 - -6.6	0.00
Vendor Count	0.1	0.04	-0.27 - 0.35	1.00	0.02	-1.4 - 1.45	1.00	-0.03	-1.34 - 1.29	1.00	-1.06	-4.03 - 1.92	1.00	-0.83	-4 - 2.35	1.00	0.86	-1 - 2.72	1.00
	0.5	-0.09	-0.36 - 0.18	1.00	1.54	-0.83 - 3.91	0.81	-0.29	-2.74 - 2.16	1.00	0.35	-3.9 - 4.59	1.00	-3.05	-7.43 - 1.33	0.59	-0.43	-2.26 - 1.4	1.00
	0.9	-0.30	-0.62 - 0.02	0.09	1.25	-2.67 - 5.17	1.00	1.19	-2.93 - 5.31	1.00	0.30	-5.36 - 5.96	1.00	-5.45	-12.41 - 1.51	0.32	-3.59**	-6.44 - -0.75	0.00
Criteria Coverage Percent	0.1	0.01	-0.02 - 0.03	1.00	0.00	-0.1 - 0.1	1.00	-0.01	-0.12 - 0.1	1.00	-0.03	-0.23 - 0.18	1.00	0.03	-0.22 - 0.29	1.00	-0.06	-0.2 - 0.09	1.00
	0.5	0.01	-0.02 - 0.03	1.00	0.08	-0.1 - 0.25	1.00	-0.06	-0.25 - 0.13	1.00	-0.05	-0.35 - 0.25	1.00	0.25	-0.08 - 0.58	0.39	-0.16*	-0.3 - -0.02	0.02
	0.9	0.00	-0.03 - 0.02	1.00	0.07	-0.23 - 0.37	1.00	0.05	-0.25 - 0.35	1.00	0.00	-0.37 - 0.37	1.00	0.33	-0.14 - 0.81	0.55	-0.01	-0.22 - 0.19	1.00
Magnet Yes	0.1	-0.92	-2.35 - 0.5	0.83	-0.08	-5.06 - 4.9	1.00	0.11	-5.06 - 5.28	1.00	12.73*	1.68 - 23.78	0.01	-3.82	-14.88 - 7.24	1.00	0.46	-7.75 - 8.68	1.00
	0.5	0.10	-1.12 - 1.32	1.00	1.73	-6.78 - 10.24	1.00	4.31	-5.44 - 14.06	1.00	13.24	-2.21 - 28.69	0.18	-18.87**	-34.01 - -3.73	0.00	-1.62	-9.82 - 6.57	1.00
	0.9	-0.20	-1.63 - 1.24	1.00	-1.14	-15.4 - 13.13	1.00	-11.40	-27.13 - 4.32	0.48	5.48	-15.05 - 26.01	1.00	-23.65	-48.6 - 1.3	0.08	1.42	-11.22 - 14.07	1.00
CMI	0.1	0.59	-1.87 - 3.06	1.00	1.43	-7.85 - 10.72	1.00	1.33	-7.66 - 10.31	1.00	1.73	-18.19 - 21.66	1.00	14.50	-5.07 - 34.07	0.43	9.26	-5.19 - 23.7	0.86
	0.5	-0.70	-2.42 - 1.01	1.00	9.86	-5.1 - 24.81	0.76	15.25	-1.15 - 31.65	0.09	-6.12	-34.55 - 22.3	1.00	38.85***	10.69 - 67.02	0.00	18.42***	6.86 - 29.97	0.00

©2020 Murphy ZR et al. *JAMA Network Open*.

	0.9	-3.87***	-5.98 - -1.76	0.00	-1.70	-27.18 - 23.78	1.00	7.55	-18.47 - 33.57	1.00	-29.32	-66.09 - 7.46	0.28	48.91*	3.50 - 94.33	0.02	30.60***	15.26 - 45.94	0.00
const	0.1	91.41***	89.09 - 93.72	0.00	0.48	-17.29 - 18.25	1.00	1.09	-11.61 - 13.79	1.00	7.14	-17.88 - 32.16	1.00	-2.17	-56.08 - 51.75	1.00	19.39**	4.91 - 33.88	0.00
	0.5	99.15***	97.19 - 101.10	0.00	15.26	-12.55 - 43.08	1.00	5.99	-16.24 - 28.23	1.00	59.90***	19.31 - 100.48	0.00	66.16*	3.44 - 128.87	0.03	66.27***	53.05 - 79.49	0.00
	0.9	106.44***	104.14 - 108.75	0.00	111.93***	48.13 - 175.73	0.00	100.93***	64.94 - 136.92	0.00	185.26***	136.98 - 233.54	0.00	273.02***	175.20 - 370.83	0.00	152.75***	133.55 - 171.96	0.00
Hospital Ownerhsip (ref Non-profit)																			
Government	0.1	0.05	-1.14 - 1.24	1.00	0.62	-4.75 - 5.98	1.00	0.15	-5.42 - 5.72	1.00	0.93	-10.55 - 12.41	1.00	3.48	-9.3 - 16.26	1.00	-0.14	-7.33 - 7.05	1.00
	0.5	0.86	-0.2 - 1.92	0.26	-0.19	-9.64 - 9.26	1.00	-4.87	-14.93 - 5.19	1.00	-8.05	-24.63 - 8.52	1.00	6.66	-10.38 - 23.7	1.00	2.57	-4.65 - 9.8	1.00
	0.9	1.08	-0.17 - 2.33	0.16	5.92	-10.44 - 22.29	1.00	-3.35	-20.1 - 13.4	1.00	11.90	-9.82 - 33.62	1.00	14.24	-14.81 - 43.3	1.00	2.39	-8.79 - 13.57	1.00
Proprietary	0.1	1.35**	0.21 - 2.49	0.01	-0.27	-5.45 - 4.91	1.00	-0.05	-4.97 - 4.86	1.00	-3.05	-13.57 - 7.47	1.00	-1.71	-13.32 - 9.9	1.00	-6.96*	-13.74 - -0.18	0.04
	0.5	1.64***	0.65 - 2.64	0.00	2.46	-5.63 - 10.56	1.00	0.76	-8.07 - 9.59	1.00	-6.61	-21.84 - 8.62	1.00	8.08	-7.4 - 23.57	1.00	-2.47	-9.18 - 4.24	1.00
	0.9	1.85***	0.68 - 3.01	0.00	18.77***	5.30 - 32.23	0.00	0.92	-13.82 - 15.66	1.00	10.83	-8.78 - 30.43	1.00	56.52***	31.46 - 81.59	0.00	4.58	-5.83 - 14.98	1.00
Number of Beds (ref 1-99)																			
100-199	0.1	3.51***	1.31 - 5.72	0.00	-0.14	-17.48 - 17.21	1.00	-0.14	-11.54 - 11.27	1.00	-0.83	-25.73 - 24.06	1.00	-1.85	-56.66 - 52.96	1.00	21.46***	7.95 - 34.98	0.00
	0.5	2.33**	0.49 - 4.17	0.00	22.25	-5.03 - 49.53	0.24	28.38**	7.43 - 49.32	0.00	12.80	-27.21 - 52.81	1.00	-15.00	-76.95 - 46.94	1.00	29.02***	16.59 - 41.44	0.00
	0.9	5.21***	3.01 - 7.41	0.00	18.60	-43.14 - 80.34	1.00	26.14	-7.13 - 59.42	0.31	9.75	-38.44 - 57.93	1.00	-83.37	-178.88 - 12.13	0.15	-16.59	-33.38 - 0.21	0.06
200-299	0.1	4.75***	2.12 - 7.37	0.00	-1.13	-18.72 - 16.46	1.00	5.99	-7.56 - 19.54	1.00	10.03	-18.36 - 38.41	1.00	7.95	-47.44 - 63.34	1.00	30.01***	11.71 - 48.30	0.00
	0.5	3.20**	0.75 - 5.65	0.00	24.68	-4.12 - 53.49	0.18	42.75***	18.49 - 67.00	0.00	31.84	-12.03 - 75.71	0.48	13.31	-50.02 - 76.64	1.00	20.59**	4.07 - 37.12	0.00
	0.9	2.78	-0.18 - 5.73	0.09	-8.65	-73 - 55.69	1.00	-5.27	-43.05 - 32.52	1.00	-11.76	-67.37 - 43.85	1.00	-57.33	-152.34 - 37.68	1.00	-24.28	-49.44 - 0.88	0.07
300-399	0.1	4.82**	0.90 - 8.73	0.01	16.53	-3.07 - 36.12	0.20	22.54***	6.17 - 38.92	0.00	4.75	-29.3 - 38.8	1.00	-4.89	-64.25 - 54.48	1.00	28.69**	5.78 - 51.60	0.00
	0.5	3.72*	0.50 - 6.93	0.01	22.77	-9.37 - 54.92	0.54	39.45**	10.02 - 68.88	0.00	59.08*	7.74 - 110.42	0.01	-11.94	-80.55 - 56.66	1.00	21.90*	0.06 - 43.74	0.05
	0.9	2.70	-1.56 - 6.96	0.90	-28.13	-97.56 - 41.29	1.00	-3.61	-49.21 - 41.99	1.00	8.53	-51.62 - 68.68	1.00	-106.66	-213.54 - 0.23	0.05	-36.36*	-71.23 - -1.49	0.03
400-499	0.1	2.70	-4.35 - 9.76	1.00	16.50	-6.62 - 39.63	0.52	46.26***	25.44 - 67.09	0.00	73.88***	33.51 - 114.26	0.00	27.41	-37.59 - 92.41	1.00	50.28***	18.09 - 82.47	0.00
	0.5	2.04	-2.64 - 6.73	1.00	25.40	-14.59 - 65.39	0.90	47.47**	7.31 - 87.63	0.01	29.50	-37.03 - 96.04	1.00	7.09	-73.02 - 87.21	1.00	21.87	-9.6 - 53.34	0.60
	0.9	0.11	-4.85 - 5.06	1.00	-24.66	-91.94 - 42.61	1.00	-11.61	-73.12 - 49.9	1.00	-46.49	-142.86 - 49.87	1.00	-93.15	-217.18 - 30.89	0.40	-39.01	-81.64 - 3.62	0.11
500+	0.1	6.05***	1.65 - 10.45	0.00	11.25	-9.18 - 31.68	1.00	14.08	-4.78 - 32.94	0.41	34.13	-8.03 - 76.29	0.26	32.61	-28.89 - 94.1	1.00	27.57*	0.24 - 54.91	0.05
	0.5	2.92	-0.8 - 6.64	0.31	18.39	-16.54 - 53.33	1.00	47.95***	14.78 - 81.11	0.00	38.09	-17.92 - 94.11	0.66	3.72	-69.28 - 76.71	1.00	18.72	-6.28 - 43.72	0.41
	0.9	3.61	-1.38 - 8.59	0.49	-27.52	-104.45 - 49.41	1.00	3.37	-54.56 - 61.31	1.00	-6.19	-73.99 - 61.62	1.00	-108.21	-218.26 - 1.84	0.06	-29.98	-69.91 - 9.95	0.40
Geographic Division (ref East North Central)																			
East South Central	0.1	0.94	-0.87 - 2.75	1.00	-0.19	-8.83 - 8.44	1.00	0.53	-7.84 - 8.91	1.00	-2.62	-19.16 - 13.91	1.00	10.30	-9.52 - 30.11	1.00	-9.78	-20.92 - 1.35	0.15

©2020 Murphy ZR et al. *JAMA Network Open*.

		0.5	0.29	-1.28 - 1.87	1.00	3.54	-10.4 - 17.47	1.00	3.99	-10.76 - 18.75	1.00	-9.07	-33.56 - 15.41	1.00	44.62***	18.58 - 70.67	0.00	-13.70**	-24.34 - -3.05	0.00
		0.9	-0.50	-2.29 - 1.29	1.00	-5.47	-30.4 - 19.46	1.00	-17.52	-40.93 - 5.89	0.41	4.11	-27.45 - 35.66	1.00	15.95	-26.52 - 58.42	1.00	-11.07	-26.64 - 4.5	0.54
	Mid Atlantic	0.1	-0.75	-2.32 - 0.83	1.00	0.13	-6.38 - 6.64	1.00	-0.18	-6.7 - 6.34	1.00	3.59	-10.4 - 17.58	1.00	8.84	-5.34 - 23.02	0.97	-4.42	-12.98 - 4.14	1.00
		0.5	0.87	-0.48 - 2.22	0.84	-0.54	-11.49 - 10.41	1.00	7.21	-4.8 - 19.22	1.00	5.13	-14.69 - 24.94	1.00	13.52	-6.18 - 33.22	0.64	-0.23	-9.32 - 8.86	1.00
		0.9	1.88**	0.31 - 3.46	0.01	16.47	-2.84 - 35.79	0.18	0.41	-19.18 - 19.99	1.00	21.15	-3.56 - 45.86	0.18	24.77	-9.71 - 59.26	0.51	9.68	-4.2 - 23.57	0.59
	Mountain	0.1	-2.21*	-4.2 - -0.23	0.02	-0.84	-9.21 - 7.53	1.00	-0.27	-8.94 - 8.4	1.00	0.78	-17 - 18.55	1.00	-3.55	-23.43 - 16.33	1.00	11.18	-0.01 - 22.37	0.05
		0.5	-0.58	-2.29 - 1.12	1.00	-11.07	-25.04 - 2.9	0.29	-0.36	-16.07 - 15.36	1.00	10.55	-14.59 - 35.68	1.00	-11.98	-38.89 - 14.93	1.00	13.34**	1.87 - 24.81	0.01
		0.9	-1.45	-3.38 - 0.47	0.39	-11.43	-35.93 - 13.07	1.00	12.09	-14.85 - 39.03	1.00	34.11*	0.93 - 67.29	0.04	-17.73	-61.09 - 25.63	1.00	5.64	-11.88 - 23.16	1.00
	New England	0.1	0.45	-1.84 - 2.75	1.00	0.32	-9.15 - 9.78	1.00	0.20	-9.61 - 10.01	1.00	11.12	-6.72 - 28.96	0.97	0.41	-20.23 - 21.05	1.00	1.36	-11.77 - 14.5	1.00
		0.5	0.46	-1.46 - 2.38	1.00	2.26	-13.12 - 17.64	1.00	9.36	-7.4 - 26.13	1.00	23.26	-3.78 - 50.3	0.17	-6.85	-35.16 - 21.45	1.00	6.74	-6.15 - 19.64	1.00
		0.9	-2.05	-4.38 - 0.28	0.15	5.15	-22.03 - 32.33	1.00	35.63**	8.47 - 62.79	0.00	58.89***	25.32 - 92.46	0.00	-45.42	-91.47 - 0.63	0.06	7.23	-11.83 - 26.3	1.00
	Pacific	0.1	-4.34***	-6.02 - -2.65	0.00	-0.35	-7.37 - 6.67	1.00	-0.47	-7.33 - 6.39	1.00	-2.66	-17.45 - 12.12	1.00	0.88	-15.18 - 16.94	1.00	9.10	-0.78 - 18.97	0.10
		0.5	-3.82***	-5.26 - -2.38	0.00	-1.77	-13.13 - 9.58	1.00	12.72*	0.09 - 25.35	0.05	2.89	-18.12 - 23.89	1.00	11.21	-10.28 - 32.69	1.00	8.16	-1.51 - 17.83	0.20
		0.9	-2.15***	-3.63 - -0.66	0.00	3.81	-15.77 - 23.39	1.00	14.86	-5.83 - 35.55	0.51	11.29	-15.38 - 37.97	1.00	-23.46	-57.18 - 10.26	0.60	16.35**	2.45 - 30.25	0.01
	South Atlantic	0.1	-1.27	-2.72 - 0.18	0.15	0.21	-5.86 - 6.28	1.00	0.25	-5.49 - 6	1.00	-0.05	-12.5 - 12.4	1.00	7.34	-6.36 - 21.04	1.00	-3.07	-11.64 - 5.5	1.00
		0.5	-0.81	-2.04 - 0.41	0.75	2.53	-7.42 - 12.48	1.00	7.00	-3.86 - 17.86	0.84	-0.70	-18.44 - 17.03	1.00	18.74*	0.39 - 37.08	0.04	-7.86	-16.11 - 0.38	0.08
		0.9	-1.66**	-3.03 - -0.29	0.01	18.72*	1.60 - 35.84	0.02	6.54	-11.39 - 24.48	1.00	-3.88	-26.65 - 18.89	1.00	25.39	-4.24 - 55.03	0.18	-8.71	-20.83 - 3.42	0.51
	West North Central	0.1	-3.94***	-5.65 - -2.22	0.00	-0.09	-8.21 - 8.04	1.00	0.00	-8.13 - 8.13	1.00	3.07	-13.04 - 19.18	1.00	-3.71	-20.79 - 13.38	1.00	-8.58	-18.58 - 1.41	0.17
		0.5	-3.07***	-4.56 - -1.59	0.00	1.18	-12.17 - 14.53	1.00	5.09	-9.28 - 19.45	1.00	10.19	-12.38 - 32.75	1.00	-15.67	-40.25 - 8.91	0.88	-6.76	-16.82 - 3.29	0.70
		0.9	-2.71***	-4.4 - -1.02	0.00	-3.51	-26.36 - 19.34	1.00	9.76	-13.43 - 32.94	1.00	22.88	-6.62 - 52.39	0.33	-53.21***	-91.92 - -14.5	0.00	-2.60	-16.86 - 11.66	1.00
	West South Central	0.1	1.78**	0.29 - 3.28	0.01	0.09	-6.57 - 6.75	1.00	0.23	-6.31 - 6.77	1.00	-0.22	-14.09 - 13.66	1.00	5.79	-9.12 - 20.71	1.00	-4.73	-13.82 - 4.36	1.00
		0.5	3.64***	2.33 - 4.95	0.00	7.93	-3.09 - 18.96	0.50	6.35	-5.73 - 18.42	1.00	-5.90	-25.75 - 13.95	1.00	21.97*	1.05 - 42.89	0.03	-6.32	-15.17 - 2.54	0.53
		0.9	4.98***	3.49 - 6.47	0.00	-1.13	-21.24 - 18.98	1.00	-0.19	-19.57 - 19.19	1.00	-7.70	-32.62 - 17.22	1.00	19.51	-13.86 - 52.89	1.00	0.39	-12.75 - 13.52	1.00
NCHS Code (ref Large central metro)																				
	Large fringe metro	0.1	-1.00	-2.26 - 0.25	0.28	-0.16	-5.39 - 5.07	1.00	-0.19	-5.48 - 5.09	1.00	-2.89	-14.39 - 8.61	1.00	-0.97	-12.78 - 10.84	1.00	-4.54	-11.94 - 2.85	1.00
		0.5	-1.19*	-2.33 - -0.05	0.03	-6.31	-14.75 - 2.12	0.41	-5.95	-15.46 - 3.55	0.95	-5.55	-21.26 - 10.16	1.00	-10.94	-26.45 - 4.56	0.55	-5.55	-13.19 - 2.1	0.48
		0.9	-3.11***	-4.42 - -1.8	0.00	-6.95	-21.31 - 7.4	1.00	6.96	-7.82 - 21.74	1.00	-29.28***	-48.94 - -9.61	0.00	-21.45	-45.51 - 2.61	0.13	-6.74	-18.14 - 4.66	1.00
	Medium metro	0.1	-1.50**	-2.78 - -0.22	0.01	0.17	-4.61 - 4.95	1.00	-0.22	-5.19 - 4.74	1.00	-3.27	-13.75 - 7.21	1.00	-3.29	-14.54 - 7.96	1.00	-7.82*	-15.05 - -0.59	0.02
		0.5	-2.11***	-3.21 - -1.01	0.00	-5.20	-13.31 - 2.91	0.87	-1.87	-11.02 - 7.29	1.00	-0.07	-15.19 - 15.06	1.00	-13.98	-28.85 - 0.88	0.09	-9.34**	-16.76 - -1.91	0.00

©2020 Murphy ZR et al. *JAMA Network Open*.

		0.9	-3.32***	-4.57 - -2.07	0.00	-11.36	-25.28 - 2.55	0.24	6.53	-8.54 - 21.6	1.00	-22.71**	-41.62 - -3.81	0.01	-15.35	-38.12 - 7.42	0.69	-9.58	-20.75 - 1.59	0.18
	Small metro	0.1	-3.68***	-5.19 - -2.18	0.00	0.35	-5.31 - 6.02	1.00	-0.28	-6.56 - 5.99	1.00	-2.05	-14.77 - 10.68	1.00	-1.77	-15.79 - 12.26	1.00	-6.13	-15.3 - 3.04	0.72
		0.5	-3.08***	-4.41 - -1.76	0.00	-0.55	-10.5 - 9.4	1.00	-1.19	-12.4 - 10.02	1.00	-16.97	-35.33 - 1.4	0.10	-20.52*	-38.97 - -2.07	0.02	-11.89**	-20.82 - -2.97	0.00
		0.9	-5.02***	-6.6 - -3.44	0.00	9.95	-6.87 - 26.78	1.00	0.33	-17.78 - 18.45	1.00	-39.85***	-62.6 - -17.1	0.00	-4.27	-33.62 - 25.07	1.00	-5.00	-18.23 - 8.23	1.00
	Micropolitan	0.1	-3.99***	-5.58 - -2.41	0.00	0.49	-7.53 - 8.52	1.00	-0.20	-7.64 - 7.24	1.00	-3.05	-19.45 - 13.34	1.00	-1.64	-20.6 - 17.32	1.00	-8.60	-18.08 - 0.88	0.11
		0.5	-4.08***	-5.49 - -2.68	0.00	-9.58	-22.77 - 3.62	0.48	-1.58	-14.65 - 11.5	1.00	3.52	-18.64 - 25.69	1.00	-18.49	-43.28 - 6.29	0.42	-13.19***	-22.65 - -3.73	0.00
		0.9	-7.07***	-8.73 - -5.41	0.00	-8.41	-31.75 - 14.93	1.00	13.74	-6.71 - 34.2	0.70	-6.98	-34.95 - 20.98	1.00	-40.84	-81.75 - 0.06	0.05	-11.16	-25.01 - 2.69	0.26
	Noncore	0.1	-5.44***	-7.63 - -3.24	0.00	-0.75	-34.91 - 33.41	1.00	-0.32	-17.79 - 17.14	1.00	-6.09	-60.24 - 48.07	1.00	32.66	-35.79 - 101.11	1.00	-10.47	-25.2 - 4.25	0.53
		0.5	-5.34***	-7.37 - -3.31	0.00	-35.12	-79.37 - 9.14	0.29	-5.46	-34.12 - 23.2	1.00	-44.32	-110.44 - 21.8	0.71	13.05	-67.32 - 93.42	1.00	-13.80*	-27.59 - -0.01	0.05
		0.9	-6.76***	-9.15 - -4.37	0.00	-11.57	-96.12 - 72.98	1.00	1.02	-43.66 - 45.7	1.00	-87.24*	-165.95 - -8.52	0.02	-13.84	-75.88 - 48.2	1.00	-15.82	-36.57 - 4.92	0.37

CPOE = computerized provider order entry, MU = meaningful use, CMI = case mix index, NCHS = national center for health statistics, CLABSI = central line associated blood stream infection, CAUTI = catheter associated urinary tract infection, SSI-Colon = surgical site infection following colon surgery, MRSA = methicillin resistant staph aureus, C diff = Clostridioides difficile.

©2020 Murphy ZR et al. *JAMA Network Open*.

eMethods. Data Considerations

This supplement details the considerations behind selecting 2016 as our single year to analyze, along with our selection of Meaningful Use (MU) performance measures and Hospital Value Based Purchasing (HVBP) components.

First considering MU measures, Supplement 4 shows the number of hospitals attesting to each MU performance measure in each year of the program. This table looks at continuous MU performance measures crosslinked between the 4 versions of criteria that have existed over the life of the MU program. As this table shows, there is a division between what criteria hospitals attested to, with relatively sharp delineations between pre-2014 and post-2014 attestations and 2014 itself as a transition year. A cohesive analysis would require choosing one of these sets and the contemporaneous outcome data for analysis. While the pre-2014 measures are fundamental, they became quickly saturated and the post-2014 measures may have more relevance to the current and future direction of EHR implementation. While we do not feel this in itself is enough of a reason to pursue one set over the other, the below considerations of the HVBP datasets show that the post-2014 timeframe is more amenable to the purpose of this project, and moreover including only CY 2016 is the best option.

Each of the four HVBP domains are composed of one to several components, each with performance periods in each fiscal year representing some previous timeframe. Supplement 5 illustrates the performance periods of each HVBP domain component since inception of the program by fiscal year.

The Engagement domain is composed of several components which have stayed largely constant over time. However, there are two complicating factors.

First, the Hospital Compare datasets for FY2016 and prior include only dimension point scores. These are the number of points awarded for each component, with 0 for being at or below an established baseline, 10 for being at or above an established threshold, and 1-9 points for achieving gradations between the baseline and threshold, which are then summed after also examining improvement since the baseline period and consistency across dimensions to yield the overall domain score. This discrete scale is less suited to analysis, and perhaps less interesting to analyze, than the percent of respondents selecting “top box” scores for each component. These continuous percentages, adjusted for patient-level factors detailed elsewhere, are available only in the FY2017 and FY 2018 datasets, representing CY 2015 and CY 2016 respectively. Thus, the most informative analysis may be performed by looking at the adjusted top-box percentages in 2015 and/or 2016.

Second, the Pain Management component is present in each dataset except for FY2018, when these questions were no longer asked. Conversely, the care transition domain was added in FY2018 and is not present in any other year. If we were to include both FY 2017 and FY 2018 in the analysis, the only way to include both of these components would be to impute their values during the year not asked. Given that this means 50% of the data for each of these components would be imputed, this presents a significant barrier to the validity of the model resulting from this outcome and thus we favor one year or the other and include the respective dimension for that year.

The Efficiency domain, consisting of the sole MSPB measure, is unchanged from its inclusion in the HVBP in FY2015. It has been reported by calendar year in the Hospital Compare datasets for much longer. This, then, gives us little direction in selecting years to analyze. However, in looking at the components of the domain scores themselves rather than the points awarded, we have believe that the MSPB may be a better outcome to analyze than the domain score. The Efficiency domain score represents points awarded for performing at, below, or between pre-established baseline and threshold scores. The MSPB measure itself represents each hospital’s average price-standardized, risk adjusted spending per care episode divided by the national median of spending per episode. Using this measure avoids the problem of a large percent of the hospitals having 0 points (since scoring below 50th percentile of the baseline period earns 0 points). Using the MSPB directly will allow us to model across the entire spectrum of hospitals.

The Safety domain is composed of PSI-90 (itself a combination of several measures of accidents, injuries, and other adverse events), HAI 1-6 (rates of various kinds of hospital acquired infections), and PC-01 (rate of elective delivery before 39 weeks). As seen in supplement 4, the performance periods for PSI-90 are less regular than those for the Engagement and Efficiency domains and overlap significantly. While FY2015 has PSI-90 performance periods are contemporaneous only with the pre-2014 MU measures, FY2016 and on have performance periods that include both pre-2014 and post-2014 MU measures. Thus, the PSI-90 outcome is not conducive to analysis with post-2014 MU measures. While PSI-90 for FY2015 may be analyzed with pre-2014 MU measures this is a departure from the above considerations favoring the analysis of CY2015 and/or CY2016 data, and thus may be suited to separate analysis in the interest of keeping the current project cohesive. HAI 1-4 as reported in FY2016 to FY2018 have performance periods in CY2014 to CY2016, and similarly HAI 5-6 and PC-01 have performance periods in CY2015 to CY2016 for FY 2017 and FY 2018 respectively. While HAI 1-6 are reported as standardized infection ratios, PC-01 is reported as an unadjusted percentage, making it less amenable to

©2020 Murphy ZR et al. *JAMA Network Open*.

analysis since we cannot adjust for patient-level characteristics as with all the other measures. Thus, HAI 1-6 are viable outcomes to analyze for CY2015 and CY2016 while PSI-90 and PC-01 are not amenable to this analysis.

The clinical care domain, while one of the more interesting to analyze, has issues similar to PSI-90. The performance periods for all three components (MORT-30-AMI, MORT-30-HF, and MORT-30-PN) are identical, but vary in duration between fiscal years and largely overlap. While FY2014 and FY 2015 performance periods are contemporaneous only with the pre-2014 MU measures and could be analyzed against them, this may be suited to a separate analysis similar to the considerations for PSI-90 above.

These considerations lead us to considering data during 2015 or 2016, including the post-2014 set of MU measures as predictors and outcomes including the 8 dimensions of HCAHPS, MSPB, and HAI 1-6. In terms of modeling this dataset as a cross-sectional analysis, there is no clear benefit or method for using multiple years of data. Given that 2016 is the most recent year and is thus most likely to be most applicable to the current state of the field, we decided to analyze CY 2016.

©2020 Murphy ZR et al. *JAMA Network Open*.

eFigure. Hospital Value-Based Purchasing Program (HVBP) Domain Component Performance Periods by Fiscal Year

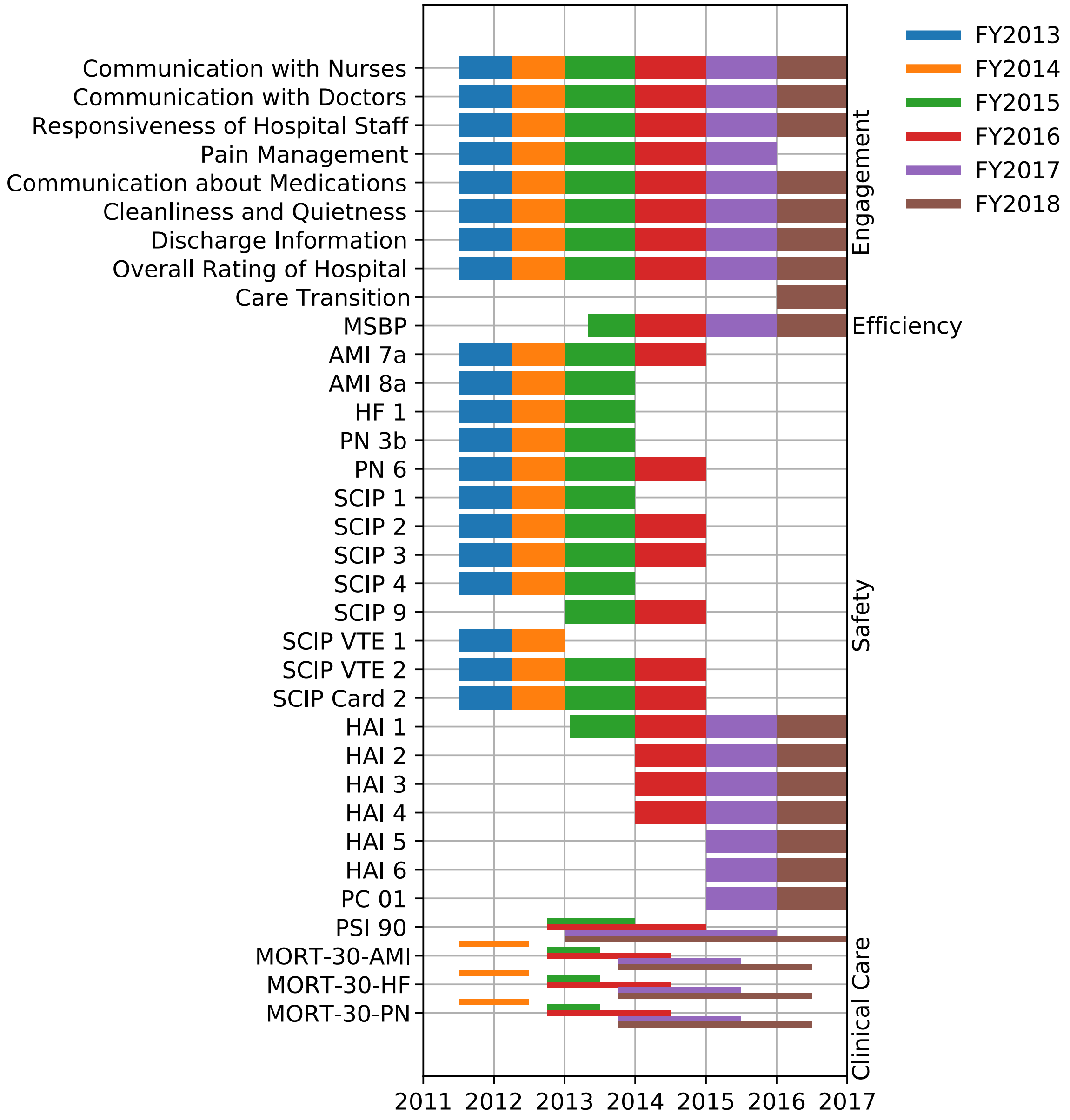

©2020 Murphy ZR et al. *JAMA Network Open*.
